# Association between mitochondria-related genes and systemic lupus erythematosus: Findings from Mendelian randomization study

**DOI:** 10.1097/MD.0000000000045301

**Published:** 2025-10-31

**Authors:** Xinglan Huang, Liehua Deng, Rongguo He, Suiying Zhang, Yuqiong Deng, Xiaoqing Zhao, Xingrong Wang, Yuqi Yang, Ning Zhang, Xiping Cheng

**Affiliations:** a Department of Dermatology, The First Affiliated Hospital of Jinan University, Jinan University & Jinan University Institute of Dermatology, Guangzhou, China; b Department of Dermatology, Guangzhou Twelfth People’s Hospital, Guangzhou, China; c Department of Dermatology, Dongguan Songshan Lake Central Hospital Affiliated to Guangdong Medical University, Dongguan, China; d Department of Dermatology, Panyu Maternal and Child Care Service Centre of Guangzhou, Guangzhou, China; e Department of Traditional Chinese Medicine, The Affiliated Traditional Chinese Medicine Hospital of Guangzhou Medical University, Guangzhou, China; f Department of Dermatology, Hui Ya Hospital of The First Affiliated Hospital of Sun Yat-sen University, Huizhou, China; g Department of Dermatology, The First Affiliated Hospital of Guangzhou Medical University, Guangzhou, China; h Department of Dermatology, Shenzhen Baoan Konghai Hospital, Guangzhou, China.

**Keywords:** gene expression, Mendelian randomization, methylation, mitochondrion, protein, systemic lupus erythematosus

## Abstract

Systemic lupus erythematosus (SLE) is a severe autoimmune disease that damages various organs of the body. Mitochondrial dysfunction is closely related to the pathogenesis of SLE, but the genetic pathophysiological mechanism underlying this association has not been fully elucidated. The current study aims to elucidate the relationships between mitochondria-related genes and SLE via Mendelian randomization analysis. The genetic association data for SLE were obtained from the IEU OPEN GWAS, comprising 7071,163 SNPs from 5201 cases and 9066 controls of European ancestry. Known mitochondrial-related genes in humans were obtained from the MitoCarta 3.0 database. Summary-level data on the methylation, expression, and protein abundance of mitochondria-related genes were obtained from studies of the corresponding methylation quantitative trait loci (mQTL), expression quantitative trait loci (eQTL), and protein quantitative trait loci (pQTL), respectively. Two-sample Mendelian randomization (TSMR) and summary-data-based Mendelian randomization (SMR) analyses were then performed on the screened data to determine the relationships between the molecular characteristics of mitochondria-related genes and SLE. Integration of the positive exposures obtained with TSMR and SMR revealed common results for 12 expression loci, 23 methylation loci, and 3 plasma protein loci. Among them, the mQTL, eQTL, and pQTL of SPATA20 (cg22450693) were all positively correlated (p_HEIDI > .05, p_SMR < .05, p_TSMR < .05). In addition, the SMR results between eQTLs and SLE in exposed skin tissues verified that SPATA20 is a protective factor against SLE. However, CASP9 (cg21858823, cg14078231) and MSRA (cg12810313, cg16773768) were the only mQTL- and eQTL-positive exposures, and no SNP data for CASP9 and MSRA were found in the pQTL database. Therefore, the causal relationship between pQTLs and SLE remains unclear. The findings of this study suggest that the mitochondrial-related SPATA20 gene may be regulated by epigenetic changes to reduce the risk of SLE, thus providing a basis for the prevention and intervention of SLE.

## 1. Introduction

Systemic lupus erythematosus (SLE) is a chronic and recurrent autoimmune disease characterized by severe and persistent inflammatory responses. It is estimated that the global incidence of SLE is approximately 5.14 cases per 100,000 person-years (range 1.4–15.13 cases per 100,000 person-years), with approximately 400,000 new cases per year.^[1]^ The symptoms of SLE are highly diverse and can affect 1 or more organs and systems, such as the skin, joints, kidneys, heart, and nervous system, which can lead to serious complications and even death.^[2]^ It is generally believed that multiple abnormalities of mitochondria, including functional changes, oxidative stress, genetic polymorphisms, mitochondrial DNA (mtDNA) mutations, and apoptotic pathways, are closely related to the pathogenesis of lupus.^[3]^ Recent studies have further refined this understanding: decreased mtDNA copy number and mitochondrial homeostasis imbalance have been observed in SLE patients^[3]^; Mendelian randomization (MR) analyses suggest a causal link between mtDNA variation and autoimmune diseases; and oxidized mtDNA has been shown to trigger inflammasome activation and gasdermin D oligomerization, thereby amplifying inflammatory responses in SLE. These findings underscore the central role of mitochondrial dysfunction and mtDNA-related immune activation in SLE pathophysiology.^[4,5]^ The interaction between mitochondria and SLE has therefore remained a focus of recent research.

As a damage-related molecular pattern, mtDNA can activate innate immune signaling pathways and trigger abnormal inflammatory responses.^[4,5]^ To date, more and more studies have reported that mtDNA genetic variation is associated with the occurrence of SLE.^[6–9]^ SLE occurs due to an interaction between genetics and the environment. The biological characteristics of this disease not only depend on nuclear DNA but are also closely related to mtDNA.^[10]^ In response to cellular stress, both nuclear and mitochondrial DNA can activate specific sensors that stimulate cytokine production and immune complex formation, thereby exacerbating systemic inflammation and contributing to organ damage.^[11]^ Alterations in mtDNA may lead to oxidative stress and inflammation, which are strongly associated with the pathogenesis of SLE.^[12]^ By modulating the chromatin structure, DNA methylation can intricately regulate the interaction between promoters and transcription factors, thereby encoding genes in the transcriptional machinery. It has been demonstrated that alterations in DNA methylation play a pivotal role in epigenetics, potentially leading to disruption of immune tolerance and persistent SLE.^[13,14]^ Therefore, despite the well-established central role of mitochondria in SLE pathogenesis, the specific impact of mitochondria-associated genes and their methylation on downstream effects of SLE remains unclear.

MR analysis is a powerful strategy that utilizes genetic variants as instrumental variables to enhance the inference of causal relationships between exposures and outcomes.^[15]^ This approach offers significant advantages over traditional observational studies due to its ability to mitigate confounding and reverse-causality bias.^[16]^ The fundamental strength lies in the random assignment of genetic variants at conception and their stability throughout an individual’s lifetime, which remains unaffected by disease onset. Consequently, employing genetic variation to investigate the association between exposure factors and disease enables more reliable inference of causality.

With the accumulation of data from large-scale Genome-Wide Association Studies (GWAS) and quantitative trait loci (QTL), researchers have gained insights into the intricate relationship between gene regulation and disease. Specifically, attention has been directed towards mitochondrial-related genes’ involvement in regulatory processes, as well as how disparities in DNA methylation, gene expression, and protein abundance influence SLE risk alongside other diseases.

In this study, we employed MR analysis to investigate the potential causal relationships between mitochondrial-related genes and SLE by integrating methylation, gene expression, and protein abundance data. The validity of MR analysis relies on 3 core assumptions: first, the genetic variants selected as instrumental variables must be strongly associated with the exposure of interest (relevance); second, these variants should be independent of any confounding factors that could bias the exposure–outcome relationship (independence); and third, the variants should affect the outcome solely through the exposure, without alternative biological pathways influencing the association (exclusion restriction). These assumptions provide the theoretical foundation for the analytical framework used in the present study.

## 2. Data and methods

### 2.1. Study design

**Figure 1.**
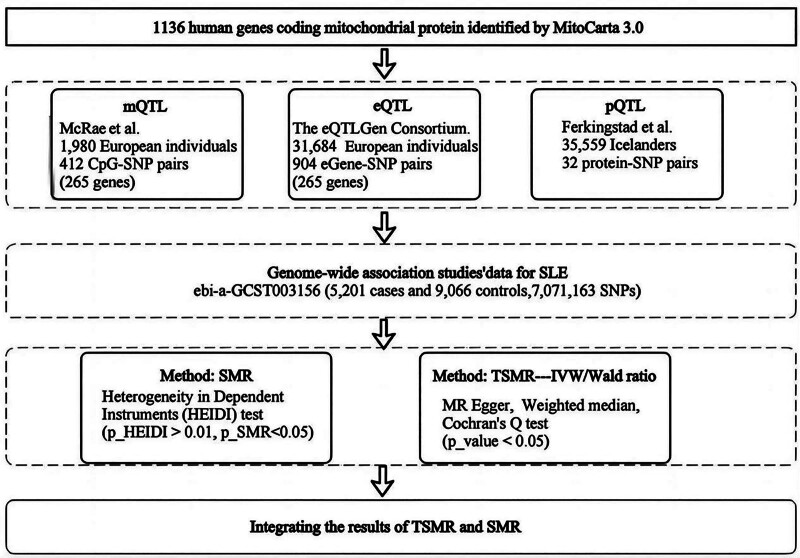
Schematic diagram of the study’s workflow. eQTLs = expression quantitative trait loci, IVW = inverse-variance weighted, mQTLs = methylation quantitative trait loci, pQTLs = protein quantitative trait loci, SLE = systemic lupus erythematosus, SMR = summary-data-based Mendelian randomization, TSMR = two-sample Mendelian randomization.

The flow chart of the study design is shown in Figure 1. First, we extracted instrumental variables for mitochondria-related genes at the methylation, gene expression, and protein abundance levels. Two-sample Mendelian randomization (TSMR) analyses were subsequently performed for SLE at each biological level. To strengthen causal inference, we also performed the analysis via summary-data-based Mendelian randomization (SMR). Finally, by integrating the results from the 3 biological levels of the TSMR and SMR analyses, we identified candidate causative genes. In addition, to further validate the candidate pathogenic genes, we also used the tissue-specific expression quantitative trait loci (eQTL) data of skin tissues, including Skin_Sun and Skin_Not_Sun, to evaluate the tissue-specific expression of target genes. All the methods used herein satisfy 3 core assumptions: genetic variation is strongly associated with exposure (i.e., association); there is no association between genetic variants and confounding factors (i.e., independence); and the effect of genetic variation on outcome is fully mediated by exposure (i.e., exclusion restrictions). The schematic diagram of the workflow is depicted in Figure 1.

### 2.2. Data acquisition and screening

The data reported in this study and the databases used in this study are shown in Table S1, Supplemental Digital Content, https://links.lww.com/MD/Q423. All databases were accessed in March 2024.

Mitochondria-related genes: we obtained data from the MitoCarta3.0 database (accessed on March 15, 2024).^[17]^ A total of 1136 known mitochondria-related genes were obtained (Table S2, Supplemental Digital Content, https://links.lww.com/MD/Q423).

Methylation quantitative trait loci (mQTL): the blood-based SNP-CpG associations were obtained from mQTL data on methylation, which was collected by McRae et al in a cohort comprising 1980 individuals of European descent.^[18]^ Cis-mQTLs of genetic variants that are closely related to the selected genes were extracted. Clumping was performed via plinkbinr (version 0.0.0.9000; https://github.com/gabraham/plinkbinr) based on the European 1000 Genomes Project data (https://www.internationalgenome.org/, accessed on March 15, 2024) to remove SNPs in linkage disequilibrium (clump_kb = 10,000, clump_r2 = 0.001). A stringent clump_r2 threshold of 0.001 was chosen to ensure the independence of instrumental variables and to minimize bias from residual linkage disequilibrium, thereby strengthening causal inference in MR analyses.

eQTL: the blood eQTL dataset was obtained from the eQTLGen consortium, comprising a total of 31,684 participants.^[19]^ We extracted genetic variants within 1000 kb flanking coding sequences (cis) that are closely related to gene expression. Among the cis-eQTLs, SNPs associated with the expression of transcripts associated with mitochondria-related genes were selected. All SNPs included in the initial analysis had at least one homozygous Psnp-mitodys < 5 e − 08.

Protein quantitative trait loci (pQTL): relevant data on genetic associations with levels of circulating proteins were obtained from a study conducted by Ferkingstad et al, in which a total of 28,191 genetic associations (*P*_value < 1.8e − 9) were identified for 4907 aptamers across 35,559 Icelanders.^[20]^

GWAS: SLE data were downloaded from IEU OPEN GWAS-related tools (https://gwas.mrcieu.ac.uk/, accessed on March 15, 2024). Seven European populations were identified, and the pre-analysis results revealed that ebi-a-GCST003156 had the best results. We identified 7071,163 SNPs across 5201 cases and 9066 controls. Quality control steps for GWAS data included excluding SNPs with a minor allele frequency < 0.01, removing insertion/deletion (Indel) variants, and retaining only biallelic SNPs. In addition, SNPs with low imputation quality (INFO score < 0.8) or high missingness (>5%) were excluded to ensure robust downstream MR analyses.

Skin tissue-specific expression of eQTL data: data were downloaded from the gene expression (GTEx) portal website (https://gtexportal.org/home/)^[21,22]^ to determine the skin tissue-specific expression of eQTLs. The GTEx database has 2 skin tissue datasets: skin-not sun exposed (suprapubic), including data from skin that is not exposed to the sun (such as the suprapubic area) and skin-sun exposed (lower leg), including data from skin (such as the lower leg area) exposed to the sun.

The quality control conditions for instrumental variables were as follows: we removed SNPs with *R*^2^ values > 0.9 and < 0.05 around the top SNP, and we retained only those SNPs with *R*^2^ values ≤ 0.9 among the remaining paired SNPs. Data from the 1000 Gene Project European population were selected to remove LD from GWAS data (source: https://ctg.cncr.nl/software/magma). Instrumental variables with *F* > 10 were calculated and retained. The harmonise_data package was used to standardize outcome and instrumental variables.

### 2.3. TSMR analysis

The R package TwoSampleMR (version 0.5.10; MRC Integrative Epidemiology Unit, University of Bristol, Bristol, United Kingdom)^[23]^ was used to conduct TSMR,^[24]^ direction testing and sensitivity analysis. The TwoSampleMR package contains multiple methods for MR. When the number of SNPs was ≥ 2, the TwoSampleMR package 5 default methods were used, including MR Egger, weighted median, inverse variance weighted (IVW), fixed effects and multiplicative random effects. The fixed effects model was used when there was no heterogeneity between the exposure and outcome, while the random effects model was used when there was heterogeneity. The exposure factors with *P*_values < .05 under the 2 methods were retained, and both a simple model and a weighted model were constructed to examine causal relationships. Results obtained via IVW have decisive significance; the results of the other 4 methods were only obtained for reference. In addition, when only 1 SNP remained, the Wald ratio could be used for MR analysis, and *P* < .05 indicated that there was a causal effect between the exposure factor and the outcome.

Odds ratios with 95% confidence intervals were used to estimate effect sizes. The Cochran *Q* test (mr_heterogeneity) was used for the IVW to evaluate heterogeneity among SNPs; a *P*_value < .05 indicated the presence of significant heterogeneity.^[25]^ The MR–Egger regression intercept and its 95% confidence interval were used to investigate the degree of bias in random estimates due to directional pleiotropy, and a *P*_value > .05 indicated the presence of no horizontal pleiotropy.^[26]^

### 2.4. SMR analysis

The SMR analysis was developed to explore pleiotropic associations between genetic features such as gene expression, DNA methylation, or protein abundance and important complex features such as disease phenotypes. To ensure adherence to MR principles in our analyses, causality was assessed in the following manner: βeQTL − IA/SAH/uIA = βSNP − IA/SAH/uIA/βSNP − eQTL. SMR is mainly based on SMR (Linux version 1.3.1; Yang Lab, School of Life Sciences, Westlake University, Hangzhou, China).^[27]^ When the exposure factor p_SMR < .05 and p_HEIDI > .01, the exposure can be considered positive, and there is evidence of colocalization.^[28]^

### 2.5. Integrating results from TSMR and SMR

To gain a comprehensive understanding of the correlations between mitochondrial-related genes and SLE at diverse levels of regulation, we integrated the results of TSMR and SMR after their merger and incorporated the outcomes from 3 distinct gene regulatory levels. Since proteins are the ultimate expression products of genes, we classified potential disease-causing genes into 3 tiers: Tier 1 genes are defined as those associated with SLE at the protein abundance level, as well as at the methylation and expression levels; Tier 2 genes are defined as those associated with SLE at the protein abundance level and either at the methylation or expression level; and Tier 3 genes are defined as those with an uncertain association with SLE at the protein abundance level, but a definite association with SLE at both the methylation and expression levels.

## 3. Results

### 3.1. MR analysis of mQTLs for mitochondria-related genes

A total of 645,45 SNPs were extracted, including 412 mitochondrial-related gene methylation site (cg) mQTLs, which were annotated to 264 genes (Table S3, Supplemental Digital Content, https://links.lww.com/MD/Q423). TSMR analysis revealed that 313 mitochondrial-related gene methylation site (cg) mQTLs were causally related to SLE (Table S4, Supplemental Digital Content, https://links.lww.com/MD/Q423). The positive exposures were then tested for heterogeneity and horizontal pleiotropy (Table S5, Supplemental Digital Content, https://links.lww.com/MD/Q423).

**Figure 2.**
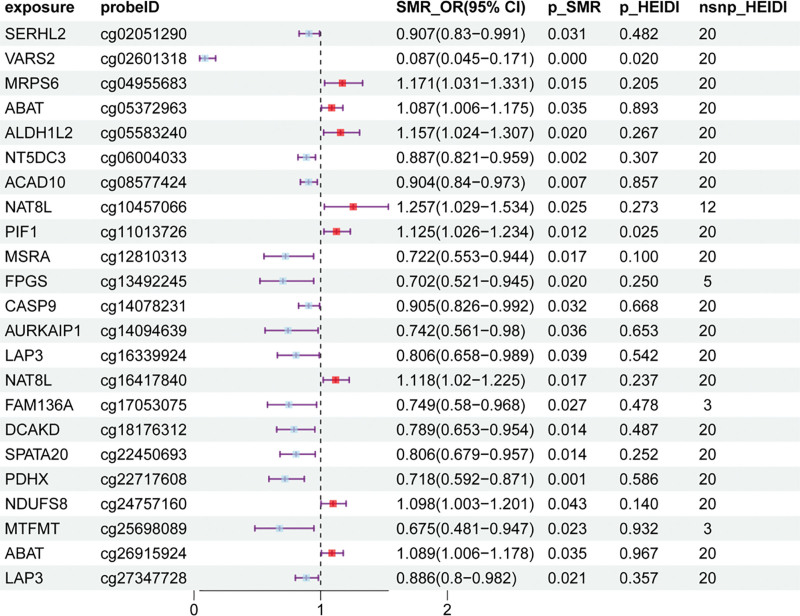
Forest plot showing the results of Mendelian randomization with respect to cis-mQTLs and SLE. mQTLs = methylation quantitative trait loci, SLE = systemic lupus erythematosus.

SMR analysis revealed that 39 mitochondria-related gene methylation loci (cg) in mQTLs were causatively related to SLE (Table S6, Supplemental Digital Content, https://links.lww.com/MD/Q423), and colocalization analysis yielded significant results (p_SMR < .05, p_HEIDI > .01). The 313 positive exposures elucidated via TSMR and the 39 positive exposures elucidated via SMR were intercrossed to obtain 23 mitochondrial-related gene methylation loci (cg) mQTLs. Twenty genes were annotated (ABAT, ACAD10, ALDH1L2, AURKAIP1, CASP9, DCAKD, FAM136A, FPGS, LAP3, MRPS6, MSRA, MTFMT, NAT8L, NDUFS8, NT5DC3, PDHX, PIF1, SERHL2, SPATA20, and VARS2). The results of these analyses were visualized via forest plots (Fig. 2). The positive exposures were subsequently tested for heterogeneity and horizontal pleiotropy (Table S7, Supplemental Digital Content, https://links.lww.com/MD/Q423). The results of the sensitivity analyses were visualized via scatterplots and funnel plots (Fig. S1, Supplemental Digital Content, https://links.lww.com/MD/Q424).

### 3.2. MR analysis of the eQTLs of mitochondria-related genes

A total of 2122 SNPs were extracted, including 904 eQTLs of mitochondria-related genes (Table S8, Supplemental Digital Content, https://links.lww.com/MD/Q423). The results of TSMR analysis revealed that 36 mitochondrial-related genes had a causal relationship with SLE (Table S9, Supplemental Digital Content, https://links.lww.com/MD/Q423). These 36 genes are listed in Table 1. The positive exposures were subsequently tested for heterogeneity and horizontal pleiotropy (Table S10, Supplemental Digital Content, https://links.lww.com/MD/Q423).

**Table 1:** Relationship between cis-eQTL of mitochondrial-related genes and SLE (results based on TSMR).

id.exposure	Exposure	Method	nsnp	*b*	se	pval	OR	or_uci95	or_lci95
eqtl-a-ENSG00000004142	POLDIP2	IVW (fe)	2	0.179	0.090	.048	1.196	1.427	1.002
eqtl-a-ENSG00000004799	PDK4	IVW (fe)	3	−0.252	0.097	.010	0.778	0.941	0.642
eqtl-a-ENSG00000006282	SPATA20	IVW (fe)	4	0.077	0.037	.039	1.08	1.163	1.004
eqtl-a-ENSG00000013583	HEBP1	IVW (fe)	3	−0.165	0.070	.019	0.848	0.973	0.739
eqtl-a-ENSG00000023228	NDUFS1	IVW (fe)	2	−0.496	0.199	.013	0.609	0.899	0.413
eqtl-a-ENSG00000087088	BAX	IVW (fe)	2	0.152	0.074	.041	1.164	1.346	1.006
eqtl-a-ENSG00000087157	PGS1	IVW (fe)	3	−0.356	0.128	.005	0.701	0.9	0.546
eqtl-a-ENSG00000100290	BIK	IVW (fe)	2	0.911	0.313	.004	2.487	4.589	1.348
eqtl-a-ENSG00000101574	METTL4	IVW (fe)	2	−0.358	0.150	.017	0.699	0.939	0.521
eqtl-a-ENSG00000102763	VWA8	IVW (fe)	2	−0.321	0.134	.016	0.725	0.943	0.558
eqtl-a-ENSG00000106153	CHCHD2	IVW (fe)	4	0.101	0.040	.011	1.106	1.195	1.024
eqtl-a-ENSG00000116688	MFN2	IVW (fe)	5	0.101	0.051	.046	1.107	1.223	1.002
eqtl-a-ENSG00000120832	MTERF2	IVW (fe)	2	−0.350	0.154	.023	0.705	0.953	0.521
eqtl-a-ENSG00000121691	CAT	IVW (fe)	3	−0.120	0.056	.032	0.887	0.99	0.794
eqtl-a-ENSG00000128050	PAICS	IVW (fe)	2	0.365	0.176	.038	1.44	2.033	1.02
eqtl-a-ENSG00000128311	TST	IVW (fe)	8	−0.175	0.067	.008	0.839	0.956	0.737
eqtl-a-ENSG00000132906	CASP9	IVW (fe)	3	−0.235	0.069	.001	0.791	0.905	0.691
eqtl-a-ENSG00000134326	CMPK2	IVW (mre)	3	0.852	0.365	.020	2.345	4.798	1.146
eqtl-a-ENSG00000135423	GLS2	IVW (fe)	2	0.693	0.281	.013	2	3.467	1.154
eqtl-a-ENSG00000136783	NIPSNAP3A	IVW (fe)	3	−0.353	0.158	.026	0.702	0.958	0.515
eqtl-a-ENSG00000137411	VARS2	IVW (mre)	3	−0.510	0.100	.000	0.6	0.731	0.493
eqtl-a-ENSG00000139531	SUOX	IVW (fe)	4	−0.158	0.056	.004	0.854	0.952	0.766
eqtl-a-ENSG00000143198	MGST3	IVW (fe)	5	−0.096	0.044	.027	0.908	0.989	0.834
eqtl-a-ENSG00000143252	SDHC	IVW (fe)	2	0.187	0.078	.017	1.206	1.406	1.034
eqtl-a-ENSG00000148459	PDSS1	IVW (fe)	2	0.175	0.066	.008	1.191	1.355	1.047
eqtl-a-ENSG00000156411	ATP5MPL	IVW (fe)	2	−0.155	0.068	.023	0.857	0.979	0.749
eqtl-a-ENSG00000171953	ATPAF2	IVW (fe)	2	0.163	0.064	.011	1.177	1.333	1.039
eqtl-a-ENSG00000175806	MSRA	IVW (fe)	9	−0.232	0.048	.000	0.793	0.871	0.722
eqtl-a-ENSG00000177302	TOP3A	IVW (fe)	2	0.214	0.091	.018	1.238	1.479	1.037
eqtl-a-ENSG00000196850	PPTC7	IVW (fe)	4	0.282	0.129	.029	1.326	1.707	1.029
eqtl-a-ENSG00000197119	SLC25A29	IVW (fe)	3	−0.216	0.067	.001	0.806	0.919	0.707
eqtl-a-ENSG00000198130	HIBCH	IVW (fe)	4	−0.265	0.062	.000	0.767	0.865	0.68
eqtl-a-ENSG00000203667	COX20	IVW (fe)	2	−0.166	0.080	.037	0.847	0.99	0.724
eqtl-a-ENSG00000204564	C6orf136	IVW (fe)	2	−0.416	0.143	.004	0.659	0.873	0.498
eqtl-a-ENSG00000241878	PISD	IVW (fe)	6	−0.100	0.043	.020	0.905	0.984	0.831
eqtl-a-ENSG00000254858	MPV17L2	IVW (fe)	2	−0.465	0.131	.000	0.628	0.811	0.486

eQTLs = expression quantitative trait loci, IVW = inverse-variance weighted, OR = odds ratio, pval = *P*-value, SE = standard error, SLE = systemic lupus erythematosus, TSMR = two-sample Mendelian randomization.

**Figure 3.**
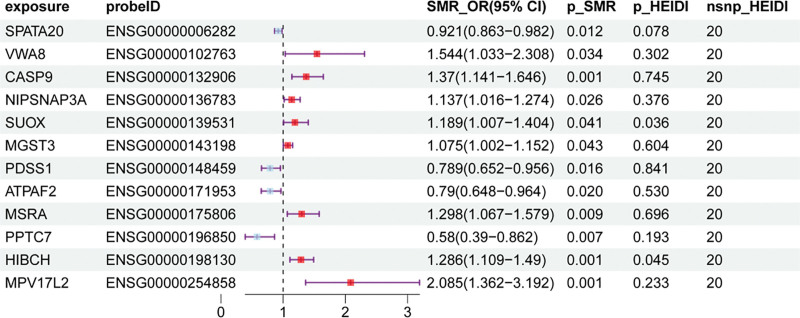
Forest plot of the results of Mendelian randomization with respect to cis-eQTLs and SLE. eQTLs = expression quantitative trait loci, SLE = systemic lupus erythematosus.

SMR analysis revealed that 65 mitochondria-related genes had causal relationships with SLE (Table S11, Supplemental Digital Content, https://links.lww.com/MD/Q423). Furthermore, colocalization analysis was performed and yielded significant results (p_SMR < .05, p_HEIDI > .01).^[28]^ The 36 positive exposures elucidated via TSMR and the 65 positive exposures elucidated via SMR were intercrossed. Twelve target mitochondria-related genes (SPATA20, VWA8, CASP9, NIPSNAP3A, SUOX, MGST3, PDSS1, ATPAF2, MSRA, PPTC7, HIBCH, and MPV17L2) were obtained. A forest map was used for visualization (Fig. 3). The results of the sensitivity analyses were visualized via forest plots, scatterplots and funnel plots (Fig. S2, Supplemental Digital Content, https://links.lww.com/MD/Q424).

### 3.3. MR analysis of the mitochondria-associated plasma protein pQTLs

A total of 110 SNPs were extracted, including 32 mitochondria-related plasma protein pQTLs (Table S12, Supplemental Digital Content, https://links.lww.com/MD/Q423). The results of TSMR analysis revealed that 4 mitochondria-related plasma protein pQTLs were causally related to SLE (Table 2 and Table S13, Supplemental Digital Content, https://links.lww.com/MD/Q423). The positive exposures were then tested for heterogeneity and horizontal pleiotropy (Table S14, Supplemental Digital Content, https://links.lww.com/MD/Q423).

**Table 2:** Relationship between pQTLs of mitochondrial-related genes and SLE (results based on TSMR).

id.exposure	Exposure	Method	nsnp	*b*	se	pval	OR	or_uci95	or_lci95
10630_5_HTATIP2_HTAI2_filtered	HTATIP2	IVW(fe)	4	0.212	0.084	.012	1.237	1.459	1.048
11117_2_SPATA20_SPT20_filtered	SPATA20	IVW(fe)	3	0.240	0.100	.016	1.271	1.546	1.045
12396_19_HIBCH_HIBCH_filtered	HIBCH	IVW(fe)	3	−0.247	0.119	.038	0.781	0.986	0.618
9126_171_NT5DC3_NT5D3	NT5DC3	IVW(fe)	2	0.519	0.163	.001	1.680	2.313	1.220

pQTLs = protein quantitative trait loci, IVW = inverse-variance weighted, OR = odds ratio, pval = *P*-value, SE = standard error, SLE = systemic lupus erythematosus, TSMR = two-sample Mendelian randomization.

**Figure 4.**
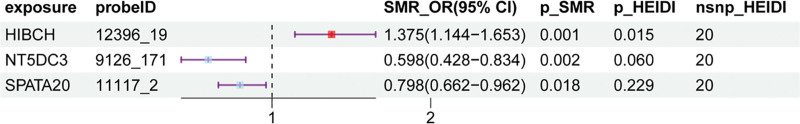
Forest plot of the results of Mendelian randomization with respect to cis-pQTLs and SLE. pQTLs = protein quantitative trait loci, SLE = systemic lupus erythematosus.

SMR analysis revealed that 12 mitochondria-related plasma protein pQTLs were found to have causal relationship with SLE (Table S15, Supplemental Digital Content, https://links.lww.com/MD/Q423), and colocation analysis yielded significant results (p_SMR < .05, p_HEIDI > .01). Four positive exposures obtained by TSMR analysis and 12 positive exposures obtained by SMR analysis were intercrossed to obtain 3 mitochondria-related plasma protein pQTLs (SPATA20, HIBCH, and NT5DC3), which were visualized via forest plots (Fig. 4). The results of the sensitivity analyses were visualized via forest plots, scatterplots and funnel plots (Fig. S3, Supplemental Digital Content, https://links.lww.com/MD/Q424).

### 3.4. Integrating the results of TSMR and SMR

**Figure 5.**
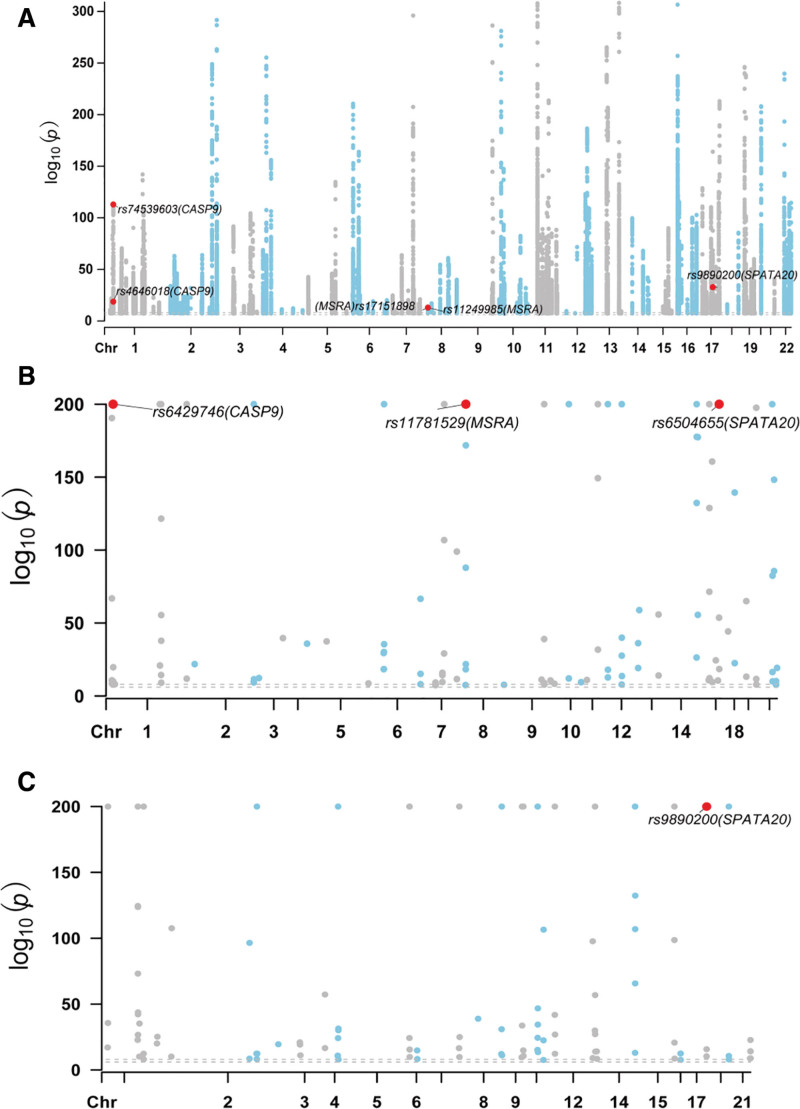
Manhattan plot for associations between mitochondrial-related gene molecular features and inflammatory bowel disease. Manhattan plot for mitochondrial-related gene methylation (A), expression (B), and protein abundance (C). Genes with significant signals in protein abundance levels were labeled.

Following the integration of results from TSMR and SMR, we identified 3 genes with multi-omics evidence for their associations with SLE. The mQTLs, eQTLs, and pQTLs of SPATA20 (cg22450693) were all found to be positive exposures by integrating the results of SLE with mQTLs, eQTLs, and pQTLs. Therefore, SPATA20 was considered the first-level gene based on multiomics evidence. The mQTLs of CASP9 (cg21858823, cg14078231) and MSRA (cg12810313, cg16773768) were risk factors for SLE, and eQTLs were protective factors against SLE. However, the SNP data of pQTLs were not obtained, and thus, the causal relationship between pQTLs and SLE remains unclear. Therefore, CASP9 and MSRA were used as third-level genes for multiomics evidence (Table 3). In addition, the Manhattan plot was used to show the associations between the mQTLs, eQTLs, and pQTLs of SPATA20, CASP9, and MSRA and SLE (Fig. 5).

**Table 3:** Tier of genetically predicted methylation, expression, and protein of candidate gene with SLE in Mendelian randomization analysis.

Exposure	Tier	mQTL			eQTL		pQTL	
		probeID	SMR_OR(95% CI)	p_SMR	SMR_OR (95% CI)	p_SMR	SMR_OR (95% CI)	p_SMR
MSRA	Tier3	cg12810313	0.722 (0.553–0.944)	.017	1.298 (1.067–1.579)	.009	NA	NA
CASP9	Tier3	cg14078231	0.905 (0.826–0.992)	.032	1.37 (1.141–1.646)	.001	NA	NA
SPATA20	Tier1	cg22450693	0.806 (0.679–0.957)	.014	0.921 (0.863–0.982)	.012	0.798 (0.662–0.962)	.018

eQTLs = expression quantitative trait loci, mQTLs = methylation quantitative trait loci, OR = odds ratio, pQTLs = protein quantitative trait loci, SLE = systemic lupus erythematosus, SMR = summary-data-based Mendelian randomization.

### 3.5. GTEx tissue specificity validation

**Figure 6.**
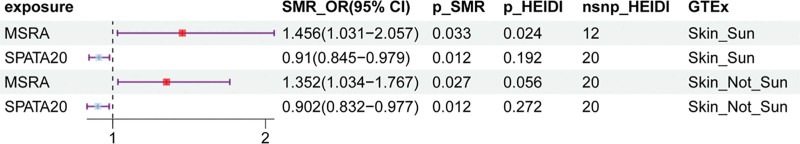
Forest plot of the results of Mendelian randomization with respect to skin eQTLs and SLE. eQTLs = expression quantitative trait loci, SLE = systemic lupus erythematosus.

Further analysis of the causal relationship between the eQTLs of skin tissue (Skin_Sun, Skin_Not_Sun) and SLE revealed that 2 SNPs were extracted, which were related only to MSRA and SPATA20 (Table S16, Supplemental Digital Content, https://links.lww.com/MD/Q423). SMR (Table S17, Supplemental Digital Content, https://links.lww.com/MD/Q423) results revealed that MSRA and SPATA20 in the skin were positive for SLE exposure (Fig. 6).

## 4. Discussion

MR is a frequently employed technique to establish causality, assuming specific conditions such as correlation, independence, and exclusion restrictions. In this study, 2 methods were utilized to showcase the impact of genes related to mitochondria on SLE. In this study, we performed TSMR and SMR analyses to explore the associations of genetically predicted levels of methylation, expression, and protein abundance of mitochondria-related genes with SLE. By integrating multiomics evidence, we found that the mitochondrial SPATA20 (spermatogenesis-associated protein 20) gene was negatively correlated with the risk of SLE and that the mitochondrial CASP9 (caspase-9) and MSRA (methionine sulfoxide reductase A) genes might be associated with SLE.

The mitochondrial-related SPATA20 gene is a spermatogenesis-related gene. The protein encoded by this gene is expressed in mammalian germ cells and plays an important role in spermatogenesis, sperm maturation, and fertilization.^[29]^ Although the SPATA20 gene is mainly related to spermatogenesis, studies have shown that the SPATA20 protein is related to mitochondrial function and energy metabolism during spermatogenesis and may be involved in the regulation of mitochondrial DNA replication, transcription or stability, thereby regulating mitochondrial function. SPATA20 is involved in acrosome formation and maintains mitochondrial function during spermatogenesis. In obese mice, SPATA 20^[30]^ was found to be involved in the response to DNA damage in sperm, thereby affecting sperm quality.^[31]^ Studies have shown that SPATA-20 knockout mice have reduced sperm number, motility and abnormal sperm morphology,^[32,33]^ thus suggesting that the SPATA20 gene may play a role in protecting sperm. Additionally, thioredoxin catalyzes protein disulfide bonds and regulates enzymes and transcription factors involved in antioxidant defence. Compared with that in healthy controls, the expression of SPATA-20 is increased 5-fold in the spermatogenic cells of patients with type 1 diabetes,^[34]^ and SPATA-20 in sperm may play a protective role in the oxidative stress environment associated with obesity.^[35]^ An increase in SPATA 20 may represent a compensatory response to the potential oxidative stress of a high-fat diet. Therefore, SPATA-20 plays a role in acrosome formation, spermatogenesis and sperm motility, maintenance of mitochondrial function, and compensation for the oxidative stress response. However, the correlation between mitochondrial SPATA20 and SLE has not been studied in the literature thus far. By using the SMR and TSMR methods, we found that the methylation level, expression and corresponding protein expression of the mitochondrial-related gene SPATA20 were negatively correlated with the risk of SLE. SPATA20 encodes a spermatogenesis-associated protein that localizes to the mitochondria and participates in acrosome formation, maintenance of mitochondrial structure, and regulation of oxidative stress responses. Beyond its role in reproductive biology, accumulating evidence suggests that SPATA20 may contribute to mitochondrial DNA stability and energy homeostasis through thioredoxin-like activity and interactions with antioxidant defense pathways. In the context of SLE, where excessive mitochondrial reactive oxygen species production, mtDNA leakage, and activation of innate immune pathways are key pathological features, enhanced SPATA20 function could theoretically mitigate oxidative stress, preserve mitochondrial membrane potential, and reduce the release of pro-inflammatory mitochondrial signals such as oxidized mtDNA.

The present multi-omics findings – with consistent associations in methylation (mQTL), gene expression (eQTL), and protein abundance (pQTL) analyses – support a model in which SPATA20 acts as a systemic protective factor, potentially by modulating mitochondrial resilience to immune-mediated oxidative injury. Future functional studies in immune cells and lupus animal models are warranted to clarify whether targeted enhancement of SPATA20 activity could represent a novel therapeutic strategy in SLE.

CASP9, namely, caspase 9, is one of the members of the caspase family encoding caspases and is known as the promoter of the mitochondrial intrinsic apoptosis pathway caspases or an adapter of apoptosis-dependent receptors.^[36–38]^ Mitochondria are the control centers of apoptosis, and dissipation of their transmembrane potential is a key step in apoptosis. Cytochrome C (Cyt-C) is released from mitochondria when cells are stimulated by DNA damage, cellular stress or hypoxia. Cyt-C binds to Apaf-1 (apoptotic protease activating factor-1) and ATP to form multimers, which in turn activate Caspase 9. Activated Caspase 9 forms heterodimers that further activate downstream Caspase 3, which triggers apoptosis. Studies have shown that apoptosis is increased in patients with SLE, and both apoptosis and autoantibodies are important factors related to disease activity in the pathogenesis of SLE.^[39]^ These findings are consistent with our findings that the mitochondria-related gene CASP9 is negatively correlated with SLE risk.

MSRA encodes a ubiquitous and highly conserved protein that is responsible for the enzymatic reduction of methionine sulfoxide to methionine. Human and animal studies have shown that methionine sulfoxide reductase A is expressed at the highest level in kidney and nerve tissues and functions to repair oxidative damage.^[40,41]^ Oxidative stress is closely related to the pathogenesis of SLE. It has been reported that oxidative stress leads to impaired mitochondrial function, which in turn produces reactive oxygen species and triggers autoantigenicity and proinflammatory cytokines, leading to SLE.^[42,43]^ To date, no studies have investigated the relationship between methionine sulfoxide reductase A and SLE. Our study suggests that the mitochondrial-associated gene MSRA may be associated with the risk of SLE, possibly through decreased expression of methionine sulfoxide reductase A, which reduces proteins that repair mitochondrial oxidative damage, further exacerbates oxidative stress and triggers autoantigenicity and proinflammatory cytokines, thus leading to SLE.

MR can effectively avoid reverse causality and confounding factors. Our study has the following strengths. First, we used GWAS data, MitoCarta 3.0 data, as well as eQTL, mQTL and pQTL data to investigate the causal relationships between mitochondria-related genes and SLE at the methylation, expression and protein abundance levels. Second, we found a positive association between mitochondria-related genes and SLE risk based on genetic data. Finally, multiple methods were used to validate the accuracy of the MR results, including SMR, TSMR, and multiomic analysis. Furthermore, the results were verified in in exposed and nonexposed skin tissue.

However, this study also has several limitations. First, the eQTLs of CASP9 (cg21858823, cg14078231) and MSRA (cg12810313, cg16773768) were risk factors for SLE, whereas their mQTLs were protective factors. The abnormal expression of the CASP9 and MSRA genes may increase the risk of SLE, while their DNA methylation may alleviate disease occurrence. However, no suitable CASP9 or MSRA SNP data were available in the pQTL database; therefore, the causal relationship between pQTLs and SLE remains unclear. Second, although we performed multiple sensitivity analyses to evaluate the robustness of the MR results, MR analysis cannot completely eliminate the possibility of genetic confounding, as discussed by Davey Smith et al,^[43]^ and this inherent limitation should be acknowledged when interpreting causal inferences. Finally, the data used in this study were derived from mitochondrial-related genes in individuals of European ancestry, which may limit the generalizability of our findings to other populations. To address this, future studies will aim to validate these results in multi-ethnic cohorts, thereby improving the robustness and extrapolation of the conclusions across diverse genetic backgrounds.

In conclusion, our data revealed that SPATA20 was negatively associated with the risk of SLE, whereas CASP9 and MSRA gene expression and methylation played important roles in the pathogenesis of SLE. These findings are based on multiomic evidence from MR. The present MR study explored the potential causal relationship between mitochondrial-related gene methylation, expression and protein abundance and SLE and revealed that SPATA20, CASP9 and MSRA mitochondrial-related genes were correlated with SLE. Furthermore, this study revealed the importance of the regulation of these genes in the pathogenesis of SLE. However, further clinical studies are needed to confirm this work and explore the possible mechanisms.

## Acknowledgments

The authors thank the studies or consortiums referenced and included in the present analysis for providing public datasets.

## Author contributions

**Conceptualization:** Xinglan Huang, Liehua Deng, Rongguo He, Suiying Zhang, Xiaoqing Zhao, Ning Zhang, Xiping Cheng.

**Data curation:** Xinglan Huang, Liehua Deng, Rongguo He, Suiying Zhang, Xiaoqing Zhao, Xiping Cheng.

**Formal analysis:** Xinglan Huang, Liehua Deng, Yuqiong Deng, Xiaoqing Zhao, Xingrong Wang, Ning Zhang, Xiping Cheng.

**Investigation:** Xinglan Huang, Liehua Deng, Suiying Zhang, Yuqiong Deng, Xiaoqing Zhao, Yuqi Yang, Xiping Cheng.

**Methodology:** Xinglan Huang, Liehua Deng, Yuqiong Deng, Xiaoqing Zhao, Yuqi Yang, Xiping Cheng.

**Supervision:** Rongguo He, Suiying Zhang, Yuqiong Deng, Xiaoqing Zhao, Xiping Cheng.

**Validation:** Rongguo He, Suiying Zhang, Yuqiong Deng, Xiaoqing Zhao, Xiping Cheng.

**Visualization:** Xinglan Huang, Suiying Zhang, Yuqiong Deng, Xiaoqing Zhao, Xingrong Wang, Yuqi Yang, Xiping Cheng.

**Writing – original draft:** Xinglan Huang, Xiping Cheng.

**Writing – review & editing:** Xinglan Huang, Xiping Cheng.

## Supplementary Material




