# Supplementary material for: Association between mitochondria-related genes and systemic lupus erythematosus: Findings from Mendelian randomization study
Source: Medicine (Baltimore). 2025 Oct 31;104(44):e45301. doi: 10.1097/MD.0000000000045301 (PMC12582722; doi:10.1097/MD.0000000000045301)
Supplement: Supplementary file 1 [file medi-104-e45301-s001.pdf]

Table S1. Information of included studies and consortia

Exposure/Outcome	Consortium/First author	Participants	Pubmed ID/Web source
mQTL	McRae et al.	1,980 European individuals	30514905
eQTL	eQTLGen Consortium	31,684 individuals (majority of samples were of European ancestry)	https://www.eqtlgen.org/cis-eqtls.html
Tissue-specific eQTL	The Genotype-Tissue Expression project	838 individuals (85.5% were European)	https://gtexportal.org/home/datasets
pQTL	Ferkingstad et al.	35,559 Icelanders	34857953
Systemic lupus erythematosus	IEU OPEN GWAS summary data	5201 European-ancestry cases and 9066 European-ancestry controls	https://gwas.mrcieu.ac.uk/datasets/ebi-a-GCST003156/

QTL, quantitative trait loci

Table S2. A total of 1136 known mitochondria-related genes.

HumanGeneID	Symbol	Description	MitoCarta3.0_List
1537	CYC1	cytochrome c1	MitoCarta3.0
6390	SDHB	succinate dehydrogenase complex iron sulfur subunit B	MitoCarta3.0
10229	COQ7	coenzyme Q7, hydroxylase	MitoCarta3.0
6389	SDHA	succinate dehydrogenase complex flavoprotein subunit A	MitoCarta3.0
7384	UQCRC1	ubiquinol-cytochrome c reductase core protein 1	MitoCarta3.0
84274	COQ5	coenzyme Q5, methyltransferase	MitoCarta3.0
5160	PDHA1	pyruvate dehydrogenase E1 subunit alpha 1	MitoCarta3.0
57017	COQ9	coenzyme Q9	MitoCarta3.0
6182	MRPL12	mitochondrial ribosomal protein L12	MitoCarta3.0
513	ATP5F1D	ATP synthase F1 subunit delta	MitoCarta3.0
9377	COX5A	cytochrome c oxidase subunit 5A	MitoCarta3.0
122961	ISCA2	iron-sulfur cluster assembly 2	MitoCarta3.0
9512	PMPCB	peptidase, mitochondrial processing subunit beta	MitoCarta3.0
7386	UQCRFS1	ubiquinol-cytochrome c reductase, Rieske iron-sulfur polypeptide 1	MitoCarta3.0
498	ATP5F1A	ATP synthase F1 subunit alpha	MitoCarta3.0
4967	OGDH	oxoglutarate dehydrogenase	MitoCarta3.0
5162	PDHB	pyruvate dehydrogenase E1 subunit beta	MitoCarta3.0
7385	UQCRC2	ubiquinol-cytochrome c reductase core protein 2	MitoCarta3.0
6392	SDHD	succinate dehydrogenase complex subunit D	MitoCarta3.0
60488	MRPS35	mitochondrial ribosomal protein S35	MitoCarta3.0
27089	UQCRQ	ubiquinol-cytochrome c reductase complex III subunit VII	MitoCarta3.0
116540	MRPL53	mitochondrial ribosomal protein L53	MitoCarta3.0
1629	DBT	dihydrolipoamide branched chain transacylase E2	MitoCarta3.0
5166	PDK4	pyruvate dehydrogenase kinase 4	MitoCarta3.0
4191	MDH2	malate dehydrogenase 2	MitoCarta3.0
23107	MRPS27	mitochondrial ribosomal protein S27	MitoCarta3.0
1431	CS	citrate synthase	MitoCarta3.0
80273	GRPEL1	GrpE like 1, mitochondrial	MitoCarta3.0
1737	DLAT	dihydrolipoamide S-acetyltransferase	MitoCarta3.0
10128	LRPPRC	leucine rich pentatricopeptide repeat containing	MitoCarta3.0
1743	DLST	dihydrolipoamide S-succinyltransferase	MitoCarta3.0
8050	PDHX	pyruvate dehydrogenase complex component X	MitoCarta3.0
85476	GFM1	G elongation factor mitochondrial 1	MitoCarta3.0
25874	MPC2	mitochondrial pyruvate carrier 2	MitoCarta3.0
4719	NDUFS1	NADH:ubiquinone oxidoreductase core subunit S1	MitoCarta3.0
26589	MRPL46	mitochondrial ribosomal protein L46	MitoCarta3.0
514	ATP5F1E	ATP synthase F1 subunit epsilon	MitoCarta3.0
5250	SLC25A3	solute carrier family 25 member 3	MitoCarta3.0
51649	MRPS23	mitochondrial ribosomal protein S23	MitoCarta3.0
2271	FH	fumarate hydratase	MitoCarta3.0
23203	PMPCA	peptidase, mitochondrial processing subunit alpha	MitoCarta3.0
506	ATP5F1B	ATP synthase F1 subunit beta	MitoCarta3.0
135154	SDHAF4	succinate dehydrogenase complex assembly factor 4	MitoCarta3.0
29796	UQCR10	ubiquinol-cytochrome c reductase, complex III subunit X	MitoCarta3.0
81689	ISCA1	iron-sulfur cluster assembly 1	MitoCarta3.0
8803	SUCLA2	succinate-CoA ligase ADP-forming subunit beta	MitoCarta3.0
51805	COQ3	coenzyme Q3, methyltransferase	MitoCarta3.0
55699	IARS2	isoleucyl-tRNA synthetase 2, mitochondrial	MitoCarta3.0
64960	MRPS15	mitochondrial ribosomal protein S15	MitoCarta3.0
3419	IDH3A	isocitrate dehydrogenase (NAD(+)) 3 catalytic subunit alpha	MitoCarta3.0
1353	COX11	cytochrome c oxidase copper chaperone COX11	MitoCarta3.0
2110	ETFDH	electron transfer flavoprotein dehydrogenase	MitoCarta3.0
26519	TIMM10	translocase of inner mitochondrial membrane 10	MitoCarta3.0
64981	MRPL34	mitochondrial ribosomal protein L34	MitoCarta3.0
51069	MRPL2	mitochondrial ribosomal protein L2	MitoCarta3.0
593	BCKDHA	branched chain keto acid dehydrogenase E1 subunit alpha	MitoCarta3.0
7388	UQCRH	ubiquinol-cytochrome c reductase hinge protein	MitoCarta3.0
192286	HIGD2A	HIG1 hypoxia inducible domain family member 2A	MitoCarta3.0
539	ATP5PO	ATP synthase peripheral stalk subunit OSCP	MitoCarta3.0
1892	ECHS1	enoyl-CoA hydratase, short chain 1	MitoCarta3.0
25875	LETMD1	LETM1 domain containing 1	MitoCarta3.0
1337	COX6A1	cytochrome c oxidase subunit 6A1	MitoCarta3.0
1355	COX15	cytochrome c oxidase assembly homolog COX15	MitoCarta3.0
10939	AFG3L2	AFG3 like matrix AAA peptidase subunit 2	MitoCarta3.0
3030	HADHA	hydroxyacyl-CoA dehydrogenase trifunctional multienzyme complex subunit alpha	MitoCarta3.0
2108	ETFA	electron transfer flavoprotein subunit alpha	MitoCarta3.0
374291	NDUFS7	NADH:ubiquinone oxidoreductase core subunit S7	MitoCarta3.0
1376	CPT2	carnitine palmitoyltransferase 2	MitoCarta3.0
594	BCKDHB	branched chain keto acid dehydrogenase E1 subunit beta	MitoCarta3.0
3420	IDH3B	isocitrate dehydrogenase (NAD(+)) 3 non-catalytic subunit beta	MitoCarta3.0
23395	LARS2	leucyl-tRNA synthetase 2, mitochondrial	MitoCarta3.0
35	ACADS	acyl-CoA dehydrogenase short chain	MitoCarta3.0
3954	LETM1	leucine zipper and EF-hand containing transmembrane protein 1	MitoCarta3.0
521	ATP5ME	ATP synthase membrane subunit e	MitoCarta3.0
4976	OPA1	OPA1 mitochondrial dynamin like GTPase	MitoCarta3.0
549	AUH	AU RNA binding methylglutaconyl-CoA hydratase	MitoCarta3.0
8802	SUCLG1	succinate-CoA ligase GDP/ADP-forming subunit alpha	MitoCarta3.0
4729	NDUFV2	NADH:ubiquinone oxidoreductase core subunit V2	MitoCarta3.0
51004	COQ6	coenzyme Q6, monooxygenase	MitoCarta3.0
84545	MRPL43	mitochondrial ribosomal protein L43	MitoCarta3.0
83451	ABHD11	abhydrolase domain containing 11	MitoCarta3.0
522	ATP5PF	ATP synthase peripheral stalk subunit F6	MitoCarta3.0
4714	NDUFB8	NADH:ubiquinone oxidoreductase subunit B8	MitoCarta3.0
9361	LONP1	lon peptidase 1, mitochondrial	MitoCarta3.0
1738	DLD	dihydrolipoamide dehydrogenase	MitoCarta3.0
9131	AIFM1	apoptosis inducing factor mitochondria associated 1	MitoCarta3.0
79746	ECHDC3	enoyl-CoA hydratase domain containing 3	MitoCarta3.0
139322	APOOL	apolipoprotein O like	MitoCarta3.0
124995	MRPL10	mitochondrial ribosomal protein L10	MitoCarta3.0
92399	MRRF	mitochondrial ribosome recycling factor	MitoCarta3.0
4728	NDUFS8	NADH:ubiquinone oxidoreductase core subunit S8	MitoCarta3.0
34	ACADM	acyl-CoA dehydrogenase medium chain	MitoCarta3.0
10989	IMMT	inner membrane mitochondrial protein	MitoCarta3.0
26520	TIMM9	translocase of inner mitochondrial membrane 9	MitoCarta3.0
291	SLC25A4	solute carrier family 25 member 4	MitoCarta3.0
25813	SAMM50	SAMM50 sorting and assembly machinery component	MitoCarta3.0
4720	NDUFS2	NADH:ubiquinone oxidoreductase core subunit S2	MitoCarta3.0
4723	NDUFV1	NADH:ubiquinone oxidoreductase core subunit V1	MitoCarta3.0
50	ACO2	aconitase 2	MitoCarta3.0
6832	SUPV3L1	Suv3 like RNA helicase	MitoCarta3.0
2235	FECH	ferrochelatase	MitoCarta3.0
4528	MTIF2	mitochondrial translational initiation factor 2	MitoCarta3.0
5245	PHB	prohibitin	MitoCarta3.0
26275	HIBCH	3-hydroxyisobutyryl-CoA hydrolase	MitoCarta3.0
51116	MRPS2	mitochondrial ribosomal protein S2	MitoCarta3.0
3313	HSPA9	heat shock protein family A (Hsp70) member 9	MitoCarta3.0
6834	SURF1	SURF1 cytochrome c oxidase assembly factor	MitoCarta3.0
10935	PRDX3	peroxiredoxin 3	MitoCarta3.0
27069	GHITM	growth hormone inducible transmembrane protein	MitoCarta3.0
60558	GUF1	GUF1 homolog, GTPase	MitoCarta3.0
26517	TIMM13	translocase of inner mitochondrial membrane 13	MitoCarta3.0
57128	LYRM4	LYR motif containing 4	MitoCarta3.0
54948	MRPL16	mitochondrial ribosomal protein L16	MitoCarta3.0
64976	MRPL40	mitochondrial ribosomal protein L40	MitoCarta3.0
3421	IDH3G	isocitrate dehydrogenase (NAD(+)) 3 non-catalytic subunit gamma	MitoCarta3.0
6391	SDHC	succinate dehydrogenase complex subunit C	MitoCarta3.0
4711	NDUFB5	NADH:ubiquinone oxidoreductase subunit B5	MitoCarta3.0
54949	SDHAF2	succinate dehydrogenase complex assembly factor 2	MitoCarta3.0
93058	COQ10A	coenzyme Q10A	MitoCarta3.0
8209	GATD3A	glutamine amidotransferase like class 1 domain containing 3A	MitoCarta3.0
25828	TXN2	thioredoxin 2	MitoCarta3.0
55168	MRPS18A	mitochondrial ribosomal protein S18A	MitoCarta3.0
1345	COX6C	cytochrome c oxidase subunit 6C	MitoCarta3.0
4715	NDUFB9	NADH:ubiquinone oxidoreductase subunit B9	MitoCarta3.0
23788	MTCH2	mitochondrial carrier 2	MitoCarta3.0
4700	NDUFA6	NADH:ubiquinone oxidoreductase subunit A6	MitoCarta3.0
788	SLC25A20	solute carrier family 25 member 20	MitoCarta3.0
65008	MRPL1	mitochondrial ribosomal protein L1	MitoCarta3.0
10469	TIMM44	translocase of inner mitochondrial membrane 44	MitoCarta3.0
10063	COX17	cytochrome c oxidase copper chaperone COX17	MitoCarta3.0
440574	MICOS10	mitochondrial contact site and cristae organizing system subunit 10	MitoCarta3.0
6648	SOD2	superoxide dismutase 2	MitoCarta3.0
1340	COX6B1	cytochrome c oxidase subunit 6B1	MitoCarta3.0
7416	VDAC1	voltage dependent anion channel 1	MitoCarta3.0
8192	CLPP	caseinolytic mitochondrial matrix peptidase proteolytic subunit	MitoCarta3.0
3033	HADH	hydroxyacyl-CoA dehydrogenase	MitoCarta3.0
33	ACADL	acyl-CoA dehydrogenase long chain	MitoCarta3.0
38	ACAT1	acetyl-CoA acetyltransferase 1	MitoCarta3.0
115416	MALSU1	mitochondrial assembly of ribosomal large subunit 1	MitoCarta3.0
10845	CLPX	caseinolytic mitochondrial matrix peptidase chaperone subunit X	MitoCarta3.0
4724	NDUFS4	NADH:ubiquinone oxidoreductase subunit S4	MitoCarta3.0
51073	MRPL4	mitochondrial ribosomal protein L4	MitoCarta3.0
708	C1QBP	complement C1q binding protein	MitoCarta3.0
10531	PITRM1	pitrilysin metallopeptidase 1	MitoCarta3.0
55245	UQCC1	ubiquinol-cytochrome c reductase complex assembly factor 1	MitoCarta3.0
128308	MRPL55	mitochondrial ribosomal protein L55	MitoCarta3.0
79922	MRM1	mitochondrial rRNA methyltransferase 1	MitoCarta3.0
51102	MECR	mitochondrial trans-2-enoyl-CoA reductase	MitoCarta3.0
65080	MRPL44	mitochondrial ribosomal protein L44	MitoCarta3.0
84263	HSDL2	hydroxysteroid dehydrogenase like 2	MitoCarta3.0
63931	MRPS14	mitochondrial ribosomal protein S14	MitoCarta3.0
11194	ABCB8	ATP binding cassette subfamily B member 8	MitoCarta3.0
91647	ATPAF2	ATP synthase mitochondrial F1 complex assembly factor 2	MitoCarta3.0
4704	NDUFA9	NADH:ubiquinone oxidoreductase subunit A9	MitoCarta3.0
388753	COA6	cytochrome c oxidase assembly factor 6	MitoCarta3.0
5442	POLRMT	RNA polymerase mitochondrial	MitoCarta3.0
23479	ISCU	iron-sulfur cluster assembly enzyme	MitoCarta3.0
84816	RTN4IP1	reticulon 4 interacting protein 1	MitoCarta3.0
55753	OGDHL	oxoglutarate dehydrogenase L	MitoCarta3.0
509	ATP5F1C	ATP synthase F1 subunit gamma	MitoCarta3.0
10295	BCKDK	branched chain keto acid dehydrogenase kinase	MitoCarta3.0
84340	GFM2	GTP dependent ribosome recycling factor mitochondrial 2	MitoCarta3.0
29078	NDUFAF4	NADH:ubiquinone oxidoreductase complex assembly factor 4	MitoCarta3.0
399512	SLC25A35	solute carrier family 25 member 35	MitoCarta3.0
8402	SLC25A11	solute carrier family 25 member 11	MitoCarta3.0
26521	TIMM8B	translocase of inner mitochondrial membrane 8 homolog B	MitoCarta3.0
27349	MCAT	malonyl-CoA-acyl carrier protein transacylase	MitoCarta3.0
200205	IBA57	iron-sulfur cluster assembly factor IBA57	MitoCarta3.0
11331	PHB2	prohibitin 2	MitoCarta3.0
7818	DAP3	death associated protein 3	MitoCarta3.0
56942	CMC2	C-X9-C motif containing 2	MitoCarta3.0
644096	SDHAF1	succinate dehydrogenase complex assembly factor 1	MitoCarta3.0
51373	MRPS17	mitochondrial ribosomal protein S17	MitoCarta3.0
137872	ADHFE1	alcohol dehydrogenase iron containing 1	MitoCarta3.0
9054	NFS1	NFS1 cysteine desulfurase	MitoCarta3.0
79133	NDUFAF5	NADH:ubiquinone oxidoreductase complex assembly factor 5	MitoCarta3.0
63875	MRPL17	mitochondrial ribosomal protein L17	MitoCarta3.0
51204	TACO1	translational activator of cytochrome c oxidase I	MitoCarta3.0
55669	MFN1	mitofusin 1	MitoCarta3.0
160760	PPTC7	protein phosphatase targeting COQ7	MitoCarta3.0
65003	MRPL11	mitochondrial ribosomal protein L11	MitoCarta3.0
1327	COX4I1	cytochrome c oxidase subunit 4I1	MitoCarta3.0
4726	NDUFS6	NADH:ubiquinone oxidoreductase subunit S6	MitoCarta3.0
28998	MRPL13	mitochondrial ribosomal protein L13	MitoCarta3.0
5019	OXCT1	3-oxoacid CoA-transferase 1	MitoCarta3.0
1329	COX5B	cytochrome c oxidase subunit 5B	MitoCarta3.0
5164	PDK2	pyruvate dehydrogenase kinase 2	MitoCarta3.0
211	ALAS1	5'-aminolevulinate synthase 1	MitoCarta3.0
9553	MRPL33	mitochondrial ribosomal protein L33	MitoCarta3.0
25821	MTO1	mitochondrial tRNA translation optimization 1	MitoCarta3.0
11019	LIAS	lipoic acid synthetase	MitoCarta3.0
4698	NDUFA5	NADH:ubiquinone oxidoreductase subunit A5	MitoCarta3.0
4712	NDUFB6	NADH:ubiquinone oxidoreductase subunit B6	MitoCarta3.0
10651	MTX2	metaxin 2	MitoCarta3.0
8801	SUCLG2	succinate-CoA ligase GDP-forming subunit beta	MitoCarta3.0
2230	FDX1	ferredoxin 1	MitoCarta3.0
6576	SLC25A1	solute carrier family 25 member 1	MitoCarta3.0
28957	MRPS28	mitochondrial ribosomal protein S28	MitoCarta3.0
92609	TIMM50	translocase of inner mitochondrial membrane 50	MitoCarta3.0
7417	VDAC2	voltage dependent anion channel 2	MitoCarta3.0
284439	SLC25A42	solute carrier family 25 member 42	MitoCarta3.0
54543	TOMM7	translocase of outer mitochondrial membrane 7	MitoCarta3.0
1891	ECH1	enoyl-CoA hydratase 1	MitoCarta3.0
5188	GATB	glutamyl-tRNA amidotransferase subunit B	MitoCarta3.0

617	BCS1L	BCS1 homolog, ubiquinol-cytochrome c reductase complex chaperone	MitoCarta3.0
26284	ERAL1	Era like 12S mitochondrial rRNA chaperone 1	MitoCarta3.0
152100	CMC1	C-X9-C motif containing 1	MitoCarta3.0
10573	MRPL28	mitochondrial ribosomal protein L28	MitoCarta3.0
10102	TSFM	Ts translation elongation factor, mitochondrial	MitoCarta3.0
2395	FXN	frataxin	MitoCarta3.0
27247	NFU1	NFU1 iron-sulfur cluster scaffold	MitoCarta3.0
51067	YARS2	tyrosyl-tRNA synthetase 2	MitoCarta3.0
219927	MRPL21	mitochondrial ribosomal protein L21	MitoCarta3.0
8659	ALDH4A1	aldehyde dehydrogenase 4 family member A1	MitoCarta3.0
84134	TOMM40L	translocase of outer mitochondrial membrane 40 like	MitoCarta3.0
51103	NDUFAF1	NADH:ubiquinone oxidoreductase complex assembly factor 1	MitoCarta3.0
4716	NDUFB10	NADH:ubiquinone oxidoreductase subunit B10	MitoCarta3.0
36	ACADSB	acyl-CoA dehydrogenase short/branched chain	MitoCarta3.0
64965	MRPS9	mitochondrial ribosomal protein S9	MitoCarta3.0
56997	COQ8A	coenzyme Q8A	MitoCarta3.0
37	ACADVL	acyl-CoA dehydrogenase very long chain	MitoCarta3.0
51117	COQ4	coenzyme Q4	MitoCarta3.0
29088	MRPL15	mitochondrial ribosomal protein L15	MitoCarta3.0
4695	NDUFA2	NADH:ubiquinone oxidoreductase subunit A2	MitoCarta3.0
79590	MRPL24	mitochondrial ribosomal protein L24	MitoCarta3.0
3329	HSPD1	heat shock protein family D (Hsp60) member 1	MitoCarta3.0
4725	NDUFS5	NADH:ubiquinone oxidoreductase subunit S5	MitoCarta3.0
50808	AK3	adenylate kinase 3	MitoCarta3.0
54205	CYCS	cytochrome c, somatic	MitoCarta3.0
4285	MIPEP	mitochondrial intermediate peptidase	MitoCarta3.0
90624	LYRM7	LYR motif containing 7	MitoCarta3.0
1384	CRAT	carnitine O-acetyltransferase	MitoCarta3.0
5096	PCCB	propionyl-CoA carboxylase subunit beta	MitoCarta3.0
51081	MRPS7	mitochondrial ribosomal protein S7	MitoCarta3.0
11222	MRPL3	mitochondrial ribosomal protein L3	MitoCarta3.0
5625	PRODH	proline dehydrogenase 1	MitoCarta3.0
5095	PCCA	propionyl-CoA carboxylase subunit alpha	MitoCarta3.0
56922	MCCC1	methylcrotonoyl-CoA carboxylase 1	MitoCarta3.0
6183	MRPS12	mitochondrial ribosomal protein S12	MitoCarta3.0
81570	CLPB	caseinolytic mitochondrial matrix peptidase chaperone subunit B	MitoCarta3.0
5163	PDK1	pyruvate dehydrogenase kinase 1	MitoCarta3.0
740	MRPL49	mitochondrial ribosomal protein L49	MitoCarta3.0
1347	COX7A2	cytochrome c oxidase subunit 7A2	MitoCarta3.0
84233	TMEM126A	transmembrane protein 126A	MitoCarta3.0
55268	ECHDC2	enoyl-CoA hydratase domain containing 2	MitoCarta3.0
3052	HCCS	holocytochrome c synthase	MitoCarta3.0
11112	HIBADH	3-hydroxyisobutyrate dehydrogenase	MitoCarta3.0
9801	MRPL19	mitochondrial ribosomal protein L19	MitoCarta3.0
64979	MRPL36	mitochondrial ribosomal protein L36	MitoCarta3.0
253512	SLC25A30	solute carrier family 25 member 30	MitoCarta3.0
622	BDH1	3-hydroxybutyrate dehydrogenase 1	MitoCarta3.0
10667	FARS2	phenylalanyl-tRNA synthetase 2, mitochondrial	MitoCarta3.0
22	ABCB7	ATP binding cassette subfamily B member 7	MitoCarta3.0
4580	MTX1	metaxin 1	MitoCarta3.0
4701	NDUFA7	NADH:ubiquinone oxidoreductase subunit A7	MitoCarta3.0
10440	TIMM17A	translocase of inner mitochondrial membrane 17A	MitoCarta3.0
223	ALDH9A1	aldehyde dehydrogenase 9 family member A1	MitoCarta3.0
51023	MRPS18C	mitochondrial ribosomal protein S18C	MitoCarta3.0
92935	MARS2	methionyl-tRNA synthetase 2, mitochondrial	MitoCarta3.0
4329	ALDH6A1	aldehyde dehydrogenase 6 family member A1	MitoCarta3.0
2232	FDXR	ferredoxin reductase	MitoCarta3.0
283459	GATC	glutamyl-tRNA amidotransferase subunit C	MitoCarta3.0
10131	TRAP1	TNF receptor associated protein 1	MitoCarta3.0
27034	ACAD8	acyl-CoA dehydrogenase family member 8	MitoCarta3.0
217	ALDH2	aldehyde dehydrogenase 2 family member	MitoCarta3.0
10105	PPIF	peptidylprolyl isomerase F	MitoCarta3.0
29928	TIMM22	translocase of inner mitochondrial membrane 22	MitoCarta3.0
3712	IVD	isovaleryl-CoA dehydrogenase	MitoCarta3.0
79944	L2HGDH	L-2-hydroxyglutarate dehydrogenase	MitoCarta3.0
23474	ETHE1	ETHE1 persulfide dioxygenase	MitoCarta3.0
55052	MRPL20	mitochondrial ribosomal protein L20	MitoCarta3.0
292	SLC25A5	solute carrier family 25 member 5	MitoCarta3.0
8604	SLC25A12	solute carrier family 25 member 12	MitoCarta3.0
54460	MRPS21	mitochondrial ribosomal protein S21	MitoCarta3.0
56993	TOMM22	translocase of outer mitochondrial membrane 22	MitoCarta3.0
10449	ACAA2	acetyl-CoA acyltransferase 2	MitoCarta3.0
51263	MRPL30	mitochondrial ribosomal protein L30	MitoCarta3.0
9093	DNAJA3	DnaJ heat shock protein family (Hsp40) member A3	MitoCarta3.0
4708	NDUFB2	NADH:ubiquinone oxidoreductase subunit B2	MitoCarta3.0
65993	MRPS34	mitochondrial ribosomal protein S34	MitoCarta3.0
2109	ETFB	electron transfer flavoprotein subunit beta	MitoCarta3.0
246269	AFG1L	AFG1 like ATPase	MitoCarta3.0
93974	ATP5IF1	ATP synthase inhibitory factor subunit 1	MitoCarta3.0
515	ATP5PB	ATP synthase peripheral stalk-membrane subunit b	MitoCarta3.0
1350	COX7C	cytochrome c oxidase subunit 7C	MitoCarta3.0
54927	CHCHD3	coiled-coil-helix-coiled-coil-helix domain containing 3	MitoCarta3.0
80219	COQ10B	coenzyme Q10B	MitoCarta3.0
197322	ACSF3	acyl-CoA synthetase family member 3	MitoCarta3.0
26073	POLDIP2	DNA polymerase delta interacting protein 2	MitoCarta3.0
1468	SLC25A10	solute carrier family 25 member 10	MitoCarta3.0
10165	SLC25A13	solute carrier family 25 member 13	MitoCarta3.0
5165	PDK3	pyruvate dehydrogenase kinase 3	MitoCarta3.0
10873	ME3	malic enzyme 3	MitoCarta3.0
29093	MRPL22	mitochondrial ribosomal protein L22	MitoCarta3.0
3418	IDH2	isocitrate dehydrogenase (NADP(+)) 2	MitoCarta3.0
2639	GCDH	glutaryl-CoA dehydrogenase	MitoCarta3.0
57129	MRPL47	mitochondrial ribosomal protein L47	MitoCarta3.0
27068	PPA2	inorganic pyrophosphatase 2	MitoCarta3.0
65005	MRPL9	mitochondrial ribosomal protein L9	MitoCarta3.0
400916	CHCHD10	coiled-coil-helix-coiled-coil-helix domain containing 10	MitoCarta3.0
10352	WARS2	tryptophanyl tRNA synthetase 2, mitochondrial	MitoCarta3.0
60386	SLC25A19	solute carrier family 25 member 19	MitoCarta3.0
84869	CBR4	carbonyl reductase 4	MitoCarta3.0
91689	SMDT1	single-pass membrane protein with aspartate rich tail 1	MitoCarta3.0
3029	HAGH	hydroxyacylglutathione hydrolase	MitoCarta3.0
1346	COX7A1	cytochrome c oxidase subunit 7A1	MitoCarta3.0
92170	MTG1	mitochondrial ribosome associated GTPase 1	MitoCarta3.0
84987	COX14	cytochrome c oxidase assembly factor COX14	MitoCarta3.0
55967	NDUFA12	NADH:ubiquinone oxidoreductase subunit A12	MitoCarta3.0
51021	MRPS16	mitochondrial ribosomal protein S16	MitoCarta3.0
80298	MTERF2	mitochondrial transcription termination factor 2	MitoCarta3.0
144363	ETFRF1	electron transfer flavoprotein regulatory factor 1	MitoCarta3.0
2820	GPD2	glycerol-3-phosphate dehydrogenase 2	MitoCarta3.0
4705	NDUFA10	NADH:ubiquinone oxidoreductase subunit A10	MitoCarta3.0
4718	NDUFC2	NADH:ubiquinone oxidoreductase subunit C2	MitoCarta3.0
81892	SLIRP	SRA stem-loop interacting RNA binding protein	MitoCarta3.0
4508	MT-ATP6	ATP synthase F0 subunit 6	MitoCarta3.0
4513	MT-CO2	cytochrome c oxidase subunit II	MitoCarta3.0
29090	TIMM21	translocase of inner mitochondrial membrane 21	MitoCarta3.0
4702	NDUFA8	NADH:ubiquinone oxidoreductase subunit A8	MitoCarta3.0
2744	GLS	glutaminase	MitoCarta3.0
54539	NDUFB11	NADH:ubiquinone oxidoreductase subunit B11	MitoCarta3.0
55526	DHTKD1	dehydrogenase E1 and transketolase domain containing 1	MitoCarta3.0
51011	FAHD2A	fumarylacetoacetate hydrolase domain containing 2A	MitoCarta3.0
7923	HSD17B8	hydroxysteroid 17-beta dehydrogenase 8	MitoCarta3.0
84681	HINT2	histidine triad nucleotide binding protein 2	MitoCarta3.0
64969	MRPS5	mitochondrial ribosomal protein S5	MitoCarta3.0
221545	C6orf136	chromosome 6 open reading frame 136	MitoCarta3.0
283377	SPRYD4	SPRY domain containing 4	MitoCarta3.0
387787	LIPT2	lipoyl(octanoyl) transferase 2	MitoCarta3.0
1666	DECR1	2,4-dienoyl-CoA reductase 1	MitoCarta3.0
64949	MRPS26	mitochondrial ribosomal protein S26	MitoCarta3.0
10166	SLC25A15	solute carrier family 25 member 15	MitoCarta3.0
4731	NDUFV3	NADH:ubiquinone oxidoreductase subunit V3	MitoCarta3.0
670	BPHL	biphenyl hydrolase like	MitoCarta3.0
30968	STOML2	stomatin like 2	MitoCarta3.0
22921	MSRB2	methionine sulfoxide reductase B2	MitoCarta3.0
114294	LACTB	lactamase beta	MitoCarta3.0
10452	TOMM40	translocase of outer mitochondrial membrane 40	MitoCarta3.0
114789	SLC25A25	solute carrier family 25 member 25	MitoCarta3.0
203054	ADCK5	aarF domain containing kinase 5	MitoCarta3.0
80724	ACAD10	acyl-CoA dehydrogenase family member 10	MitoCarta3.0
23078	VWA8	von Willebrand factor A domain containing 8	MitoCarta3.0
60492	CCDC90B	coiled-coil domain containing 90B	MitoCarta3.0
55735	DNAJC11	DnaJ heat shock protein family (Hsp40) member C11	MitoCarta3.0
84266	ALKBH7	alkB homolog 7	MitoCarta3.0
2731	GLDC	glycine decarboxylase	MitoCarta3.0
347411	MPC1L	mitochondrial pyruvate carrier 1 like	MitoCarta3.0
117145	THEM4	thioesterase superfamily member 4	MitoCarta3.0
6742	SSBP1	single stranded DNA binding protein 1	MitoCarta3.0
51264	MRPL27	mitochondrial ribosomal protein L27	MitoCarta3.0
150274	HSCB	HscB mitochondrial iron-sulfur cluster cochaperone	MitoCarta3.0
55173	MRPS10	mitochondrial ribosomal protein S10	MitoCarta3.0
205	AK4	adenylate kinase 4	MitoCarta3.0
55210	ATAD3A	ATPase family AAA domain containing 3A	MitoCarta3.0
51253	MRPL37	mitochondrial ribosomal protein L37	MitoCarta3.0
80142	PTGES2	prostaglandin E synthase 2	MitoCarta3.0
10587	TXNRD2	thioredoxin reductase 2	MitoCarta3.0
54988	ACSM5	acyl-CoA synthetase medium chain family member 5	MitoCarta3.0
55847	CISD1	CDGSH iron sulfur domain 1	MitoCarta3.0
64975	MRPL41	mitochondrial ribosomal protein L41	MitoCarta3.0
54996	MTARC2	mitochondrial amidoxime reducing component 2	MitoCarta3.0
64968	MRPS6	mitochondrial ribosomal protein S6	MitoCarta3.0
6150	MRPL23	mitochondrial ribosomal protein L23	MitoCarta3.0
58472	SQOR	sulfide quinone oxidoreductase	MitoCarta3.0
6341	SCO1	synthesis of cytochrome C oxidase 1	MitoCarta3.0
23464	GCAT	glycine C-acetyltransferase	MitoCarta3.0
25902	MTHFD1L	methylenetetrahydrofolate dehydrogenase (NADP+ dependent) 1 like	MitoCarta3.0
10455	ECI2	enoyl-CoA delta isomerase 2	MitoCarta3.0
7381	UQCRB	ubiquinol-cytochrome c reductase binding protein	MitoCarta3.0
10476	ATP5PD	ATP synthase peripheral stalk subunit d	MitoCarta3.0
10632	ATP5MG	ATP synthase membrane subunit g	MitoCarta3.0
131076	CCDC58	coiled-coil domain containing 58	MitoCarta3.0
64087	MCCC2	methylcrotonoyl-CoA carboxylase 2	MitoCarta3.0
84693	MCEE	methylmalonyl-CoA epimerase	MitoCarta3.0
4337	MOCS1	molybdenum cofactor synthesis 1	MitoCarta3.0
4594	MMUT	methylmalonyl-CoA mutase	MitoCarta3.0
92014	SLC25A51	solute carrier family 25 member 51	MitoCarta3.0
6687	SPG7	SPG7 matrix AAA peptidase subunit, paraplegin	MitoCarta3.0
518	ATP5MC3	ATP synthase membrane subunit c locus 3	MitoCarta3.0
84896	ATAD1	ATPase family AAA domain containing 1	MitoCarta3.0
92106	OXNAD1	oxidoreductase NAD binding domain containing 1	MitoCarta3.0
26164	MTG2	mitochondrial ribosome associated GTPase 2	MitoCarta3.0
55157	DARS2	aspartyl-tRNA synthetase 2, mitochondrial	MitoCarta3.0
51258	MRPL51	mitochondrial ribosomal protein L51	MitoCarta3.0
5091	PC	pyruvate carboxylase	MitoCarta3.0
171425	CLYBL	citramalyl-CoA lyase	MitoCarta3.0
50640	PNPLA8	patatin like phospholipase domain containing 8	MitoCarta3.0
23417	MLYCD	malonyl-CoA decarboxylase	MitoCarta3.0
100996939	PYURF	PIGY upstream reading frame	MitoCarta3.0
10245	TIMM17B	translocase of inner mitochondrial membrane 17B	MitoCarta3.0
64983	MRPL32	mitochondrial ribosomal protein L32	MitoCarta3.0
115286	SLC25A26	solute carrier family 25 member 26	MitoCarta3.0
58510	PRODH2	proline dehydrogenase 2	MitoCarta3.0
56954	NIT2	nitrilase family member 2	MitoCarta3.0
112812	FDX2	ferredoxin 2	MitoCarta3.0
4200	ME2	malic enzyme 2	MitoCarta3.0
7284	TUFM	Tu translation elongation factor, mitochondrial	MitoCarta3.0
1339	COX6A2	cytochrome c oxidase subunit 6A2	MitoCarta3.0
10463	SLC30A9	solute carrier family 30 member 9	MitoCarta3.0
132001	TAMM41	TAM41 mitochondrial translocator assembly and maintenance homolog	MitoCarta3.0
100287932	TIMM23	translocase of inner mitochondrial membrane 23	MitoCarta3.0
55005	RMND1	required for meiotic nuclear division 1 homolog	MitoCarta3.0
9927	MFN2	mitofusin 2	MitoCarta3.0
728489	DNLZ	DNL-type zinc finger	MitoCarta3.0
131474	CHCHD4	coiled-coil-helix-coiled-coil-helix domain containing 4	MitoCarta3.0
4706	NDUFAB1	NADH:ubiquinone oxidoreductase subunit AB1	MitoCarta3.0
116285	ACSM1	acyl-CoA synthetase medium chain family member 1	MitoCarta3.0
493753	COA5	cytochrome c oxidase assembly factor 5	MitoCarta3.0
27235	COQ2	coenzyme Q2, polyprenyltransferase	MitoCarta3.0

57107	PDSS2	decaprenyl diphosphate synthase subunit 2	MitoCarta3.0
1962	EHHADH	enoyl-CoA hydratase and 3-hydroxyacyl CoA dehydrogenase	MitoCarta3.0
4722	NDUFS3	NADH:ubiquinone oxidoreductase core subunit S3	MitoCarta3.0
23456	ABCB10	ATP binding cassette subfamily B member 10	MitoCarta3.0
1160	CKMT2	creatine kinase, mitochondrial 2	MitoCarta3.0
64432	MRPS25	mitochondrial ribosomal protein S25	MitoCarta3.0
28976	ACAD9	acyl-CoA dehydrogenase family member 9	MitoCarta3.0
51241	COX16	cytochrome c oxidase assembly factor COX16	MitoCarta3.0
125228	FAM210A	family with sequence similarity 210 member A	MitoCarta3.0
55856	ACOT13	acyl-CoA thioesterase 13	MitoCarta3.0
1374	CPT1A	carnitine palmitoyltransferase 1A	MitoCarta3.0
10901	DHRS4	dehydrogenase/reductase 4	MitoCarta3.0
153768	PRELID2	PRELI domain containing 2	MitoCarta3.0
79587	CARS2	cysteinyl-tRNA synthetase 2, mitochondrial	MitoCarta3.0
2631	NIPSNAP2	nipsnap homolog 2	MitoCarta3.0
4713	NDUFB7	NADH:ubiquinone oxidoreductase subunit B7	MitoCarta3.0
125988	MICOS13	mitochondrial contact site and cristae organizing system subunit 13	MitoCarta3.0
51022	GLRX2	glutaredoxin 2	MitoCarta3.0
10884	MRPS30	mitochondrial ribosomal protein S30	MitoCarta3.0
9997	SCO2	synthesis of cytochrome C oxidase 2	MitoCarta3.0
3028	HSD17B10	hydroxysteroid 17-beta dehydrogenase 10	MitoCarta3.0
79731	NARS2	asparaginyl-tRNA synthetase 2, mitochondrial	MitoCarta3.0
285367	RPUSD3	RNA pseudouridine synthase D3	MitoCarta3.0
6821	SUOX	sulfite oxidase	MitoCarta3.0
1757	SARDH	sarcosine dehydrogenase	MitoCarta3.0
10247	RIDA	reactive intermediate imine deaminase A homolog	MitoCarta3.0
1351	COX8A	cytochrome c oxidase subunit 8A	MitoCarta3.0
6697	SPR	sepiapterin reductase	MitoCarta3.0
4358	MPV17	mitochondrial inner membrane protein MPV17	MitoCarta3.0
284273	ZADH2	zinc binding alcohol dehydrogenase domain containing 2	MitoCarta3.0
29074	MRPL18	mitochondrial ribosomal protein L18	MitoCarta3.0
219	ALDH1B1	aldehyde dehydrogenase 1 family member B1	MitoCarta3.0
118487	CHCHD1	coiled-coil-helix-coiled-coil-helix domain containing 1	MitoCarta3.0
1678	TIMM8A	translocase of inner mitochondrial membrane 8A	MitoCarta3.0
7419	VDAC3	voltage dependent anion channel 3	MitoCarta3.0
29957	SLC25A24	solute carrier family 25 member 24	MitoCarta3.0
51660	MPC1	mitochondrial pyruvate carrier 1	MitoCarta3.0
79751	SLC25A22	solute carrier family 25 member 22	MitoCarta3.0
83447	SLC25A31	solute carrier family 25 member 31	MitoCarta3.0
83733	SLC25A18	solute carrier family 25 member 18	MitoCarta3.0
51095	TRNT1	tRNA nucleotidyl transferase 1	MitoCarta3.0
3416	IDE	insulin degrading enzyme	MitoCarta3.0
373156	GSTK1	glutathione S-transferase kappa 1	MitoCarta3.0
57176	VARS2	valyl-tRNA synthetase 2, mitochondrial	MitoCarta3.0
3336	HSPE1	heat shock protein family E (Hsp10) member 1	MitoCarta3.0
116541	MRPL54	mitochondrial ribosomal protein L54	MitoCarta3.0
64978	MRPL38	mitochondrial ribosomal protein L38	MitoCarta3.0
3396	MRPL58	mitochondrial ribosomal protein L58	MitoCarta3.0
23787	MTCH1	mitochondrial carrier 1	MitoCarta3.0
51499	TRIAP1	TP53 regulated inhibitor of apoptosis 1	MitoCarta3.0
55037	PTCD3	pentatricopeptide repeat domain 3	MitoCarta3.0
1352	COX10	cytochrome c oxidase assembly factor heme A:farnesyltransferase COX10	MitoCarta3.0
8165	AKAP1	A-kinase anchoring protein 1	MitoCarta3.0
55178	MRM3	mitochondrial rRNA methyltransferase 3	MitoCarta3.0
204	AK2	adenylate kinase 2	MitoCarta3.0
54968	TMEM70	transmembrane protein 70	MitoCarta3.0
84273	NOA1	nitric oxide associated 1	MitoCarta3.0
63933	MCUR1	mitochondrial calcium uniporter regulator 1	MitoCarta3.0
51522	TMEM14C	transmembrane protein 14C	MitoCarta3.0
4717	NDUFC1	NADH:ubiquinone oxidoreductase subunit C1	MitoCarta3.0
51318	MRPL35	mitochondrial ribosomal protein L35	MitoCarta3.0
4696	NDUFA3	NADH:ubiquinone oxidoreductase subunit A3	MitoCarta3.0
23590	PDSS1	decaprenyl diphosphate synthase subunit 1	MitoCarta3.0
8034	SLC25A16	solute carrier family 25 member 16	MitoCarta3.0
154791	FMC1	formation of mitochondrial complex V assembly factor 1 homolog	MitoCarta3.0
6296	ACSM3	acyl-CoA synthetase medium chain family member 3	MitoCarta3.0
137682	NDUFAF6	NADH:ubiquinone oxidoreductase complex assembly factor 6	MitoCarta3.0
7350	UCP1	uncoupling protein 1	MitoCarta3.0
94081	SFXN1	sideroflexin 1	MitoCarta3.0
51218	GLRX5	glutaredoxin 5	MitoCarta3.0
138428	PTRH1	peptidyl-tRNA hydrolase 1 homolog	MitoCarta3.0
64963	MRPS11	mitochondrial ribosomal protein S11	MitoCarta3.0
63929	XPNPEP3	X-prolyl aminopeptidase 3	MitoCarta3.0
54704	PDP1	pyruvate dehyrogenase phosphatase catalytic subunit 1	MitoCarta3.0
1632	ECI1	enoyl-CoA delta isomerase 1	MitoCarta3.0
89874	SLC25A21	solute carrier family 25 member 21	MitoCarta3.0
123096	SLC25A29	solute carrier family 25 member 29	MitoCarta3.0
283130	SLC25A45	solute carrier family 25 member 45	MitoCarta3.0
57001	SDHAF3	succinate dehydrogenase complex assembly factor 3	MitoCarta3.0
51012	PRELID3B	PRELI domain containing 3B	MitoCarta3.0
51629	SLC25A39	solute carrier family 25 member 39	MitoCarta3.0
4709	NDUFB3	NADH:ubiquinone oxidoreductase subunit B3	MitoCarta3.0
25996	REXO2	RNA exonuclease 2	MitoCarta3.0
2628	GATM	glycine amidinotransferase	MitoCarta3.0
26515	TIMM10B	translocase of inner mitochondrial membrane 10B	MitoCarta3.0
54938	SARS2	seryl-tRNA synthetase 2, mitochondrial	MitoCarta3.0
55349	CHDH	choline dehydrogenase	MitoCarta3.0
78988	MRPL57	mitochondrial ribosomal protein L57	MitoCarta3.0
414919	C8orf82	chromosome 8 open reading frame 82	MitoCarta3.0
124454	EARS2	glutamyl-tRNA synthetase 2, mitochondrial	MitoCarta3.0
9380	GRHPR	glyoxylate and hydroxypyruvate reductase	MitoCarta3.0
123263	MTFMT	mitochondrial methionyl-tRNA formyltransferase	MitoCarta3.0
79810	PTCD2	pentatricopeptide repeat domain 2	MitoCarta3.0
55862	ECHDC1	ethylmalonyl-CoA decarboxylase 1	MitoCarta3.0
23438	HARS2	histidyl-tRNA synthetase 2, mitochondrial	MitoCarta3.0
4697	NDUFA4	NDUFA4 mitochondrial complex associated	MitoCarta3.0
51079	NDUFA13	NADH:ubiquinone oxidoreductase subunit A13	MitoCarta3.0
128240	NAXE	NAD(P)HX epimerase	MitoCarta3.0
8834	TMEM11	transmembrane protein 11	MitoCarta3.0
83642	SELENOO	selenoprotein O	MitoCarta3.0
162417	NAGS	N-acetylglutamate synthase	MitoCarta3.0
3295	HSD17B4	hydroxysteroid 17-beta dehydrogenase 4	MitoCarta3.0
55471	NDUFAF7	NADH:ubiquinone oxidoreductase complex assembly factor 7	MitoCarta3.0
4942	OAT	ornithine aminotransferase	MitoCarta3.0
54534	MRPL50	mitochondrial ribosomal protein L50	MitoCarta3.0
1349	COX7B	cytochrome c oxidase subunit 7B	MitoCarta3.0
57038	RARS2	arginyl-tRNA synthetase 2, mitochondrial	MitoCarta3.0
339229	OXLD1	oxidoreductase like domain containing 1	MitoCarta3.0
51110	LACTB2	lactamase beta 2	MitoCarta3.0
126328	NDUFA11	NADH:ubiquinone oxidoreductase subunit A11	MitoCarta3.0
54998	AURKAIP1	aurora kinase A interacting protein 1	MitoCarta3.0
79145	CHCHD7	coiled-coil-helix-coiled-coil-helix domain containing 7	MitoCarta3.0
6472	SHMT2	serine hydroxymethyltransferase 2	MitoCarta3.0
10797	MTHFD2	methylenetetrahydrofolate dehydrogenase (NADP+ dependent) 2, methenyltetrahydrofolate cyclohydrolase	MitoCarta3.0
51115	RMDN1	regulator of microtubule dynamics 1	MitoCarta3.0
1373	CPS1	carbamoyl-phosphate synthase 1	MitoCarta3.0
64928	MRPL14	mitochondrial ribosomal protein L14	MitoCarta3.0
51250	MTRES1	mitochondrial transcription rescue factor 1	MitoCarta3.0
112724	RDH13	retinol dehydrogenase 13	MitoCarta3.0
79979	TRMT2B	tRNA methyltransferase 2 homolog B	MitoCarta3.0
64951	MRPS24	mitochondrial ribosomal protein S24	MitoCarta3.0
28958	COA3	cytochrome c oxidase assembly factor 3	MitoCarta3.0
2936	GSR	glutathione-disulfide reductase	MitoCarta3.0
2021	ENDOG	endonuclease G	MitoCarta3.0
587	BCAT2	branched chain amino acid transaminase 2	MitoCarta3.0
79736	TEFM	transcription elongation factor, mitochondrial	MitoCarta3.0
80221	ACSF2	acyl-CoA synthetase family member 2	MitoCarta3.0
55687	TRMU	tRNA mitochondrial 2-thiouridylase	MitoCarta3.0
56945	MRPS22	mitochondrial ribosomal protein S22	MitoCarta3.0
51056	LAP3	leucine aminopeptidase 3	MitoCarta3.0
2746	GLUD1	glutamate dehydrogenase 1	MitoCarta3.0
3155	HMGCL	3-hydroxy-3-methylglutaryl-CoA lyase	MitoCarta3.0
2272	FHIT	fragile histidine triad diadenosine triphosphatase	MitoCarta3.0
51650	MRPS33	mitochondrial ribosomal protein S33	MitoCarta3.0
80224	NUBPL	nucleotide binding protein like	MitoCarta3.0
57226	LYRM2	LYR motif containing 2	MitoCarta3.0
255027	MPV17L	MPV17 mitochondrial inner membrane protein like	MitoCarta3.0
90480	GADD45GIP1	GADD45G interacting protein 1	MitoCarta3.0
7019	TFAM	transcription factor A, mitochondrial	MitoCarta3.0
5832	ALDH18A1	aldehyde dehydrogenase 18 family member A1	MitoCarta3.0
54995	OXSM	3-oxoacyl-ACP synthase, mitochondrial	MitoCarta3.0
56267	KYAT3	kynurenine aminotransferase 3	MitoCarta3.0
80222	TARS2	threonyl-tRNA synthetase 2, mitochondrial	MitoCarta3.0
9238	TBRG4	transforming growth factor beta regulator 4	MitoCarta3.0
157378	TMEM65	transmembrane protein 65	MitoCarta3.0
140823	ROMO1	reactive oxygen species modulator 1	MitoCarta3.0
25824	PRDX5	peroxiredoxin 5	MitoCarta3.0
29103	DNAJC15	DnaJ heat shock protein family (Hsp40) member C15	MitoCarta3.0
79568	MAIP1	matrix AAA peptidase interacting protein 1	MitoCarta3.0
25994	HIGD1A	HIG1 hypoxia inducible domain family member 1A	MitoCarta3.0
283951	C16orf91	chromosome 16 open reading frame 91	MitoCarta3.0
89941	RHOT2	ras homolog family member T2	MitoCarta3.0
9673	SLC25A44	solute carrier family 25 member 44	MitoCarta3.0
51179	HAO2	hydroxyacid oxidase 2	MitoCarta3.0
5018	OXA1L	OXA1L mitochondrial inner membrane protein	MitoCarta3.0
91137	SLC25A46	solute carrier family 25 member 46	MitoCarta3.0
57665	RDH14	retinol dehydrogenase 14	MitoCarta3.0
9556	ATP5MPL	ATP synthase membrane subunit 6.8PL	MitoCarta3.0
11238	CA5B	carbonic anhydrase 5B	MitoCarta3.0
80347	COASY	Coenzyme A synthase	MitoCarta3.0
284106	CISD3	CDGSH iron sulfur domain 3	MitoCarta3.0
2954	GSTZ1	glutathione S-transferase zeta 1	MitoCarta3.0
125965	COX6B2	cytochrome c oxidase subunit 6B2	MitoCarta3.0
51031	GLOD4	glyoxalase domain containing 4	MitoCarta3.0
30	ACAA1	acetyl-CoA acyltransferase 1	MitoCarta3.0
54677	CROT	carnitine O-octanoyltransferase	MitoCarta3.0
64146	PDF	peptide deformylase, mitochondrial	MitoCarta3.0
90639	COX19	cytochrome c oxidase assembly factor COX19	MitoCarta3.0
275	AMT	aminomethyltransferase	MitoCarta3.0
55066	PDPR	pyruvate dehydrogenase phosphatase regulatory subunit	MitoCarta3.0
57486	NLN	neurolysin	MitoCarta3.0
51027	BOLA1	bolA family member 1	MitoCarta3.0
212	ALAS2	5'-aminolevulinate synthase 2	MitoCarta3.0
134266	GRPEL2	GrpE like 2, mitochondrial	MitoCarta3.0
501	ALDH7A1	aldehyde dehydrogenase 7 family member A1	MitoCarta3.0
27440	HDHD5	haloacid dehalogenase like hydrolase domain containing 5	MitoCarta3.0
2671	GFER	growth factor, augmenter of liver regeneration	MitoCarta3.0
83943	IMMP2L	inner mitochondrial membrane peptidase subunit 2	MitoCarta3.0
84701	COX4I2	cytochrome c oxidase subunit 4I2	MitoCarta3.0
54148	MRPL39	mitochondrial ribosomal protein L39	MitoCarta3.0
51166	AADAT	aminoadipate aminotransferase	MitoCarta3.0
65991	FUNDC2	FUN14 domain containing 2	MitoCarta3.0
10434	LYPLA1	lysophospholipase 1	MitoCarta3.0
5161	PDHA2	pyruvate dehydrogenase E1 subunit alpha 2	MitoCarta3.0
54516	MTRF1L	mitochondrial translational release factor 1 like	MitoCarta3.0
84705	GTPBP3	GTP binding protein 3, mitochondrial	MitoCarta3.0
441024	MTHFD2L	methylenetetrahydrofolate dehydrogenase (NADP+ dependent) 2 like	MitoCarta3.0
10730	YME1L1	YME1 like 1 ATPase	MitoCarta3.0
10965	ACOT2	acyl-CoA thioesterase 2	MitoCarta3.0
84334	COA8	cytochrome c oxidase assembly factor 8	MitoCarta3.0
326625	MMAB	metabolism of cobalamin associated B	MitoCarta3.0
5498	PPOX	protoporphyrinogen oxidase	MitoCarta3.0
5825	ABCD3	ATP binding cassette subfamily D member 3	MitoCarta3.0
23410	SIRT3	sirtuin 3	MitoCarta3.0
197257	LDHD	lactate dehydrogenase D	MitoCarta3.0
84311	MRPL45	mitochondrial ribosomal protein L45	MitoCarta3.0
91574	C12orf65	chromosome 12 open reading frame 65	MitoCarta3.0
196294	IMMP1L	inner mitochondrial membrane peptidase subunit 1	MitoCarta3.0
5428	POLG	DNA polymerase gamma, catalytic subunit	MitoCarta3.0
64756	ATPAF1	ATP synthase mitochondrial F1 complex assembly factor 1	MitoCarta3.0
2647	BLOC1S1	biogenesis of lysosomal organelles complex 1 subunit 1	MitoCarta3.0
160428	ALDH1L2	aldehyde dehydrogenase 1 family member L2	MitoCarta3.0
285521	COX18	cytochrome c oxidase assembly factor COX18	MitoCarta3.0
1371	CPOX	coproporphyrinogen oxidase	MitoCarta3.0
23530	NNT	nicotinamide nucleotide transhydrogenase	MitoCarta3.0
284184	NDUFAF8	NADH:ubiquinone oxidoreductase complex assembly factor 8	MitoCarta3.0

91419	ATP23	ATP23 metallopeptidase and ATP synthase assembly factor homolog	MitoCarta3.0
79877	DCAKD	dephospho-CoA kinase domain containing	MitoCarta3.0
9551	ATP5MF	ATP synthase membrane subunit f	MitoCarta3.0
81855	SFXN3	sideroflexin 3	MitoCarta3.0
10157	AASS	aminoadipate-semialdehyde synthase	MitoCarta3.0
56616	DIABLO	diablo IAP-binding mitochondrial protein	MitoCarta3.0
341947	COX8C	cytochrome c oxidase subunit 8C	MitoCarta3.0
5827	PXMP2	peroxisomal membrane protein 2	MitoCarta3.0
64902	AGXT2	alanine--glyoxylate aminotransferase 2	MitoCarta3.0
27165	GLS2	glutaminase 2	MitoCarta3.0
79135	APOO	apolipoprotein O	MitoCarta3.0
84129	ACAD11	acyl-CoA dehydrogenase family member 11	MitoCarta3.0
401207	C5orf63	chromosome 5 open reading frame 63	MitoCarta3.0
112817	HOGA1	4-hydroxy-2-oxoglutarate aldolase 1	MitoCarta3.0
54902	TTC19	tetratricopeptide repeat domain 19	MitoCarta3.0
51335	NGRN	neugrin, neurite outgrowth associated	MitoCarta3.0
285343	TCAIM	T cell activation inhibitor, mitochondrial	MitoCarta3.0
2653	GCSH	glycine cleavage system protein H	MitoCarta3.0
23408	SIRT5	sirtuin 5	MitoCarta3.0
84706	GPT2	glutamic--pyruvic transaminase 2	MitoCarta3.0
100188893	TOMM6	translocase of outer mitochondrial membrane 6	MitoCarta3.0
26355	FAM162A	family with sequence similarity 162 member A	MitoCarta3.0
10058	ABCB6	ATP binding cassette subfamily B member 6 (Langereis blood group)	MitoCarta3.0
28992	MACROD1	mono-ADP ribosylhydrolase 1	MitoCarta3.0
7263	TST	thiosulfate sulfurtransferase	MitoCarta3.0
4512	MT-CO1	cytochrome c oxidase subunit I	MitoCarta3.0
56181	MTFR1L	mitochondrial fission regulator 1 like	MitoCarta3.0
56948	SDR39U1	short chain dehydrogenase/reductase family 39U member 1	MitoCarta3.0
29958	DMGDH	dimethylglycine dehydrogenase	MitoCarta3.0
9016	SLC25A14	solute carrier family 25 member 14	MitoCarta3.0
10650	PRELID3A	PRELI domain containing 3A	MitoCarta3.0
84269	CHCHD5	coiled-coil-helix-coiled-coil-helix domain containing 5	MitoCarta3.0
9868	TOMM70	translocase of outer mitochondrial membrane 70	MitoCarta3.0
4817	NIT1	nitrilase 1	MitoCarta3.0
81889	FAHD1	fumarylacetoacetate hydrolase domain containing 1	MitoCarta3.0
90550	MCU	mitochondrial calcium uniporter	MitoCarta3.0
1593	CYP27A1	cytochrome P450 family 27 subfamily A member 1	MitoCarta3.0
4536	MT-ND2	MTND2	MitoCarta3.0
4538	MT-ND4	NADH dehydrogenase, subunit 4 (complex I)	MitoCarta3.0
4540	MT-ND5	NADH dehydrogenase, subunit 5 (complex I)	MitoCarta3.0
9812	DELE1	DAP3 binding cell death enhancer 1	MitoCarta3.0
79675	FASTKD1	FAST kinase domains 1	MitoCarta3.0
10240	MRPS31	mitochondrial ribosomal protein S31	MitoCarta3.0
10840	ALDH1L1	aldehyde dehydrogenase 1 family member L1	MitoCarta3.0
26024	PTCD1	pentatricopeptide repeat domain 1	MitoCarta3.0
3032	HADHB	hydroxyacyl-CoA dehydrogenase trifunctional multienzyme complex subunit beta	MitoCarta3.0
54802	TRIT1	tRNA isopentenyltransferase 1	MitoCarta3.0
54675	CRLS1	cardiolipin synthase 1	MitoCarta3.0
132158	GLYCTK	glycerate kinase	MitoCarta3.0
81034	SLC25A32	solute carrier family 25 member 32	MitoCarta3.0
57505	AARS2	alanyl-tRNA synthetase 2, mitochondrial	MitoCarta3.0
9941	EXOG	exo/endonuclease G	MitoCarta3.0
51142	CHCHD2	coiled-coil-helix-coiled-coil-helix domain containing 2	MitoCarta3.0
8564	KMO	kynurenine 3-monooxygenase	MitoCarta3.0
94097	SFXN5	sideroflexin 5	MitoCarta3.0
60528	ELAC2	elaC ribonuclease Z 2	MitoCarta3.0
2876	GPX1	glutathione peroxidase 1	MitoCarta3.0
1375	CPT1B	carnitine palmitoyltransferase 1B	MitoCarta3.0
51024	FIS1	fission, mitochondrial 1	MitoCarta3.0
27166	PRELID1	PRELI domain containing 1	MitoCarta3.0
7352	UCP3	uncoupling protein 3	MitoCarta3.0
9481	SLC25A27	solute carrier family 25 member 27	MitoCarta3.0
55972	SLC25A40	solute carrier family 25 member 40	MitoCarta3.0
79085	SLC25A23	solute carrier family 25 member 23	MitoCarta3.0
51409	HEMK1	HemK methyltransferase family member 1	MitoCarta3.0
55186	SLC25A36	solute carrier family 25 member 36	MitoCarta3.0
115209	OMA1	OMA1 zinc metallopeptidase	MitoCarta3.0
137994	LETM2	leucine zipper and EF-hand containing transmembrane protein 2	MitoCarta3.0
115817	DHRS1	dehydrogenase/reductase 1	MitoCarta3.0
2040	STOM	stomatin	MitoCarta3.0
29081	METTL5	methyltransferase like 5	MitoCarta3.0
4482	MSRA	methionine sulfoxide reductase A	MitoCarta3.0
254552	NUDT8	nudix hydrolase 8	MitoCarta3.0
349565	NMNAT3	nicotinamide nucleotide adenylyltransferase 3	MitoCarta3.0
54977	SLC25A38	solute carrier family 25 member 38	MitoCarta3.0
84769	MPV17L2	MPV17 mitochondrial inner membrane protein like 2	MitoCarta3.0
47	ACLY	ATP citrate lyase	MitoCarta3.0
225	ABCD2	ATP binding cassette subfamily D member 2	MitoCarta3.0
80308	FLAD1	flavin adenine dinucleotide synthetase 1	MitoCarta3.0
51601	LIPT1	lipoyltransferase 1	MitoCarta3.0
80017	DGLUCY	D-glutamate cyclase	MitoCarta3.0
5106	PCK2	phosphoenolpyruvate carboxykinase 2, mitochondrial	MitoCarta3.0
133686	NADK2	NAD kinase 2, mitochondrial	MitoCarta3.0
4357	MPST	mercaptopyruvate sulfurtransferase	MitoCarta3.0
25961	NUDT13	nudix hydrolase 13	MitoCarta3.0
31	ACACA	acetyl-CoA carboxylase alpha	MitoCarta3.0
18	ABAT	4-aminobutyrate aminotransferase	MitoCarta3.0
3158	HMGCS2	3-hydroxy-3-methylglutaryl-CoA synthase 2	MitoCarta3.0
7915	ALDH5A1	aldehyde dehydrogenase 5 family member A1	MitoCarta3.0
548596	CKMT1A	creatine kinase, mitochondrial 1A	MitoCarta3.0
23761	PISD	phosphatidylserine decarboxylase	MitoCarta3.0
4710	NDUFB4	NADH:ubiquinone oxidoreductase subunit B4	MitoCarta3.0
10975	UQCR11	ubiquinol-cytochrome c reductase, complex III subunit XI	MitoCarta3.0
84908	FAM136A	family with sequence similarity 136 member A	MitoCarta3.0
401505	TOMM5	translocase of outer mitochondrial membrane 5	MitoCarta3.0
27429	HTRA2	HtrA serine peptidase 2	MitoCarta3.0
4694	NDUFA1	NADH:ubiquinone oxidoreductase subunit A1	MitoCarta3.0
4519	MT-CYB	cytochrome b	MitoCarta3.0
2987	GUK1	guanylate kinase 1	MitoCarta3.0
728294	D2HGDH	D-2-hydroxyglutarate dehydrogenase	MitoCarta3.0
80207	OPA3	outer mitochondrial membrane lipid metabolism regulator OPA3	MitoCarta3.0
57016	AKR1B10	aldo-keto reductase family 1 member B10	MitoCarta3.0
5264	PHYH	phytanoyl-CoA 2-hydroxylase	MitoCarta3.0
91942	NDUFAF2	NADH:ubiquinone oxidoreductase complex assembly factor 2	MitoCarta3.0
51295	ECSIT	ECSIT signaling integrator	MitoCarta3.0
55486	PARL	presenilin associated rhomboid like	MitoCarta3.0
28977	MRPL42	mitochondrial ribosomal protein L42	MitoCarta3.0
3338	DNAJC4	DnaJ heat shock protein family (Hsp40) member C4	MitoCarta3.0
51025	PAM16	presequence translocase associated motor 16	MitoCarta3.0
224	ALDH3A2	aldehyde dehydrogenase 3 family member A2	MitoCarta3.0
23305	ACSL6	acyl-CoA synthetase long chain family member 6	MitoCarta3.0
57380	MRS2	magnesium transporter MRS2	MitoCarta3.0
55347	ABHD10	abhydrolase domain containing 10, depalmitoylase	MitoCarta3.0
135114	HINT3	histidine triad nucleotide binding protein 3	MitoCarta3.0
11264	PXMP4	peroxisomal membrane protein 4	MitoCarta3.0
64172	OSGEPL1	O-sialoglycoprotein endopeptidase like 1	MitoCarta3.0
318	NUDT2	nudix hydrolase 2	MitoCarta3.0
80777	CYB5B	cytochrome b5 type B	MitoCarta3.0
2926	GRSF1	G-rich RNA sequence binding factor 1	MitoCarta3.0
28973	MRPS18B	mitochondrial ribosomal protein S18B	MitoCarta3.0
84277	DNAJC30	DnaJ heat shock protein family (Hsp40) member C30	MitoCarta3.0
847	CAT	catalase	MitoCarta3.0
1716	DGUOK	deoxyguanosine kinase	MitoCarta3.0
23597	ACOT9	acyl-CoA thioesterase 9	MitoCarta3.0
84532	ACSS1	acyl-CoA synthetase short chain family member 1	MitoCarta3.0
55312	RFK	riboflavin kinase	MitoCarta3.0
56910	STARD7	StAR related lipid transfer domain containing 7	MitoCarta3.0
56994	CHPT1	choline phosphotransferase 1	MitoCarta3.0
4509	MT-ATP8	ATP synthase F0 subunit 8	MitoCarta3.0
51300	TIMMDC1	translocase of inner mitochondrial membrane domain containing 1	MitoCarta3.0
219938	SPATA19	spermatogenesis associated 19	MitoCarta3.0
10553	HTATIP2	HIV-1 Tat interactive protein 2	MitoCarta3.0
8574	AKR7A2	aldo-keto reductase family 7 member A2	MitoCarta3.0
1854	DUT	deoxyuridine triphosphatase	MitoCarta3.0
4514	MT-CO3	cytochrome c oxidase III	MitoCarta3.0
25979	DHRS7B	dehydrogenase/reductase 7B	MitoCarta3.0
9617	MTRF1	mitochondrial translation release factor 1	MitoCarta3.0
55260	TMEM143	transmembrane protein 143	MitoCarta3.0
56953	NT5M	5',3'-nucleotidase, mitochondrial	MitoCarta3.0
55288	RHOT1	ras homolog family member T1	MitoCarta3.0
4833	NME4	NME/NM23 nucleoside diphosphate kinase 4	MitoCarta3.0
10588	MTHFS	methenyltetrahydrofolate synthetase	MitoCarta3.0
90580	TIMM29	translocase of inner mitochondrial membrane 29	MitoCarta3.0
55163	PNPO	pyridoxamine 5'-phosphate oxidase	MitoCarta3.0
221154	MICU2	mitochondrial calcium uptake 2	MitoCarta3.0
84105	PCBD2	pterin-4 alpha-carbinolamine dehydratase 2	MitoCarta3.0
55278	QRSL1	glutaminyl-tRNA amidotransferase subunit QRSL1	MitoCarta3.0
57143	ADCK1	aarF domain containing kinase 1	MitoCarta3.0
9588	PRDX6	peroxiredoxin 6	MitoCarta3.0
8508	NIPSNAP1	nipsnap homolog 1	MitoCarta3.0
2617	GARS1	glycyl-tRNA synthetase 1	MitoCarta3.0
123876	ACSM2A	acyl-CoA synthetase medium chain family member 2A	MitoCarta3.0
84275	SLC25A33	solute carrier family 25 member 33	MitoCarta3.0
201229	LYRM9	LYR motif containing 9	MitoCarta3.0
53917	RAB24	RAB24, member RAS oncogene family	MitoCarta3.0
25973	PARS2	prolyl-tRNA synthetase 2, mitochondrial	MitoCarta3.0
122704	MRPL52	mitochondrial ribosomal protein L52	MitoCarta3.0
80324	PUS1	pseudouridine synthase 1	MitoCarta3.0
2806	GOT2	glutamic-oxaloacetic transaminase 2	MitoCarta3.0
55739	NAXD	NAD(P)HX dehydratase	MitoCarta3.0
4537	MT-ND3	NADH dehydrogenase, subunit 3 (complex I)	MitoCarta3.0
84680	ACCS	1-aminocyclopropane-1-carboxylate synthase homolog (inactive)	MitoCarta3.0
51651	PTRH2	peptidyl-tRNA hydrolase 2	MitoCarta3.0
9489	PGS1	phosphatidylglycerophosphate synthase 1	MitoCarta3.0
55074	OXR1	oxidation resistance 1	MitoCarta3.0
127018	LYPLAL1	lysophospholipase like 1	MitoCarta3.0
54943	DNAJC28	DnaJ heat shock protein family (Hsp40) member C28	MitoCarta3.0
2356	FPGS	folylpolyglutamate synthase	MitoCarta3.0
374882	TMEM205	transmembrane protein 205	MitoCarta3.0
401612	SLC25A53	solute carrier family 25 member 53	MitoCarta3.0
150209	AIFM3	apoptosis inducing factor mitochondria associated 3	MitoCarta3.0
4257	MGST1	microsomal glutathione S-transferase 1	MitoCarta3.0
51537	MTFP1	mitochondrial fission process 1	MitoCarta3.0
51287	COA4	cytochrome c oxidase assembly factor 4 homolog	MitoCarta3.0
60491	NIF3L1	NGG1 interacting factor 3 like 1	MitoCarta3.0
126789	PUSL1	pseudouridine synthase like 1	MitoCarta3.0
113675	SDSL	serine dehydratase like	MitoCarta3.0
581	BAX	BCL2 associated X, apoptosis regulator	MitoCarta3.0
57506	MAVS	mitochondrial antiviral signaling protein	MitoCarta3.0
64745	METTL17	methyltransferase like 17	MitoCarta3.0
32	ACACB	acetyl-CoA carboxylase beta	MitoCarta3.0
384	ARG2	arginase 2	MitoCarta3.0
55149	MTPAP	mitochondrial poly(A) polymerase	MitoCarta3.0
79714	CCDC51	coiled-coil domain containing 51	MitoCarta3.0
79783	SUGCT	succinyl-CoA:glutarate-CoA transferase	MitoCarta3.0
10059	DNM1L	dynamin 1 like	MitoCarta3.0
4898	NRDC	nardilysin convertase	MitoCarta3.0
55863	TMEM126B	transmembrane protein 126B	MitoCarta3.0
387338	NSUN4	NOP2/Sun RNA methyltransferase 4	MitoCarta3.0
79611	ACSS3	acyl-CoA synthetase short chain family member 3	MitoCarta3.0
116151	FAM210B	family with sequence similarity 210 member B	MitoCarta3.0
79863	RBFA	ribosome binding factor A	MitoCarta3.0
254042	METAP1D	methionyl aminopeptidase type 1D, mitochondrial	MitoCarta3.0
10768	AHCYL1	adenosylhomocysteinase like 1	MitoCarta3.0
25934	NIPSNAP3A	nipsnap homolog 3A	MitoCarta3.0
64757	MTARC1	mitochondrial amidoxime reducing component 1	MitoCarta3.0
139341	FUNDC1	FUN14 domain containing 1	MitoCarta3.0
2180	ACSL1	acyl-CoA synthetase long chain family member 1	MitoCarta3.0
116228	COX20	cytochrome c oxidase assembly factor COX20	MitoCarta3.0
83693	HSDL1	hydroxysteroid dehydrogenase like 1	MitoCarta3.0
874	CBR3	carbonyl reductase 3	MitoCarta3.0
130916	MTERF4	mitochondrial transcription termination factor 4	MitoCarta3.0
390916	NUDT19	nudix hydrolase 19	MitoCarta3.0
50865	HEBP1	heme binding protein 1	MitoCarta3.0
114971	PTPMT1	protein tyrosine phosphatase mitochondrial 1	MitoCarta3.0
192111	PGAM5	PGAM family member 5, mitochondrial serine/threonine protein phosphatase	MitoCarta3.0

1841	DTYMK	deoxythymidylate kinase	MitoCarta3.0
3735	KARS1	lysyl-tRNA synthetase 1	MitoCarta3.0
6770	STAR	steroidogenic acute regulatory protein	MitoCarta3.0
131118	DNAJC19	DnaJ heat shock protein family (Hsp40) member C19	MitoCarta3.0
5009	OTC	ornithine carbamoyltransferase	MitoCarta3.0
9167	COX7A2L	cytochrome c oxidase subunit 7A2 like	MitoCarta3.0
51001	MTERF3	mitochondrial transcription termination factor 3	MitoCarta3.0
118980	SFXN2	sideroflexin 2	MitoCarta3.0
2194	FASN	fatty acid synthase	MitoCarta3.0
65018	PINK1	PTEN induced kinase 1	MitoCarta3.0
80775	TMEM177	transmembrane protein 177	MitoCarta3.0
7296	TXNRD1	thioredoxin reductase 1	MitoCarta3.0
79896	THNSL1	threonine synthase like 1	MitoCarta3.0
2224	FDPS	farnesyl diphosphate synthase	MitoCarta3.0
3422	IDI1	isopentenyl-diphosphate delta isomerase 1	MitoCarta3.0
7156	TOP3A	DNA topoisomerase III alpha	MitoCarta3.0
94033	FTMT	ferritin mitochondrial	MitoCarta3.0
92483	LDHAL6B	lactate dehydrogenase A like 6B	MitoCarta3.0
9650	MTFR1	mitochondrial fission regulator 1	MitoCarta3.0
23786	BCL2L13	BCL2 like 13	MitoCarta3.0
54931	TRMT10C	tRNA methyltransferase 10C, mitochondrial RNase P subunit	MitoCarta3.0
11332	ACOT7	acyl-CoA thioesterase 7	MitoCarta3.0
51106	TFB1M	transcription factor B1, mitochondrial	MitoCarta3.0
381	ARF5	ADP ribosylation factor 5	MitoCarta3.0
55013	MCUB	mitochondrial calcium uniporter dominant negative subunit beta	MitoCarta3.0
56652	TWNK	twinkle mtDNA helicase	MitoCarta3.0
27249	MMADHC	metabolism of cobalamin associated D	MitoCarta3.0
29920	PYCR2	pyrroline-5-carboxylate reductase 2	MitoCarta3.0
100131187	TSTD1	thiosulfate sulfurtransferase like domain containing 1	MitoCarta3.0
57149	LYRM1	LYR motif containing 1	MitoCarta3.0
114876	OSBPL1A	oxysterol binding protein like 1A	MitoCarta3.0
9401	RECQL4	RecQ like helicase 4	MitoCarta3.0
100131801	PET100	PET100 cytochrome c oxidase chaperone	MitoCarta3.0
54332	GDAP1	ganglioside induced differentiation associated protein 1	MitoCarta3.0
219402	MTIF3	mitochondrial translational initiation factor 3	MitoCarta3.0
7374	UNG	uracil DNA glycosylase	MitoCarta3.0
10249	GLYAT	glycine-N-acyltransferase	MitoCarta3.0
79763	ISOC2	isochorismatase domain containing 2	MitoCarta3.0
516	ATP5MC1	ATP synthase membrane subunit c locus 1	MitoCarta3.0
5860	QDPR	quinoid dihydropteridine reductase	MitoCarta3.0
79934	COQ8B	coenzyme Q8B	MitoCarta3.0
85865	GTPBP10	GTP binding protein 10	MitoCarta3.0
706	TSPO	translocator protein	MitoCarta3.0
2495	FTH1	ferritin heavy chain 1	MitoCarta3.0
55621	TRMT1	tRNA methyltransferase 1	MitoCarta3.0
90956	ADCK2	aarF domain containing kinase 2	MitoCarta3.0
100130890	TSTD3	thiosulfate sulfurtransferase like domain containing 3	MitoCarta3.0
92259	MRPS36	mitochondrial ribosomal protein S36	MitoCarta3.0
57546	PDP2	pyruvate dehyrogenase phosphatase catalytic subunit 2	MitoCarta3.0
55326	AGPAT5	1-acylglycerol-3-phosphate O-acyltransferase 5	MitoCarta3.0
84331	MCRIP2	MAPK regulated corepressor interacting protein 2	MitoCarta3.0
7351	UCP2	uncoupling protein 2	MitoCarta3.0
51312	SLC25A37	solute carrier family 25 member 37	MitoCarta3.0
81894	SLC25A28	solute carrier family 25 member 28	MitoCarta3.0
153328	SLC25A48	solute carrier family 25 member 48	MitoCarta3.0
203427	SLC25A43	solute carrier family 25 member 43	MitoCarta3.0
283600	SLC25A47	solute carrier family 25 member 47	MitoCarta3.0
284427	SLC25A41	solute carrier family 25 member 41	MitoCarta3.0
284723	SLC25A34	solute carrier family 25 member 34	MitoCarta3.0
2053	EPHX2	epoxide hydrolase 2	MitoCarta3.0
11164	NUDT5	nudix hydrolase 5	MitoCarta3.0
10367	MICU1	mitochondrial calcium uptake 1	MitoCarta3.0
6647	SOD1	superoxide dismutase 1	MitoCarta3.0
253190	SERHL2	serine hydrolase like 2	MitoCarta3.0
25915	NDUFAF3	NADH:ubiquinone oxidoreductase complex assembly factor 3	MitoCarta3.0
388962	BOLA3	bolA family member 3	MitoCarta3.0
132299	OCIAD2	OCIA domain containing 2	MitoCarta3.0
55316	RSAD1	radical S-adenosyl methionine domain containing 1	MitoCarta3.0
84303	CHCHD6	coiled-coil-helix-coiled-coil-helix domain containing 6	MitoCarta3.0
22868	FASTKD2	FAST kinase domains 2	MitoCarta3.0
8799	PEX11B	peroxisomal biogenesis factor 11 beta	MitoCarta3.0
25880	TMEM186	transmembrane protein 186	MitoCarta3.0
80119	PIF1	PIF1 5'-to-3' DNA helicase	MitoCarta3.0
2168	FABP1	fatty acid binding protein 1	MitoCarta3.0
215	ABCD1	ATP binding cassette subfamily D member 1	MitoCarta3.0
11212	PLPBP	pyridoxal phosphate binding protein	MitoCarta3.0
1622	DBI	diazepam binding inhibitor, acyl-CoA binding protein	MitoCarta3.0
87178	PNPT1	polyribonucleotide nucleotidyltransferase 1	MitoCarta3.0
116447	TOP1MT	DNA topoisomerase I mitochondrial	MitoCarta3.0
246243	RNASEH1	ribonuclease H1	MitoCarta3.0
10350	ABCA9	ATP binding cassette subfamily A member 9	MitoCarta3.0
6342	SCP2	sterol carrier protein 2	MitoCarta3.0
51559	NT5DC3	5'-nucleotidase domain containing 3	MitoCarta3.0
100272147	CMC4	C-X9-C motif containing 4	MitoCarta3.0
3945	LDHB	lactate dehydrogenase B	MitoCarta3.0
54059	YBEY	ybeY metalloendoribonuclease	MitoCarta3.0
3094	HINT1	histidine triad nucleotide binding protein 1	MitoCarta3.0
79594	MUL1	mitochondrial E3 ubiquitin protein ligase 1	MitoCarta3.0
7084	TK2	thymidine kinase 2	MitoCarta3.0
79814	AGMAT	agmatinase	MitoCarta3.0
129607	CMPK2	cytidine/uridine monophosphate kinase 2	MitoCarta3.0
637	BID	BH3 interacting domain death agonist	MitoCarta3.0
402	ARL2	ADP ribosylation factor like GTPase 2	MitoCarta3.0
55333	SYNJ2BP	synaptojanin 2 binding protein	MitoCarta3.0
56947	MFF	mitochondrial fission factor	MitoCarta3.0
51181	DCXR	dicarbonyl and L-xylulose reductase	MitoCarta3.0
53343	NUDT9	nudix hydrolase 9	MitoCarta3.0
23600	AMACR	alpha-methylacyl-CoA racemase	MitoCarta3.0
55187	VPS13D	vacuolar protein sorting 13 homolog D	MitoCarta3.0
11162	NUDT6	nudix hydrolase 6	MitoCarta3.0
55101	DMAC2	distal membrane arm assembly complex 2	MitoCarta3.0
29960	MRM2	mitochondrial rRNA methyltransferase 2	MitoCarta3.0
578	BAK1	BCL2 antagonist/killer 1	MitoCarta3.0
1723	DHODH	dihydroorotate dehydrogenase (quinone)	MitoCarta3.0
4129	MAOB	monoamine oxidase B	MitoCarta3.0
4259	MGST3	microsomal glutathione S-transferase 3	MitoCarta3.0
51205	ACP6	acid phosphatase 6, lysophosphatidic	MitoCarta3.0
51642	MRPL48	mitochondrial ribosomal protein L48	MitoCarta3.0
189	AGXT	alanine--glyoxylate and serine--pyruvate aminotransferase	MitoCarta3.0
1727	CYB5R3	cytochrome b5 reductase 3	MitoCarta3.0
55794	DDX28	DEAD-box helicase 28	MitoCarta3.0
25953	PNKD	PNKD metallo-beta-lactamase domain containing	MitoCarta3.0
286097	MICU3	mitochondrial calcium uptake family member 3	MitoCarta3.0
664	BNIP3	BCL2 interacting protein 3	MitoCarta3.0
345778	MTX3	metaxin 3	MitoCarta3.0
4595	MUTYH	mutY DNA glycosylase	MitoCarta3.0
8225	GTPBP6	GTP binding protein 6 (putative)	MitoCarta3.0
1583	CYP11A1	cytochrome P450 family 11 subfamily A member 1	MitoCarta3.0
54940	OCIAD1	OCIA domain containing 1	MitoCarta3.0
79828	METTL8	methyltransferase like 8	MitoCarta3.0
389203	SMIM20	small integral membrane protein 20	MitoCarta3.0
84881	RPUSD4	RNA pseudouridine synthase D4	MitoCarta3.0
57150	SMIM8	small integral membrane protein 8	MitoCarta3.0
10201	NME6	NME/NM23 nucleoside diphosphate kinase 6	MitoCarta3.0
23770	FKBP8	FKBP prolyl isomerase 8	MitoCarta3.0
341392	ACSM4	acyl-CoA synthetase medium chain family member 4	MitoCarta3.0
79671	NLRX1	NLR family member X1	MitoCarta3.0
55572	FOXRED1	FAD dependent oxidoreductase domain containing 1	MitoCarta3.0
64216	TFB2M	transcription factor B2, mitochondrial	MitoCarta3.0
152926	PPM1K	protein phosphatase, Mg2+/Mn2+ dependent 1K	MitoCarta3.0
517	ATP5MC2	ATP synthase membrane subunit c locus 2	MitoCarta3.0
4535	MT-ND1	NADH dehydrogenase, subunit 1 (complex I)	MitoCarta3.0
6901	TAZ	tafazzin	MitoCarta3.0
253827	MSRB3	methionine sulfoxide reductase B3	MitoCarta3.0
26027	ACOT11	acyl-CoA thioesterase 11	MitoCarta3.0
55335	NIPSNAP3B	nipsnap homolog 3B	MitoCarta3.0
4913	NTHL1	nth like DNA glycosylase 1	MitoCarta3.0
54708	MARCHF5	membrane associated ring-CH-type finger 5	MitoCarta3.0
119559	SFXN4	sideroflexin 4	MitoCarta3.0
57570	TRMT5	tRNA methyltransferase 5	MitoCarta3.0
293	SLC25A6	solute carrier family 25 member 6	MitoCarta3.0
147407	SLC25A52	solute carrier family 25 member 52	MitoCarta3.0
4968	OGG1	8-oxoguanine DNA glycosylase	MitoCarta3.0
730249	ACOD1	aconitate decarboxylase 1	MitoCarta3.0
81890	QTRT1	queuine tRNA-ribosyltransferase catalytic subunit 1	MitoCarta3.0
84883	AIFM2	apoptosis inducing factor mitochondria associated 2	MitoCarta3.0
7001	PRDX2	peroxiredoxin 2	MitoCarta3.0
26235	FBXL4	F-box and leucine rich repeat protein 4	MitoCarta3.0
57678	GPAM	glycerol-3-phosphate acyltransferase, mitochondrial	MitoCarta3.0
27109	DMAC2L	distal membrane arm assembly complex 2 like	MitoCarta3.0
55177	RMDN3	regulator of microtubule dynamics 3	MitoCarta3.0
84293	PRXL2A	peroxiredoxin like 2A	MitoCarta3.0
84833	ATP5MD	ATP synthase membrane subunit DAPIT	MitoCarta3.0
100303755	PET117	PET117 cytochrome c oxidase chaperone	MitoCarta3.0
4128	MAOA	monoamine oxidase A	MitoCarta3.0
145853	C15orf61	chromosome 15 open reading frame 61	MitoCarta3.0
10721	POLQ	DNA polymerase theta	MitoCarta3.0
83875	BCO2	beta-carotene oxygenase 2	MitoCarta3.0
3429	IFI27	interferon alpha inducible protein 27	MitoCarta3.0
1429	CRYZ	crystallin zeta	MitoCarta3.0
5831	PYCR1	pyrroline-5-carboxylate reductase 1	MitoCarta3.0
55217	TMLHE	trimethyllysine hydroxylase, epsilon	MitoCarta3.0
9804	TOMM20	translocase of outer mitochondrial membrane 20	MitoCarta3.0
79693	YRDC	yrdC N6-threonylcarbamoyltransferase domain containing	MitoCarta3.0
65260	COA7	cytochrome c oxidase assembly factor 7 (putative)	MitoCarta3.0
10202	DHRS2	dehydrogenase/reductase 2	MitoCarta3.0
166785	MMAA	metabolism of cobalamin associated A	MitoCarta3.0
54471	MIEF1	mitochondrial elongation factor 1	MitoCarta3.0
84419	C15orf48	chromosome 15 open reading frame 48	MitoCarta3.0
3980	LIG3	DNA ligase 3	MitoCarta3.0
665	BNIP3L	BCL2 interacting protein 3 like	MitoCarta3.0
60493	FASTKD5	FAST kinase domains 5	MitoCarta3.0
11315	PARK7	Parkinsonism associated deglycase	MitoCarta3.0
55744	COA1	cytochrome c oxidase assembly factor 1 homolog	MitoCarta3.0
170712	COX7B2	cytochrome c oxidase subunit 7B2	MitoCarta3.0
4539	MT-ND4L	NADH dehydrogenase, subunit 4L (complex I)	MitoCarta3.0
23409	SIRT4	sirtuin 4	MitoCarta3.0
84300	UQCC2	ubiquinol-cytochrome c reductase complex assembly factor 2	MitoCarta3.0
84749	USP30	ubiquitin specific peptidase 30	MitoCarta3.0
201626	PDE12	phosphodiesterase 12	MitoCarta3.0
4832	NME3	NME/NM23 nucleoside diphosphate kinase 3	MitoCarta3.0
22907	DHX30	DExH-box helicase 30	MitoCarta3.0
10922	FASTK	Fas activated serine/threonine kinase	MitoCarta3.0
196074	METTL15	methyltransferase like 15	MitoCarta3.0
1591	CYP24A1	cytochrome P450 family 24 subfamily A member 1	MitoCarta3.0
25819	NOCT	nocturnin	MitoCarta3.0
80025	PANK2	pantothenate kinase 2	MitoCarta3.0
763	CA5A	carbonic anhydrase 5A	MitoCarta3.0
64943	NT5DC2	5'-nucleotidase domain containing 2	MitoCarta3.0
83858	ATAD3B	ATPase family AAA domain containing 3B	MitoCarta3.0
26995	TRUB2	TruB pseudouridine synthase family member 2	MitoCarta3.0
5566	PRKACA	protein kinase cAMP-activated catalytic subunit alpha	MitoCarta3.0
118881	COMTD1	catechol-O-methyltransferase domain containing 1	MitoCarta3.0
126129	CPT1C	carnitine palmitoyltransferase 1C	MitoCarta3.0
285315	C3orf33	chromosome 3 open reading frame 33	MitoCarta3.0
84895	MIGA2	mitoguardin 2	MitoCarta3.0
27044	SND1	staphylococcal nuclease and tudor domain containing 1	MitoCarta3.0
2747	GLUD2	glutamate dehydrogenase 2	MitoCarta3.0
84842	HPDL	4-hydroxyphenylpyruvate dioxygenase like	MitoCarta3.0
90806	ANGEL2	angel homolog 2	MitoCarta3.0
65990	ANTKMT	adenine nucleotide translocase lysine methyltransferase	MitoCarta3.0
790955	UQCC3	ubiquinol-cytochrome c reductase complex assembly factor 3	MitoCarta3.0
10549	PRDX4	peroxiredoxin 4	MitoCarta3.0
51654	CDK5RAP1	CDK5 regulatory subunit associated protein 1	MitoCarta3.0
10606	PAICS	phosphoribosylaminoimidazole carboxylase and phosphoribosylaminoimidazolesuccinocarboxamide synthase	MitoCarta3.0
84709	MGARP	mitochondria localized glutamic acid rich protein	MitoCarta3.0

81932	HDHD3	haloacid dehalogenase like hydrolase domain containing 3	MitoCarta3.0
1585	CYP11B2	cytochrome P450 family 11 subfamily B member 2	MitoCarta3.0
56895	AGPAT4	1-acylglycerol-3-phosphate O-acyltransferase 4	MitoCarta3.0
81554	RCC1L	RCC1 like	MitoCarta3.0
222234	FAM185A	family with sequence similarity 185 member A	MitoCarta3.0
5366	PMAIP1	phorbol-12-myristate-13-acetate-induced protein 1	MitoCarta3.0
11022	TDRKH	tudor and KH domain containing	MitoCarta3.0
80024	SLC8B1	solute carrier family 8 member B1	MitoCarta3.0
1594	CYP27B1	cytochrome P450 family 27 subfamily B member 1	MitoCarta3.0
11232	POLG2	DNA polymerase gamma 2, accessory subunit	MitoCarta3.0
284486	THEM5	thioesterase superfamily member 5	MitoCarta3.0
638	BIK	BCL2 interacting killer	MitoCarta3.0
7978	MTERF1	mitochondrial transcription termination factor 1	MitoCarta3.0
113115	MTFR2	mitochondrial fission regulator 2	MitoCarta3.0
2879	GPX4	glutathione peroxidase 4	MitoCarta3.0
387990	TOMM20L	translocase of outer mitochondrial membrane 20 like	MitoCarta3.0
1312	COMT	catechol-O-methyltransferase	MitoCarta3.0
60314	MYG1	MYG1 exonuclease	MitoCarta3.0
64847	SPATA20	spermatogenesis associated 20	MitoCarta3.0
64064	OXCT2	3-oxoacid CoA-transferase 2	MitoCarta3.0
133957	CCDC127	coiled-coil domain containing 127	MitoCarta3.0
1407	CRY1	cryptochrome circadian regulator 1	MitoCarta3.0
348158	ACSM2B	acyl-CoA synthetase medium chain family member 2B	MitoCarta3.0
80179	MYO19	myosin XIX	MitoCarta3.0
92667	MGME1	mitochondrial genome maintenance exonuclease 1	MitoCarta3.0
5414	SEPTIN4	septin 4	MitoCarta3.0
80303	EFHD1	EF-hand domain family member D1	MitoCarta3.0
55969	RAB5IF	RAB5 interacting factor	MitoCarta3.0
328	APEX1	apurinic/apyrimidinic endodeoxyribonuclease 1	MitoCarta3.0
9517	SPTLC2	serine palmitoyltransferase long chain base subunit 2	MitoCarta3.0
90871	DMAC1	distal membrane arm assembly complex 1	MitoCarta3.0
134145	ATPSCKMT	ATP synthase c subunit lysine N-methyltransferase	MitoCarta3.0
129807	NEU4	neuraminidase 4	MitoCarta3.0
842	CASP9	caspase 9	MitoCarta3.0
55750	AGK	acylglycerol kinase	MitoCarta3.0
4170	MCL1	MCL1 apoptosis regulator, BCL2 family member	MitoCarta3.0
54920	DUS2	dihydrouridine synthase 2	MitoCarta3.0
201164	PLD6	phospholipase D family member 6	MitoCarta3.0
10953	TOMM34	translocase of outer mitochondrial membrane 34	MitoCarta3.0
9692	PRORP	protein only RNase P catalytic subunit	MitoCarta3.0
57048	PLSCR3	phospholipid scramblase 3	MitoCarta3.0
1159	CKMT1B	creatine kinase, mitochondrial 1B	MitoCarta3.0
4707	NDUFB1	NADH:ubiquinone oxidoreductase subunit B1	MitoCarta3.0
100526740	ATP5MF-PTCD1	ATP5MF-PTCD1 readthrough	MitoCarta3.0
841	CASP8	caspase 8	MitoCarta3.0
598	BCL2L1	BCL2 like 1	MitoCarta3.0
55218	EXD2	exonuclease 3'-5' domain containing 2	MitoCarta3.0
83787	ARMC10	armadillo repeat containing 10	MitoCarta3.0
125170	MIEF2	mitochondrial elongation factor 2	MitoCarta3.0
54974	THG1L	tRNA-histidine guanylyltransferase 1 like	MitoCarta3.0
64083	GOLPH3	golgi phosphoprotein 3	MitoCarta3.0
9581	PREPL	prolyl endopeptidase like	MitoCarta3.0
79072	FASTKD3	FAST kinase domains 3	MitoCarta3.0
84902	CEP89	centrosomal protein 89	MitoCarta3.0
150763	GPAT2	glycerol-3-phosphate acyltransferase 2, mitochondrial	MitoCarta3.0
4077	NBR1	NBR1 autophagy cargo receptor	MitoCarta3.0
666	BOK	BCL2 family apoptosis regulator BOK	MitoCarta3.0
60681	FKBP10	FKBP prolyl isomerase 10	MitoCarta3.0
133383	SETD9	SET domain containing 9	MitoCarta3.0
1584	CYP11B1	cytochrome P450 family 11 subfamily B member 1	MitoCarta3.0
751071	CSKMT	citrate synthase lysine methyltransferase	MitoCarta3.0
374986	MIGA1	mitoguardin 1	MitoCarta3.0
22934	RPIA	ribose 5-phosphate isomerase A	MitoCarta3.0
27113	BBC3	BCL2 binding component 3	MitoCarta3.0
572	BAD	BCL2 associated agonist of cell death	MitoCarta3.0
596	BCL2	BCL2 apoptosis regulator	MitoCarta3.0
597	BCL2A1	BCL2 related protein A1	MitoCarta3.0
599	BCL2L2	BCL2 like 2	MitoCarta3.0
836	CASP3	caspase 3	MitoCarta3.0
1760	DMPK	DM1 protein kinase	MitoCarta3.0
1763	DNA2	DNA replication helicase/nuclease 2	MitoCarta3.0
4541	MT-ND6	NADH dehydrogenase, subunit 6 (complex I)	MitoCarta3.0
5071	PRKN	parkin RBR E3 ubiquitin protein ligase	MitoCarta3.0
8846	ALKBH1	alkB homolog 1, histone H2A dioxygenase	MitoCarta3.0
9463	PICK1	protein interacting with PRKCA 1	MitoCarta3.0
10017	BCL2L10	BCL2 like 10	MitoCarta3.0
11216	AKAP10	A-kinase anchoring protein 10	MitoCarta3.0
51657	STYXL1	serine/threonine/tyrosine interacting like 1	MitoCarta3.0
54888	NSUN2	NOP2/Sun RNA methyltransferase 2	MitoCarta3.0
55811	ADCY10	adenylate cyclase 10	MitoCarta3.0
56848	SPHK2	sphingosine kinase 2	MitoCarta3.0
63899	NSUN3	NOP2/Sun RNA methyltransferase 3	MitoCarta3.0
140886	PABPC5	poly(A) binding protein cytoplasmic 5	MitoCarta3.0
167681	PRSS35	serine protease 35	MitoCarta3.0
201973	PRIMPOL	primase and DNA directed polymerase	MitoCarta3.0
84947	SERAC1	serine active site containing 1	MitoCarta3.0
55014	STX17	syntaxin 17	MitoCarta3.0
55006	TRMT61B	tRNA methyltransferase 61B	MitoCarta3.0
5138	PDE2A	phosphodiesterase 2A	MitoCarta3.0
55848	PLGRKT	plasminogen receptor with a C-terminal lysine	MitoCarta3.0
254013	ETFBKMT	electron transfer flavoprotein subunit beta lysine methyltransferase	MitoCarta3.0
79680	RTL10	retrotransposon Gag like 10	MitoCarta3.0
10018	BCL2L11	BCL2 like 11	MitoCarta3.0
56907	SPIRE1	spire type actin nucleation factor 1	MitoCarta3.0
205327	C2orf69	chromosome 2 open reading frame 69	MitoCarta3.0
9823	ARMCX2	armadillo repeat containing X-linked 2	MitoCarta3.0
51309	ARMCX1	armadillo repeat containing X-linked 1	MitoCarta3.0
5423	POLB	DNA polymerase beta	MitoCarta3.0
9342	SNAP29	synaptosome associated protein 29	MitoCarta3.0
51566	ARMCX3	armadillo repeat containing X-linked 3	MitoCarta3.0
54470	ARMCX6	armadillo repeat containing X-linked 6	MitoCarta3.0
64863	METTL4	methyltransferase like 4	MitoCarta3.0
80309	SPHKAP	SPHK1 interactor, AKAP domain containing	MitoCarta3.0
339983	NAT8L	N-acetyltransferase 8 like	MitoCarta3.0
401250	MCCD1	mitochondrial coiled-coil domain 1	MitoCarta3.0
101928527	PIGBOS1	PIGB opposite strand 1	MitoCarta3.0
109703458	HTD2	hydroxyacyl-thioester dehydratase type 2	MitoCarta3.0
ENSG00000230623	RP11_469A15.2	RP11_469A15.2	MitoCarta3.0

Table S3. mQTL-harm 412 mQTLs of mitochondria-related genes.

SNP	effect_all	other_alle	effect_all	other_alle	beta.exposu	beta.outcome	eaf.exposure	eaf.outcom	remove	palindrom	ambiguou	id.outcom	chr	pos	se.outcome	samplesiz	pval.outcome
rs100000	C	T	C	T	-1.08973	-0.0100503	0.0427892	NA	FALSE	FALSE	FALSE	ebi-a-GC	4	108826383	0.0877986	14267	0.908865
rs105165	G	A	G	A	-0.480634	0.0392207	0.0761817	NA	FALSE	FALSE	FALSE	ebi-a-GC	4	108821436	0.0486641	14267	0.420273
rs111736	C	T	C	T	-1.12083	-0.105361	0.0242763	NA	FALSE	FALSE	FALSE	ebi-a-GC	4	108700006	0.115197	14267	0.360395
rs116067	C	A	C	A	-0.363177	-0.105361	0.079574	NA	FALSE	FALSE	FALSE	ebi-a-GC	4	108818757	0.0542313	14267	0.0520403
rs129826	G	T	G	T	-0.558553	0.0198026	0.0403249	NA	FALSE	FALSE	FALSE	ebi-a-GC	4	108940029	0.072017	14267	0.783338
rs131031	T	G	T	G	-1.06945	-0.0512933	0.0439315	NA	FALSE	FALSE	FALSE	ebi-a-GC	4	108834529	0.0765653	14267	0.502903
rs131152	G	T	G	T	-0.97543	-0.0512933	0.0361897	NA	FALSE	FALSE	FALSE	ebi-a-GC	4	108841562	0.0744055	14267	0.490587
rs131158	T	G	T	G	-1.13058	-0.0512933	0.0420052	NA	FALSE	FALSE	FALSE	ebi-a-GC	4	108841562	0.0744055	14267	0.490587
rs131200	A	G	A	G	-1.10137	-0.0512933	0.0418276	NA	FALSE	FALSE	FALSE	ebi-a-GC	4	108824239	0.0775548	14267	0.508368
rs131249	C	T	C	T	-0.773393	-0.0202027	0.059527	NA	FALSE	FALSE	FALSE	ebi-a-GC	4	108851173	0.0469684	14267	0.667099
rs131378	G	A	G	A	-1.06945	-0.0512933	0.0439315	NA	FALSE	FALSE	FALSE	ebi-a-GC	4	108844778	0.0766507	14267	0.503379
rs131524	A	G	A	G	-1.06945	-0.0512933	0.0439315	NA	FALSE	FALSE	FALSE	ebi-a-GC	4	108839451	0.0767242	14267	0.503788
rs580429	A	C	A	C	-1.06945	-0.0512933	0.0439315	NA	FALSE	FALSE	FALSE	ebi-a-GC	4	108833945	0.0810814	14267	0.526985
rs676650	T	C	T	C	-0.553779	0.0392207	0.0384039	NA	FALSE	FALSE	FALSE	ebi-a-GC	4	109000449	0.0912989	14267	0.667497
rs684370	T	C	T	C	-0.560333	0.14842	0.0371533	NA	FALSE	FALSE	FALSE	ebi-a-GC	4	109082993	0.0903326	14267	0.100375
rs716010	A	G	A	G	-0.572162	0.131028	0.0388291	NA	FALSE	FALSE	FALSE	ebi-a-GC	4	109094396	0.0875523	14267	0.134505
rs100002	T	C	T	C	-0.365339	0.00995033	0.412095	NA	FALSE	FALSE	FALSE	ebi-a-GC	4	171034742	0.0302116	14267	0.741887
rs100013	C	T	C	T	-0.281648	0.0582689	0.121001	NA	FALSE	FALSE	FALSE	ebi-a-GC	4	171751059	0.0360485	14267	0.106007
rs100072	C	A	C	A	0.292015	0.00995033	0.482007	NA	FALSE	FALSE	FALSE	ebi-a-GC	4	171204323	0.0244605	14267	0.684161
rs100075	T	G	T	G	-0.200436	0.0295588	0.436706	NA	FALSE	FALSE	FALSE	ebi-a-GC	4	170886793	0.0238235	14267	0.214701
rs100081	C	T	C	T	0.260185	0.0198026	0.443139	NA	FALSE	FALSE	FALSE	ebi-a-GC	4	170872226	0.0258722	14267	0.444033
rs100089	T	C	T	C	-0.212967	0.00995033	0.302025	NA	FALSE	FALSE	FALSE	ebi-a-GC	4	171579530	0.0192701	14267	0.605602
rs100095	G	T	G	T	-0.281648	0.0582689	0.121001	NA	FALSE	FALSE	FALSE	ebi-a-GC	4	171758567	0.0357111	14267	0.102748
rs100148	A	C	A	C	-0.321526	0.00995033	0.418279	NA	FALSE	FALSE	FALSE	ebi-a-GC	4	171175473	0.0195759	14267	0.611247
rs100156	C	T	C	T	0.257294	0.00995033	0.437458	NA	FALSE	FALSE	FALSE	ebi-a-GC	4	171140846	0.022246	14267	0.654668
rs100164	T	G	T	G	0.231499	0.00995033	0.303961	NA	FALSE	FALSE	FALSE	ebi-a-GC	4	171033393	0.037189	14267	0.789036
rs100175	C	T	C	T	-0.329401	-0.0202027	0.213874	NA	FALSE	FALSE	FALSE	ebi-a-GC	4	171631091	0.0327631	14267	0.537478
rs100191	C	T	C	T	0.201925	0.0295588	0.461159	NA	FALSE	FALSE	FALSE	ebi-a-GC	4	171252194	0.0290828	14267	0.309454
rs100192	G	A	G	A	-0.233333	0.00995033	0.339182	NA	FALSE	FALSE	FALSE	ebi-a-GC	4	171544440	0.0298322	14267	0.738724
rs100221	A	C	A	C	0.262283	0.00995033	0.446441	NA	FALSE	FALSE	FALSE	ebi-a-GC	4	170872513	0.0159425	14267	0.532536
rs100233	A	G	A	G	0.259962	0.00995033	0.443889	NA	FALSE	FALSE	FALSE	ebi-a-GC	4	171140801	0.0148515	14267	0.502866
rs100243	T	C	T	C	0.246927	0.0198026	0.364578	NA	FALSE	FALSE	FALSE	ebi-a-GC	4	171198948	0.0231297	14267	0.391911
rs100245	C	T	C	T	0.221527	-0.0202027	0.422854	NA	FALSE	FALSE	FALSE	ebi-a-GC	4	171214282	0.0296055	14267	0.494988
rs100258	A	G	A	G	-0.445067	-0.0100503	0.0515337	NA	FALSE	FALSE	FALSE	ebi-a-GC	4	171543005	0.118729	14267	0.93254
rs100261	C	T	C	T	0.205817	-0.0100503	0.387324	NA	FALSE	FALSE	FALSE	ebi-a-GC	4	171184031	0.0462031	14267	0.827799
rs100271	C	T	C	T	-0.182414	0.00995033	0.472916	NA	FALSE	FALSE	FALSE	ebi-a-GC	4	170898746	0.0174195	14267	0.56785
rs100276	A	G	A	G	-0.212967	0.00995033	0.302025	NA	FALSE	FALSE	FALSE	ebi-a-GC	4	171578696	0.0189923	14267	0.60034
rs100285	G	A	G	A	-0.322086	0.00995033	0.416617	NA	FALSE	FALSE	FALSE	ebi-a-GC	4	171175522	0.0191001	14267	0.602396
rs100297	C	T	C	T	-0.250456	0.00995033	0.255767	NA	FALSE	FALSE	FALSE	ebi-a-GC	4	171634817	0.0265179	14267	0.70749
rs100304	A	G	A	G	0.217975	0.0198026	0.265579	NA	FALSE	FALSE	FALSE	ebi-a-GC	4	171167789	0.0288801	14267	0.492913
rs100324	G	A	G	A	0.244512	0.0295588	0.393549	NA	FALSE	FALSE	FALSE	ebi-a-GC	4	171052156	0.0244696	14267	0.227055
rs100326	A	G	A	G	0.280086	0.00995033	0.412474	NA	FALSE	FALSE	FALSE	ebi-a-GC	4	171091454	0.017094	14267	0.560503
rs100331	T	C	T	C	0.259394	-0.0202027	0.266307	NA	FALSE	FALSE	FALSE	ebi-a-GC	4	170900521	0.0235643	14267	0.391255
rs100336	T	C	T	C	0.265626	-0.0100503	0.224079	NA	FALSE	FALSE	FALSE	ebi-a-GC	4	170901019	0.0262479	14267	0.701795
rs100345	A	G	A	G	0.214507	0.00995033	0.364683	NA	FALSE	FALSE	FALSE	ebi-a-GC	4	170881030	0.0271657	14267	0.714154
rs102131	T	C	T	C	-0.217274	0.00995033	0.301305	NA	FALSE	FALSE	FALSE	ebi-a-GC	4	171582128	0.0207591	14267	0.631707
rs104542	A	G	A	G	0.202507	0.00995033	0.261181	NA	FALSE	FALSE	FALSE	ebi-a-GC	4	171225904	0.0228235	14267	0.66286
rs104613	G	A	G	A	0.229246	-0.0202027	0.425716	NA	FALSE	FALSE	FALSE	ebi-a-GC	4	171223920	0.0270827	14267	0.455691
rs105201	A	G	A	G	0.278585	-0.0100503	0.2299	NA	FALSE	FALSE	FALSE	ebi-a-GC	4	170914622	0.0479454	14267	0.833964
rs105201	G	A	G	A	-1.12892	-0.0304592	0.127517	NA	FALSE	FALSE	FALSE	ebi-a-GC	4	171179337	0.0410836	14267	0.458453
rs108663	A	C	A	C	-0.704243	-0.0304592	0.04099	NA	FALSE	FALSE	FALSE	ebi-a-GC	4	171294797	0.0655059	14267	0.641942
rs111227	G	A	G	A	-0.795882	-0.0202027	0.0323591	NA	FALSE	FALSE	FALSE	ebi-a-GC	4	171434389	0.0584931	14267	0.729804
rs111232	T	C	T	C	-0.795882	-0.0202027	0.0323591	NA	FALSE	FALSE	FALSE	ebi-a-GC	4	171432837	0.0580186	14267	0.727683
rs111236	C	T	C	T	-0.663976	-0.0100503	0.0218466	NA	FALSE	FALSE	FALSE	ebi-a-GC	4	171635319	0.0456708	14267	0.825824

rs111262	T	G	T	G	-1.33383	-0.0512933	0.108837	NA	FALSE	FALSE	FALSE	ebi-a-GC	4	171207298	0.0424531	14267	0.226957
rs111275	G	A	G	A	-0.722147	-0.0100503	0.0340348	NA	FALSE	FALSE	FALSE	ebi-a-GC	4	171368910	0.0735191	14267	0.891265
rs111323	T	C	T	C	-1.50083	0.00995033	0.106402	NA	FALSE	FALSE	FALSE	ebi-a-GC	4	171049059	0.0670994	14267	0.882112
rs111328	C	T	C	T	0.268257	0.00995033	0.318287	NA	FALSE	FALSE	FALSE	ebi-a-GC	4	170886340	0.0310165	14267	0.748356
rs111328	G	A	G	A	-0.195996	0.0295588	0.437955	NA	FALSE	FALSE	FALSE	ebi-a-GC	4	170889181	0.023102	14267	0.200725
rs111328	T	C	T	C	-0.328081	-0.0304592	0.460932	NA	FALSE	FALSE	FALSE	ebi-a-GC	4	171075746	0.0281399	14267	0.279066
rs111328	T	G	T	G	0.232177	-0.0100503	0.337754	NA	FALSE	FALSE	FALSE	ebi-a-GC	4	171086368	0.0232474	14267	0.665508
rs111328	G	A	G	A	0.281044	0.00995033	0.441048	NA	FALSE	FALSE	FALSE	ebi-a-GC	4	171093594	0.0168953	14267	0.555902
rs111328	G	A	G	A	-0.329413	-0.00995033	0.485851	NA	FALSE	FALSE	FALSE	ebi-a-GC	4	171093645	0.0154849	14267	0.520494
rs111328	C	T	C	T	-0.329413	-0.00995033	0.485851	NA	FALSE	FALSE	FALSE	ebi-a-GC	4	171095168	0.0154705	14267	0.520107
rs111328	A	G	A	G	0.226799	-0.0202027	0.426437	NA	FALSE	FALSE	FALSE	ebi-a-GC	4	171215956	0.024454	14267	0.408718
rs111328	G	A	G	A	-0.704243	-0.0304592	0.04099	NA	FALSE	FALSE	FALSE	ebi-a-GC	4	171296646	0.0655136	14267	0.641982
rs111328	G	T	G	T	-0.704243	-0.0304592	0.04099	NA	FALSE	FALSE	FALSE	ebi-a-GC	4	171300129	0.0656769	14267	0.642811
rs111328	A	G	A	G	-0.214364	-0.0100503	0.286906	NA	FALSE	FALSE	FALSE	ebi-a-GC	4	171620374	0.0242757	14267	0.678867
rs111328	A	G	A	G	-0.220967	-0.0100503	0.285244	NA	FALSE	FALSE	FALSE	ebi-a-GC	4	171620514	0.0192701	14267	0.601984
rs111337	A	C	A	C	-0.884639	0.00995033	0.0318769	NA	FALSE	FALSE	FALSE	ebi-a-GC	4	171478535	0.0420399	14267	0.812899
rs111340	C	T	C	T	-0.664726	-0.040822	0.0431479	NA	FALSE	FALSE	FALSE	ebi-a-GC	4	171287528	0.0547629	14267	0.456011
rs111346	T	C	T	C	-0.784806	-0.0304592	0.0349913	NA	FALSE	FALSE	FALSE	ebi-a-GC	4	171293592	0.0619386	14267	0.622887
rs111384	G	A	G	A	-1.57906	0.00995033	0.0146176	NA	FALSE	FALSE	FALSE	ebi-a-GC	4	171586878	0.107022	14267	0.925924
rs111390	T	C	T	C	-0.704243	-0.0202027	0.04099	NA	FALSE	FALSE	FALSE	ebi-a-GC	4	171289294	0.054886	14267	0.71281
rs111395	A	G	A	G	-0.856281	0.0198026	0.0311566	NA	FALSE	FALSE	FALSE	ebi-a-GC	4	171543533	0.0665052	14267	0.765886
rs111405	T	G	T	G	-0.887575	0.076961	0.10417	NA	FALSE	FALSE	FALSE	ebi-a-GC	4	170872954	0.0480575	14267	0.10928
rs111421	A	G	A	G	-0.72354	0.00995033	0.0357178	NA	FALSE	FALSE	FALSE	ebi-a-GC	4	171413786	0.0897708	14267	0.911742
rs111454	C	T	C	T	-1.13865	-0.0202027	0.124827	NA	FALSE	FALSE	FALSE	ebi-a-GC	4	171199132	0.0300195	14267	0.500956
rs111460	A	G	A	G	-0.884639	0.00995033	0.0318769	NA	FALSE	FALSE	FALSE	ebi-a-GC	4	171475912	0.0420692	14267	0.813027
rs111466	A	G	A	G	-0.821264	-0.0100503	0.0354724	NA	FALSE	FALSE	FALSE	ebi-a-GC	4	171324371	0.102296	14267	0.921736
rs111471	A	G	A	G	-0.927898	-0.0202027	0.0239732	NA	FALSE	FALSE	FALSE	ebi-a-GC	4	171613791	0.0862779	14267	0.814862
rs111502	T	C	T	C	-1.42875	0.0392207	0.0824532	NA	FALSE	FALSE	FALSE	ebi-a-GC	4	170873288	0.048946	14267	0.422955
rs111508	A	C	A	C	-0.833996	-0.0202027	0.0316388	NA	FALSE	FALSE	FALSE	ebi-a-GC	4	171446410	0.0539731	14267	0.708172
rs111518	A	G	A	G	-0.803628	-0.0100503	0.0336063	NA	FALSE	FALSE	FALSE	ebi-a-GC	4	171352210	0.0537944	14267	0.851795
rs111589	A	G	A	G	-1.01821	0.0487902	0.116756	NA	FALSE	FALSE	FALSE	ebi-a-GC	4	170878705	0.0385568	14267	0.205724
rs111598	A	C	A	C	-0.592757	0.00995033	0.0294839	NA	FALSE	FALSE	FALSE	ebi-a-GC	4	171424380	0.057334	14267	0.862219
rs111598	T	C	T	C	-0.286346	-0.0100503	0.210529	NA	FALSE	FALSE	FALSE	ebi-a-GC	4	171635471	0.0190848	14267	0.598461
rs111632	T	C	T	C	-1.36231	-0.0202027	0.110752	NA	FALSE	FALSE	FALSE	ebi-a-GC	4	171108188	0.0350124	14267	0.563928
rs111640	T	C	T	C	-0.709993	-0.0202027	0.0409876	NA	FALSE	FALSE	FALSE	ebi-a-GC	4	171263981	0.049631	14267	0.683965
rs111688	A	G	A	G	-0.884639	0.00995033	0.0318769	NA	FALSE	FALSE	FALSE	ebi-a-GC	4	171470631	0.0421114	14267	0.813211
rs111729	T	C	T	C	-0.715509	0.00995033	0.0359535	NA	FALSE	FALSE	FALSE	ebi-a-GC	4	171409934	0.09322	14267	0.914995
rs111745	A	G	A	G	-0.702684	-0.0100503	0.0359535	NA	FALSE	FALSE	FALSE	ebi-a-GC	4	171372201	0.0426403	14267	0.813665
rs111752	T	C	T	C	-1.66182	0.00995033	0.0963545	NA	FALSE	FALSE	FALSE	ebi-a-GC	4	170994894	0.0414387	14267	0.810236
rs111810	T	C	T	C	-0.898372	-0.0100503	0.0247252	NA	FALSE	FALSE	FALSE	ebi-a-GC	4	171619526	0.0658285	14267	0.878655
rs111826	C	T	C	T	-0.704243	-0.0202027	0.04099	NA	FALSE	FALSE	FALSE	ebi-a-GC	4	171287273	0.0532792	14267	0.70455
rs111832	C	T	C	T	-0.637865	-0.0100503	0.0223253	NA	FALSE	FALSE	FALSE	ebi-a-GC	4	171645185	0.143778	14267	0.944272
rs111860	A	G	A	G	-0.967072	0.0295588	0.0287623	NA	FALSE	FALSE	FALSE	ebi-a-GC	4	171526399	0.0664677	14267	0.656531
rs111912	T	C	T	C	-1.68603	0.00995033	0.0951603	NA	FALSE	FALSE	FALSE	ebi-a-GC	4	170995313	0.0756586	14267	0.895367
rs112005	T	C	T	C	-0.72354	-0.0100503	0.0357178	NA	FALSE	FALSE	FALSE	ebi-a-GC	4	171389080	0.0976526	14267	0.918027
rs112023	T	C	T	C	-0.833996	-0.0202027	0.0316388	NA	FALSE	FALSE	FALSE	ebi-a-GC	4	171457002	0.0677166	14267	0.765441
rs112024	T	C	T	C	-0.709993	-0.0202027	0.0409876	NA	FALSE	FALSE	FALSE	ebi-a-GC	4	171261918	0.0548954	14267	0.712856
rs112036	A	G	A	G	-1.46612	0.0295588	0.0826972	NA	FALSE	FALSE	FALSE	ebi-a-GC	4	170886594	0.0478622	14267	0.536851
rs112071	C	T	C	T	-1.52219	0.00995033	0.10621	NA	FALSE	FALSE	FALSE	ebi-a-GC	4	171013752	0.0851116	14267	0.906932
rs112079	T	C	T	C	-0.807376	0.0487902	0.0321168	NA	FALSE	FALSE	FALSE	ebi-a-GC	4	171432881	0.0862836	14267	0.571759
rs112079	T	C	T	C	-0.762153	0.00995033	0.0349974	NA	FALSE	FALSE	FALSE	ebi-a-GC	4	171413786	0.0897708	14267	0.911742
rs112086	C	T	C	T	-0.828682	-0.0100503	0.0331276	NA	FALSE	FALSE	FALSE	ebi-a-GC	4	171335929	0.0499421	14267	0.840511
rs112095	A	C	A	C	-0.762779	-0.0100503	0.0349974	NA	FALSE	FALSE	FALSE	ebi-a-GC	4	171366851	0.0833476	14267	0.904021
rs112108	A	G	A	G	-0.819495	-0.0100503	0.035714	NA	FALSE	FALSE	FALSE	ebi-a-GC	4	171323275	0.0993837	14267	0.91945
rs112117	C	T	C	T	-0.795882	-0.0202027	0.0323591	NA	FALSE	FALSE	FALSE	ebi-a-GC	4	171437697	0.0593593	14267	0.733596
rs112167	C	A	C	A	-0.702684	-0.0100503	0.0359535	NA	FALSE	FALSE	FALSE	ebi-a-GC	4	171373842	0.093409	14267	0.914317

Table S4. The all result of two-sample Mendelian randomization analysis for association between mitochondrial gene methylations and systemic lupus erythematosus

id.exposure	id.outcome	outcome	exposure	method	nsnp	b	se	pval	pval1	OR	or_uci95	or_lci95
cg00046913	ebi-a-GCST(	Systemic lup	HAGH	MR Egger	530	0.161136474	0.010390875	5.74E-45	0	1.121015401	1.136847604	1.105007525
cg00046913	ebi-a-GCST(	Systemic lup	HAGH	Weighted me	530	0.12437948	0.004905012	7.40E-142	0	1.092207438	1.09963185	1.085424656
cg00046913	ebi-a-GCST(	Systemic lup	HAGH	Inverse varia	530	0.11510647	0.003664874	1.57E-216	0	1.085424656	1.09085343	1.079975377
cg00046913	ebi-a-GCST(	Systemic lup	HAGH	Simple mode	530	0.146830117	0.016502811	9.09E-18	0	1.109694865	1.134878054	1.084744621
cg00046913	ebi-a-GCST(	Systemic lup	HAGH	Weighted mo	530	0.146830117	0.016962161	5.89E-17	0	1.109694865	1.13553487	1.084064265
cg00086710	ebi-a-GCST(	Systemic lup	PPM1K	MR Egger	13	-0.528326455	0.200449291	0.02316987	0.023	0.669026766	0.9053509	0.483364361
cg00086710	ebi-a-GCST(	Systemic lup	PPM1K	Weighted me	13	0.098434353	0.051184037	0.054461774	0.054	1.07244885	1.150559677	0.998556583
cg00086710	ebi-a-GCST(	Systemic lup	PPM1K	Inverse varia	13	0.195378681	0.033503489	5.49E-09	0	1.147957881	1.200378798	1.096936483
cg00086710	ebi-a-GCST(	Systemic lup	PPM1K	Simple mode	13	0.330619806	0.116613528	0.01503069	0.015	1.25821739	1.458906907	1.075190314
cg00086710	ebi-a-GCST(	Systemic lup	PPM1K	Weighted mo	13	0.330619806	0.064106101	0.000238061	0	1.25821739	1.366252264	1.155101558
cg00121533	ebi-a-GCST(	Systemic lup	CRYZ	MR Egger	269	-0.099640102	0.024271265	5.37E-05	0	0.929790998	0.962734091	0.897627674
cg00121533	ebi-a-GCST(	Systemic lup	CRYZ	Weighted me	269	-0.057089771	0.008688883	5.02E-11	0	0.959770155	0.971589536	0.947853143
cg00121533	ebi-a-GCST(	Systemic lup	CRYZ	Inverse varia	269	-0.060797361	0.005671845	8.28E-27	0	0.956800118	0.964953053	0.949348164
cg00121533	ebi-a-GCST(	Systemic lup	CRYZ	Simple mode	269	-0.066279669	0.021197464	0.00196199	0.002	0.953078953	0.982582947	0.924479992
cg00121533	ebi-a-GCST(	Systemic lup	CRYZ	Weighted mo	269	-0.058728457	0.016003271	0.000292873	0	0.958285901	0.980390956	0.93659083
cg00139037	ebi-a-GCST(	Systemic lup	SLC25A30	MR Egger	78	-0.330014339	0.151808179	0.032826985	0.033	0.781569545	0.976730221	0.617298483
cg00139037	ebi-a-GCST(	Systemic lup	SLC25A30	Weighted me	78	-0.16327231	0.019723995	1.25E-16	0	0.886745225	0.913033	0.86155842
cg00139037	ebi-a-GCST(	Systemic lup	SLC25A30	Inverse varia	78	-0.145743766	0.016264566	3.22E-19	0	0.89840186	0.919912089	0.877351626
cg00139037	ebi-a-GCST(	Systemic lup	SLC25A30	Simple mode	78	-0.1774041	0.045991712	0.00023648	0	0.877351626	0.938097561	0.819668183
cg00139037	ebi-a-GCST(	Systemic lup	SLC25A30	Weighted mo	78	-0.1774041	0.051461561	0.000920578	0.001	0.877351626	0.945607703	0.813114191
cg00225070	ebi-a-GCST(	Systemic lup	MTHFS	MR Egger	12	3.80E-17	1.247298413	1	1	1	3.64696905	0.12035194
cg00225070	ebi-a-GCST(	Systemic lup	MTHFS	Weighted me	12	-0.275792671	0.05673444	1.17E-06	0	0.814755483	0.885964757	0.74760223
cg00225070	ebi-a-GCST(	Systemic lup	MTHFS	Inverse varia	12	-0.27454584	0.044872122	9.45E-10	0	0.815575429	0.871843649	0.76213617
cg00225070	ebi-a-GCST(	Systemic lup	MTHFS	Simple mode	12	-0.275725994	0.085668125	0.008180338	0.008	0.814755483	0.924479992	0.715454127
cg00225070	ebi-a-GCST(	Systemic lup	MTHFS	Weighted mo	12	-0.275725994	0.089733389	0.01060819	0.011	0.814755483	0.929790998	0.711054322
cg00266592	ebi-a-GCST(	Systemic lup	BPHL	MR Egger	38	0.224283656	0.397497843	0.576090482	0.576	1.170566057	1.898014819	0.654435541
cg00266592	ebi-a-GCST(	Systemic lup	BPHL	Weighted me	38	0.057770031	0.015946684	0.000291545	0	1.04194383	1.065572312	1.019346089
cg00266592	ebi-a-GCST(	Systemic lup	BPHL	Inverse varia	38	0.049502693	0.012574126	8.26E-05	0	1.036327492	1.054501216	1.017921908
cg00266592	ebi-a-GCST(	Systemic lup	BPHL	Simple mode	38	0.057719233	0.030768547	0.068573105	0.069	1.04194383	1.087462841	0.997834333
cg00266592	ebi-a-GCST(	Systemic lup	BPHL	Weighted mo	38	0.057719233	0.031635774	0.076160966	0.076	1.04194383	1.088820033	0.997111721
cg00365680	ebi-a-GCST(	Systemic lup	MRPL32	MR Egger	139	-0.04981965	0.016384198	0.002828553	0.003	0.964213778	0.986956963	0.941857519
cg00365680	ebi-a-GCST(	Systemic lup	MRPL32	Weighted me	139	-0.056679809	0.008099097	2.59E-12	0	0.959770155	0.970853654	0.948600847
cg00365680	ebi-a-GCST(	Systemic lup	MRPL32	Inverse varia	139	-0.045826359	0.006143609	8.71E-14	0	0.967168608	0.975996958	0.959028219
cg00365680	ebi-a-GCST(	Systemic lup	MRPL32	Simple mode	139	-0.01882304	0.019543122	0.337154826	0.337	0.98622888	1.014355293	0.959028219
cg00365680	ebi-a-GCST(	Systemic lup	MRPL32	Weighted mo	139	-0.053396432	0.018015619	0.003579361	0.004	0.961993677	0.986956963	0.937344392
cg00452199	ebi-a-GCST(	Systemic lup	ACCS	MR Egger	168	0.019606123	0.017381038	0.260940049	0.261	1.014355293	1.039138394	0.989865623
cg00452199	ebi-a-GCST(	Systemic lup	ACCS	Weighted me	168	0.042338336	0.010453009	5.11E-05	0	1.030689204	1.046141782	1.015782997
cg00452199	ebi-a-GCST(	Systemic lup	ACCS	Inverse varia	168	0.074667968	0.007038713	2.73E-26	0	1.055195654	1.064882852	1.044743821
cg00452199	ebi-a-GCST(	Systemic lup	ACCS	Simple mode	168	0.043773645	0.029582513	0.140833662	0.141	1.032100843	1.075190314	0.989865623
cg00452199	ebi-a-GCST(	Systemic lup	ACCS	Weighted mo	168	0.02967599	0.019161124	0.123332323	0.123	1.021479727	1.049630768	0.994217647
cg00563771	ebi-a-GCST(	Systemic lup	LYRM4	MR Egger	327	-0.048195862	0.010491436	6.23E-06	0	0.965691949	0.980390956	0.951587795
cg00563771	ebi-a-GCST(	Systemic lup	LYRM4	Weighted me	327	-0.045233504	0.005435242	8.63E-17	0	0.96790637	0.975263322	0.96051171
cg00563771	ebi-a-GCST(	Systemic lup	LYRM4	Inverse varia	327	-0.050235864	0.003828557	2.48E-39	0	0.964213778	0.969380765	0.959028219
cg00563771	ebi-a-GCST(	Systemic lup	LYRM4	Simple mode	327	-0.050174435	0.010129414	1.17E-06	0	0.964213778	0.97819563	0.950095094

Table S5. Results of heterogeneity and horizontal pleiotropy tests for positive exposures of TSMR analysis between mQTLs and

id.exposure	exposure	method	Q	Q_df	Q_pval	egger_intercept	se	pval
cg00046913	HAGH	MR Egge	104.9750462	528	1	-0.021400181	0.004520452	2.83E-06
cg00046913	HAGH	Inverse v	127.3865536	529	1		0.004520452	2.83E-06
cg00086710	PPM1K	MR Egge	0.62965618	11	0.999995378	0.17380586	0.047462938	0.003741974
cg00086710	PPM1K	Inverse v	14.03937269	12	0.298201076		0.047462938	0.003741974
cg00121533	CRYZ	MR Egge	198.8831619	267	0.999346392	0.013741142	0.008348551	0.100954483
cg00121533	CRYZ	Inverse v	201.5922518	268	0.99908768		0.008348551	0.100954483
cg00139037	SLC25A3	MR Egge	4.843785668	76	1	0.036391192	0.029807701	0.225911128
cg00139037	SLC25A3	Inverse v	6.33429816	77	1		0.029807701	0.225911128
cg00225070	MTHFS	MR Egge	3.65E-30	10	1	-0.0512933	0.232881464	0.830104062
cg00225070	MTHFS	Inverse v	0.048512252	11	1		0.232881464	0.830104062
cg00266592	BPHL	MR Egge	3.427284457	36	1	-0.146255139	0.332456159	0.66262274
cg00266592	BPHL	Inverse v	3.620816777	37	1		0.332456159	0.66262274
cg00343092	TSPO	MR Egge	69.80595744	55	0.08626526	-0.058560409	0.01889487	0.003055342
cg00343092	TSPO	Inverse v	81.99726155	56	0.013360576		0.01889487	0.003055342
cg00365680	MRPL32	MR Egge	52.99266412	137	1	0.002542194	0.009669404	0.793014022
cg00365680	MRPL32	Inverse v	53.0617864	138	1		0.009669404	0.793014022
cg00452199	ACCS	MR Egge	136.9793555	166	0.951458266	0.021865113	0.006310746	0.00067546
cg00452199	ACCS	Inverse v	148.9838007	167	0.838138933		0.006310746	0.00067546
cg00563771	LYRM4	MR Egge	238.2457538	325	0.999906858	-0.00114958	0.005504412	0.834698541
cg00563771	LYRM4	Inverse v	238.2893708	326	0.999920343		0.005504412	0.834698541
cg00750074	SPG7	MR Egge	629.261154	679	0.914024709	-0.027570366	0.002710693	1.03E-22
cg00750074	SPG7	Inverse v	732.7096973	680	0.078987963		0.002710693	1.03E-22
cg00806900	ACACB	MR Egge	10.93284681	58	1	0.037762527	0.028654354	0.192730999
cg00806900	ACACB	Inverse v	12.66961101	59	1		0.028654354	0.192730999
cg00848461	PRELID1	MR Egge	2.726382695	10	0.987150422	-0.036359458	0.115111302	0.758599942
cg00848461	PRELID1	Inverse v	2.826152439	11	0.992829522		0.115111302	0.758599942
cg00935504	HAGH	MR Egge	1384.133338	1037	2.24E-12	-0.038463201	0.003002213	5.44E-35
cg00935504	HAGH	Inverse v	1603.215481	1038	5.65E-27		0.003002213	5.44E-35
cg01038720	OXR1	MR Egge	36.8348433	90	0.999999881	0.216856457	0.041155524	9.30E-07
cg01038720	OXR1	Inverse v	64.59925272	91	0.983651889		0.041155524	9.30E-07
cg01062247	THEM5	MR Egge	179.0947623	533	1	-0.021044928	0.004236183	9.13E-07
cg01062247	THEM5	Inverse v	203.7747791	534	1		0.004236183	9.13E-07
cg01081346	CPT1B	MR Egge	125.1282415	177	0.998848278	-0.020294015	0.005831226	0.000630832
cg01081346	CPT1B	Inverse v	137.2402535	178	0.989703487		0.005831226	0.000630832
cg01092361	MRPS10	MR Egge	0.817577684	33	1	0.108896598	0.210884322	0.609031783
cg01092361	MRPS10	Inverse v	1.084226733	34	1		0.210884322	0.609031783
cg01227078	SLC25A4	MR Egge	21.05995335	115	1	-0.040974427	0.032139687	0.204919485
cg01227078	SLC25A4	Inverse v	22.68528686	116	1		0.032139687	0.204919485
cg01335087	COMT	MR Egge	1.062734804	9	0.999278802	-0.033923275	0.059054492	0.579742484
cg01335087	COMT	Inverse v	1.392716366	10	0.999232402		0.059054492	0.579742484
cg01416295	MRPL24	MR Egge	2.228088761	31	1	0.005753858	0.037572912	0.879281569
cg01416295	MRPL24	Inverse v	2.251540151	32	1		0.037572912	0.879281569
cg01472317	ACSF3	Inverse v	0.058118561	1	0.80949473	NA	NA	NA
cg01506891	LETM2	MR Egge	0.08242388	16	1	0.090009942	0.817925989	0.913740937
cg01506891	LETM2	Inverse v	0.094534126	17	1		0.817925989	0.913740937
cg01551177	SLC25A2	MR Egge	13.08341296	39	0.999968743	0.01100996	0.021466066	0.610913937
cg01551177	SLC25A2	Inverse v	13.34648006	40	0.999976846		0.021466066	0.610913937
cg01721281	HAGH	MR Egge	310.9896144	410	0.999914844	-0.061971726	0.012109301	4.76E-07
cg01721281	HAGH	Inverse v	337.1804287	411	0.996772911		0.012109301	4.76E-07
cg01724150	NMNAT3	MR Egge	112.0324648	99	0.17481706	0.007453032	0.008530357	0.384392346
cg01724150	NMNAT3	Inverse v	112.8963185	100	0.17824049		0.008530357	0.384392346
cg01812947	NDUFA1(	MR Egge	21.85928141	65	0.9999999	-0.015172954	0.014496862	0.299143479
cg01812947	NDUFA1(	Inverse v	22.9547307	66	0.999999831		0.014496862	0.299143479
cg01878807	DHRS4	MR Egge	17.46391942	166	1	0.030134075	0.010798458	0.005877594
cg01878807	DHRS4	Inverse v	25.25131476	167	1		0.010798458	0.005877594
cg01887435	MTHFD1l	MR Egge	4.222131336	22	0.999986381	-0.024962968	0.069083649	0.721289302
cg01887435	MTHFD1l	Inverse v	4.352700889	23	0.99999234		0.069083649	0.721289302
cg02051290	SERHL2	MR Egge	111.8181778	172	0.999887478	-0.031614288	0.005236145	9.37E-09
cg02051290	SERHL2	Inverse v	148.2720316	173	0.913447835		0.005236145	9.37E-09
cg02119229	BCO2	MR Egge	7.60807952	35	0.999999734	-0.007897946	0.025961974	0.762767717
cg02119229	BCO2	Inverse v	7.700624519	36	0.999999856		0.025961974	0.762767717
cg02187196	PANK2	MR Egge	194.6913148	276	0.999939796	0.00850096	0.003876633	0.029151196
cg02187196	PANK2	Inverse v	199.5000033	277	0.999860051		0.003876633	0.029151196
cg02192472	PDF	MR Egge	126.237946	350	1	-0.023453081	0.007076118	0.001014468
cg02192472	PDF	Inverse v	137.2231884	351	1		0.007076118	0.001014468
cg02193283	ACSF3	MR Egge	158.2455964	293	1	0.024134709	0.009831076	0.014671981
cg02193283	ACSF3	Inverse v	164.2723309	294	1		0.009831076	0.014671981
cg02245810	CPT1B	MR Egge	72.85941875	130	0.999987499	-0.008929592	0.008189549	0.277569596
cg02245810	CPT1B	Inverse v	74.04831319	131	0.999985024		0.008189549	0.277569596
cg02283691	NUDT19	MR Egge	139.4115215	159	0.866356174	-0.130252993	0.009852069	2.15E-27
cg02283691	NUDT19	Inverse v	314.2031063	160	4.13E-12		0.009852069	2.15E-27
cg02333960	COX19	MR Egge	94.11079466	147	0.999783284	0.013013852	0.009851695	0.188562026

Table S6.Summary data-level Mendelian randomization analysis for association between mitochondrial gene methylations and systemic lupus erythematosus

probeID	Genename	ProbeChr	Gene	Probe_bp	topSNP	topSNP_chr	topSNP_bp	A1	A2	Freq	b_GWAS	se_GWAS	p_GWAS	b_eQTL	se_eQTL	p_eQTL	b_SMR	se_SMR	p_SMR	p_HEIDI	nsnp_HEIDI
cg02051290	SERHL2	22	NA	42967981	rs137121	22	43013554	T	C	0.105368	0.0943107	0.0434593	0.02999991	-0.96252	0.0424284	6.19E-114	-0.0979831	0.0453577	0.03075477	0.4819984	20
cg02506360	GPT2	16	NA	46963989	rs11540355	16	46964022	G	A	0.0467197	0.248461	0.0710152	0.00046752	0.683706	0.0715544	1.24E-21	0.363403	0.110612	0.001018438	0.2265865	20
cg02601318	VARS2	6	NA	30882384	rs2596495	6	31323416	C	G	0.0954274	-0.81978	0.0411758	3.38E-88	0.33604	0.0440886	2.50E-14	-2.43953	0.342721	1.09E-12	0.01978458	20
cg04807855	SPR	2	NA	73114341	rs12479094	2	73086770	T	C	0.274354	-0.0676586	0.0335807	0.04392482	0.315341	0.0363028	3.74E-18	-0.214557	0.109317	0.04968095	0.8653448	20
cg04955683	MRPS6	21	NA	35444367	rs743391	21	35434896	C	G	0.505964	-0.0676586	0.0273789	0.01346635	-0.427508	0.031474	5.06E-42	0.158263	0.0650943	0.01504539	0.2051745	20
cg04966294	VARS2	6	NA	30893104	rs28732097	6	31081503	G	C	0.0526839	0.210721	0.068228	0.002011824	0.549174	0.0697908	3.58E-15	0.383705	0.133464	0.004040725	0.1145624	20
cg05045598	ACOT7	1	NA	6418521	rs61763925	1	6418543	G	A	0.0308151	-0.157004	0.0672524	0.01956726	0.824261	0.0843532	1.49E-22	-0.190478	0.0838874	0.02316851	0.8340769	20
cg05305993	COQ7	16	NA	19078614	rs8045418	16	19065708	T	C	0.125249	-0.0861777	0.0429015	0.04456562	0.895188	0.0493028	1.13E-73	-0.0962677	0.048217	0.04587378	0.3547659	20
cg05372963	ABAT	16	NA	8807026	rs59173435	16	8804329	T	C	0.254473	-0.0676586	0.0318663	0.0337365	-0.807934	0.0353078	6.92E-116	0.0837427	0.0396111	0.03450492	0.892993	20
cg05583240	ALDH1L2	12	NA	105477579	rs2440714	12	105544899	A	G	0.149105	0.0943107	0.040009	0.01841154	0.647273	0.0397028	9.41E-60	0.145705	0.0624544	0.01964938	0.2674522	20
cg06004033	NT5DC3	12	NA	104173675	rs954921	12	104173630	C	T	0.378728	-0.0861777	0.028143	0.002197657	0.72097	0.0304748	9.78E-124	-0.11953	0.0393605	0.002391009	0.3068489	20
cg06495347	SUOX	12	NA	56391451	rs1702877	12	56427808	T	C	0.327038	-0.0861777	0.0280921	0.002157248	-0.18407	0.0329835	2.40E-08	0.468179	0.174155	0.007181769	0.278485	5
cg06760507	NDUFS2	1	NA	161168145	rs11576415	1	161182208	G	C	0.10835	-0.10436	0.0505604	0.03901126	-0.889583	0.049943	5.71E-71	0.117313	0.0572164	0.04033073	0.8547147	20
cg08577424	ACAD10	12	NA	112123256	rs642898	12	112141233	G	A	0.259443	0.0943107	0.0350589	0.007143812	-0.939058	0.0321796	3.30E-187	-0.100431	0.0374924	0.007390702	0.8568655	20
cg10457066	NAT8L	4	NA	2065925	rs3135158	4	2066272	C	G	0.100398	-0.10436	0.0447282	0.01963722	-0.456909	0.0552549	1.35E-16	0.228404	0.101715	0.02473425	0.2727804	12
cg11013726	PIF1	15	NA	65116557	rs1684048	15	65132984	A	G	0.441352	0.0725707	0.028636	0.01126886	0.614883	0.0304627	1.33E-90	0.118024	0.0469371	0.01191993	0.02472902	20
cg12259379	MCCD1	6	NA	31496949	rs11482042[illegible]	6	31524511	A	G	0.0159046	0.235722	0.0922765	0.01063335	-2.70437	0.0989193	1.45E-164	-0.0871634	0.0342699	0.01097671	0.08719726	20
cg12453748	PRDX5	11	NA	64085041	rs2083710	11	64168813	C	G	0.082505	0.116534	0.0536755	0.0299254	0.334911	0.05877	1.21E-08	0.347955	0.171505	0.04247539	0.1839163	5
cg12810313	MSRA	8	NA	10192475	rs11249985	8	10171312	A	G	0.317097	0.0833816	0.0331573	0.01191242	-0.256197	0.0345015	1.12E-13	-0.325459	0.136641	0.01722581	0.1002911	20
cg13492245	FPGS	9	NA	130566157	rs73608018	9	130530155	A	G	0.0119284	-0.29267	0.114422	0.01053368	0.826837	0.145944	1.47E-08	-0.353963	0.151835	0.01974103	0.2499831	5
cg14078231	CASP9	1	NA	15850601	rs75461554	1	15810172	T	C	0.187873	-0.076961	0.0357928	0.0315406	0.770108	0.0339891	1.18E-113	-0.0999353	0.0466865	0.03230923	0.667926	20
cg14094639	AURKAIP1	1	NA	1309477	rs12093363	1	1171153	T	C	0.028827	0.198451	0.0898127	0.02713251	-0.66456	0.0985587	1.55E-11	-0.29862	0.142218	0.03575161	0.6532803	20
cg14360014	SARDH	9	NA	136603716	rs2905214	9	136600773	T	C	0.295229	0.105361	0.0328042	0.001319077	0.414871	0.0350527	2.55E-32	0.253961	0.0819305	0.001937106	0.07900229	20
cg16339924	LAP3	4	NA	17578868	rs62296304	4	17578253	G	A	0.348907	0.0618754	0.0291537	0.03380493	-0.287429	0.0308349	1.15E-20	-0.215272	0.104025	0.03850639	0.5424508	20
cg16417840	NAT8L	4	NA	2069925	rs3117813	4	2078853	C	T	0.107356	-0.10436	0.0431492	0.01558117	-0.936236	0.0531416	1.80E-69	0.111468	0.0465202	0.01657005	0.2365586	20
cg17053075	FAM136A	2	NA	70529197	rs11505300[illegible]	2	70402603	G	T	0.0208748	-0.239017	0.102788	0.02005395	0.827275	0.117831	2.21E-12	-0.288921	0.130886	0.02728497	0.4776944	3
cg17290868	NDUFS6	5	NA	1801904	rs56139925	5	1800045	A	G	0.122266	-0.10436	0.045821	0.02275307	0.318006	0.047218	1.64E-11	-0.32817	0.152105	0.03096461	0.02877481	12
cg17967224	BIK	22	NA	43506194	rs5759168	22	43500435	G	A	0.163022	-0.076961	0.0330823	0.02	0.348277	0.0459271	3.37E-14	-0.220976	0.0993577	0.02614512	0.1465301	20
cg18176312	DCAKD	17	NA	43111632	rs9900173	17	43111688	A	G	0.349901	0.0833816	0.0331115	0.01179533	-0.352388	0.0332825	3.40E-26	-0.236619	0.0965843	0.01429088	0.4871502	20
cg19245612	PGAM5	12	NA	133295328	rs12423651	12	133295353	T	C	0.0526839	-0.198851	0.0787791	0.01159765	-1.537	0.0867897	3.54E-70	0.129376	0.0517731	0.0124578	0.5129785	20
cg22450693	SPATA20	17	NA	48624483	rs9890200	17	48624523	C	A	0.349901	0.0833816	0.033068	0.01168503	-0.386873	0.0320579	1.56E-33	-0.215527	0.087321	0.0135788	0.2517788	20
cg22717608	PDHX	11	NA	34937665	rs12385808	11	34961064	A	G	0.117296	-0.157004	0.0436245	0.000319463	0.473669	0.0498743	2.16E-21	-0.331464	0.0984903	0.000764203	0.5858845	20
cg24268004	VARS2	6	NA	30882373	rs2233980	6	31079644	A	G	0.0854871	-0.828552	0.0419672	9.24E-87	0.240782	0.0434822	3.07E-08	-3.44109	0.645398	9.73E-08	0.063799	20
cg24343755	PDSS1	10	NA	26987087	rs1677739	10	26974350	T	C	0.303181	-0.076961	0.0324607	0.01774516	0.45567	0.0358289	4.70E-37	-0.168896	0.0724646	0.01976688	0.9946052	20
cg24721647	ACSL1	4	NA	185726836	rs34277324	4	185788253	T	C	0.182903	0.150823	0.0377232	6.38E-05	0.272505	0.0410994	3.35E-11	0.553469	0.161651	0.000617429	0.1894249	5
cg24757160	NDUFS8	11	NA	67804112	rs3133269	11	67804156	C	T	0.2833	0.0725707	0.0357869	0.04257552	0.779342	0.0340919	1.16E-115	0.0931179	0.0460997	0.0433912	0.1400447	20
cg25698089	MTFMT	15	NA	65321903	rs2946655	15	65321938	G	A	0.0397614	-0.19062	0.0783786	0.01501378	0.484985	0.0745714	7.84E-11	-0.393043	0.172541	0.02272802	0.9315628	3
cg26915924	ABAT	16	NA	8807043	rs59173435	16	8804329	T	C	0.254473	-0.0676586	0.0318663	0.0337365	-0.796652	0.0357473	5.09E-110	0.0849287	0.0401814	0.03454676	0.9671313	20
cg27347728	LAP3	4	NA	17578864	rs7658240	4	17588950	G	A	0.340954	0.0618754	0.0265649	0.01984769	-0.51281	0.0307195	1.47E-62	-0.12066	0.0523044	0.02106213	0.3571362	20

Table S7. Tests of heterogeneity and horizontal pleiotropy of the 23 positive exposures to mitochondria-associated methylation loci (cg) mQTLs

id.exposure	exposure	method	Q	Q_df	Q_pval	egger_intercep	se	pval
cg02051290	SERHL2	IVW	148.272	173.000	0.913	-0.032	0.005	0.000
cg02051290	SERHL2	MR Egger	111.818	172.000	1.000			
cg02601318	VARS2	IVW	1175.252	75.000	0.000	-0.590	0.073	0.000
cg02601318	VARS2	MR Egger	620.616	74.000	0.000			
cg04955683	MRPS6	MR Egger	22.910	53.000	1.000	-0.009	0.015	0.540
cg04955683	MRPS6	IVW	23.291	54.000	1.000			
cg05372963	ABAT	MR Egger	69.228	238.000	1.000	0.029	0.006	0.000
cg05372963	ABAT	IVW	91.759	239.000	1.000			
cg05583240	ALDH1L2	MR Egger	123.316	367.000	1.000	0.020	0.004	0.000
cg05583240	ALDH1L2	IVW	143.838	368.000	1.000			
cg06004033	NT5DC3	MR Egger	289.591	400.000	1.000	-0.044	0.006	0.000
cg06004033	NT5DC3	IVW	343.303	401.000	0.983			
cg08577424	ACAD10	MR Egger	480.689	1189.000	1.000	0.037	0.004	0.000
cg08577424	ACAD10	IVW	575.194	1190.000	1.000			
cg10457066	NAT8L	MR Egger	13.557	53.000	1.000	-0.152	0.028	0.000
cg10457066	NAT8L	IVW	43.492	54.000	0.846			
cg11013726	PIF1	IVW	291.471	186.000	0.000	-0.056	0.009	0.000
cg12810313	MSRA	IVW	88.340	87.000	0.440	0.167	0.022	0.000
cg12810313	MSRA	MR Egger	32.445	86.000	1.000			
cg13492245	FPGS	MR Egger	0.363	4.000	0.985	0.109	1.254	0.935
cg13492245	FPGS	IVW	0.370	5.000	0.996			
cg14078231	CASP9	IVW	970.935	581.000	0.000	-0.020	0.005	0.000
cg14078231	CASP9	MR Egger	941.784	580.000	0.000			
cg14094639	AURKAIP1	MR Egger	6.290	38.000	1.000	0.137	0.027	0.000
cg14094639	AURKAIP1	IVW	32.213	39.000	0.771			
cg16339924	LAP3	IVW	34.787	303.000	1.000	-0.034	0.019	0.073
cg16339924	LAP3	MR Egger	31.561	302.000	1.000			
cg16417840	NAT8L	MR Egger	124.204	316.000	1.000	-0.091	0.009	0.000
cg16417840	NAT8L	IVW	226.218	317.000	1.000			
cg17053075	FAM136A	IVW	0.963	2.000	0.618	0.346	0.409	0.553
cg17053075	FAM136A	MR Egger	0.247	1.000	0.619			
cg18176312	DCAKD	IVW	25.619	85.000	1.000	-0.029	0.022	0.183
cg18176312	DCAKD	MR Egger	23.816	84.000	1.000			
cg22450693	SPATA20	IVW	8.040	27.000	1.000	0.001	0.050	0.988
cg22450693	SPATA20	MR Egger	8.040	26.000	1.000			
cg22717608	PDHX	IVW	33.286	218.000	1.000	-0.011	0.021	0.610
cg22717608	PDHX	MR Egger	33.025	217.000	1.000			
cg24757160	NDUFS8	MR Egger	91.094	177.000	1.000	-0.060	0.008	0.000
cg24757160	NDUFS8	IVW	150.254	178.000	0.936			
cg25698089	MTFMT	IVW	0.031	2.000	0.985	0.340	1.954	0.890
cg25698089	MTFMT	MR Egger	0.001	1.000	0.981			
cg26915924	ABAT	MR Egger	57.696	212.000	1.000	0.029	0.006	0.000
cg26915924	ABAT	IVW	77.349	213.000	1.000			
cg27347728	LAP3	MR Egger	38.822	337.000	1.000	-0.053	0.011	0.000
cg27347728	LAP3	IVW	63.129	338.000	1.000			

Table S8. eQTL-harm 904 eQTLs of mitochondria-related genes.

exposure	Genenam	SNP	effect_all	other_alle	effect_all	other_alle	beta.exposur	beta.outcome	eaf.exposur	eaf.outco	remove	palindrom	ambiguou	id.outcom	chr	pos	se.outcome	samplesiz
ENSG00(	BAD	rs947939	T	C	T	C	0.135846	-0.040822	0.150381	NA	FALSE	FALSE	FALSE	ebi-a-GC:	11	63885287	0.0404337	14267
ENSG00(	LAP3	rs478808	T	C	T	C	-0.0926903	0.00995033	0.419635	NA	FALSE	FALSE	FALSE	ebi-a-GC:	16	28545449	0.0563067	14267
ENSG00(	LAP3	rs685475	G	A	G	A	0.252486	-0.0202027	0.161986	NA	FALSE	FALSE	FALSE	ebi-a-GC:	4	17932555	0.0275352	14267
ENSG00(	LAP3	rs763542	T	C	T	C	0.18982	0.0392207	0.078123	NA	FALSE	FALSE	FALSE	ebi-a-GC:	4	17292326	0.0513341	14267
ENSG00(	LAP3	rs131648	C	T	C	T	-0.0745715	0.0295588	0.283115	NA	FALSE	FALSE	FALSE	ebi-a-GC:	5	131813204	0.0348613	14267
ENSG00(	LAP3	rs109397	C	T	C	T	-0.767993	0.0512933	0.687463	NA	FALSE	FALSE	FALSE	ebi-a-GC:	4	17553407	0.0324774	14267
ENSG00(	LAP3	rs183318	A	G	A	G	0.667866	-0.139262	0.0137021	NA	FALSE	FALSE	FALSE	ebi-a-GC:	4	17629905	0.117731	14267
ENSG00(	ARF5	rs340297	C	T	C	T	-0.104862	0.00995033	0.371437	NA	FALSE	FALSE	FALSE	ebi-a-GC:	7	127230474	0.0313839	14267
ENSG00(	ARF5	rs149110	T	C	T	C	0.18717	-0.0100503	0.0526158	NA	FALSE	FALSE	FALSE	ebi-a-GC:	6	144385777	0.125477	14267
ENSG00(	ARF5	rs149007	T	C	T	C	-0.134987	-0.0304592	0.149708	NA	FALSE	FALSE	FALSE	ebi-a-GC:	7	50370254	0.0430302	14267
ENSG00(	POLDIP2	rs177199	G	A	G	A	-0.149556	-0.105361	0.0677536	NA	FALSE	FALSE	FALSE	ebi-a-GC:	17	26729453	0.0534463	14267
ENSG00(	POLDIP2	rs145923	A	G	A	G	0.539014	0.076961	0.0937308	NA	FALSE	FALSE	FALSE	ebi-a-GC:	17	26706361	0.0503362	14267
ENSG00(	AK2	rs107989	T	C	T	C	-0.188594	0.00995033	0.275752	NA	FALSE	FALSE	FALSE	ebi-a-GC:	1	33511109	0.0447274	14267
ENSG00(	AK2	rs810835	A	G	A	G	0.12385	-0.0295588	0.877992	NA	FALSE	FALSE	FALSE	ebi-a-GC:	1	33179476	0.0400188	14267
ENSG00(	NDUFAB	rs442764	C	T	C	T	-0.121493	-0.0202027	0.0943333	NA	FALSE	FALSE	FALSE	ebi-a-GC:	16	23609390	0.0561914	14267
ENSG00(	PDK4	rs127048	C	T	C	T	-0.251601	0.0487902	0.420253	NA	FALSE	FALSE	FALSE	ebi-a-GC:	7	95175185	0.0283846	14267
ENSG00(	PDK4	rs425377	T	C	T	C	0.180909	-0.0943107	0.0999617	NA	FALSE	FALSE	FALSE	ebi-a-GC:	22	46627603	0.0434593	14267
ENSG00(	PDK4	rs142272	C	T	C	T	-0.245298	0.0582689	0.0296307	NA	FALSE	FALSE	FALSE	ebi-a-GC:	7	95249487	0.0793924	14267
ENSG00(	SLC25A1	rs170873	T	G	T	G	0.0929941	-0.0100503	0.203202	NA	FALSE	FALSE	FALSE	ebi-a-GC:	4	57838583	0.061582	14267
ENSG00(	SLC25A1	rs117085	A	G	A	G	-0.389725	-0.198451	0.0146273	NA	FALSE	FALSE	FALSE	ebi-a-GC:	7	95451189	0.139834	14267
ENSG00(	SLC25A1	rs695765	C	T	C	T	-0.454699	-0.00995033	0.653813	NA	FALSE	FALSE	FALSE	ebi-a-GC:	7	95923037	0.0392524	14267
ENSG00(	MCUB	rs683060	T	C	T	C	0.160229	0.0304592	0.949243	NA	FALSE	FALSE	FALSE	ebi-a-GC:	4	110574049	0.0583781	14267
ENSG00(	MCUB	rs685135	C	G	C	G	0.359202	0.040822	0.667954	NA	FALSE	TRUE	TRUE	ebi-a-GC:	4	110575666	0.0298549	14267
ENSG00(	LIG3	rs229119	C	T	C	T	0.193998	-0.0202027	0.115088	NA	FALSE	FALSE	FALSE	ebi-a-GC:	17	33598061	0.0596965	14267
ENSG00(	LIG3	rs734612	A	G	A	G	0.441507	-0.0304592	0.378481	NA	FALSE	FALSE	FALSE	ebi-a-GC:	17	33138545	0.0350487	14267
ENSG00(	LIG3	rs129417	G	A	G	A	0.1969	-0.00995033	0.735973	NA	FALSE	FALSE	FALSE	ebi-a-GC:	17	33069494	0.0448439	14267
ENSG00(	ACSM3	rs804937	C	T	C	T	-0.0740935	0.00995033	0.444821	NA	FALSE	FALSE	FALSE	ebi-a-GC:	16	20965531	0.0212694	14267
ENSG00(	ACSM3	rs207152	A	G	A	G	-0.332288	-0.0304592	0.13791	NA	FALSE	FALSE	FALSE	ebi-a-GC:	16	20787770	0.041409	14267
ENSG00(	CROT	rs341101	G	A	G	A	-0.719746	-0.0833816	0.0319153	NA	FALSE	FALSE	FALSE	ebi-a-GC:	7	86785942	0.0892005	14267
ENSG00(	CROT	rs113240	A	G	A	G	0.493279	0.0198026	0.0446284	NA	FALSE	FALSE	FALSE	ebi-a-GC:	7	86952492	0.0564633	14267
ENSG00(	PDK2	rs129391	G	C	G	C	0.114182	-0.0100503	0.135432	NA	FALSE	TRUE	TRUE	ebi-a-GC:	17	48244531	0.0314902	14267
ENSG00(	PDK2	rs378592	G	A	G	A	-0.224067	-0.0202027	0.317521	NA	FALSE	FALSE	FALSE	ebi-a-GC:	17	48173720	0.0346764	14267
ENSG00(	SPATA2(	rs557118	T	G	T	G	-0.25749	-0.0202027	0.148828	NA	FALSE	FALSE	FALSE	ebi-a-GC:	17	48242918	0.0574299	14267
ENSG00(	SPATA2(	rs650465	A	G	A	G	-0.49749	-0.0295588	0.586481	NA	FALSE	FALSE	FALSE	ebi-a-GC:	17	48505499	0.0317315	14267
ENSG00(	SPATA2(	rs764877	A	G	A	G	-1.17653	-0.0943107	0.085504	NA	FALSE	FALSE	FALSE	ebi-a-GC:	17	48611993	0.0568532	14267
ENSG00(	SPATA2(	rs111805	A	G	A	G	0.188616	0.0487902	0.087023	NA	FALSE	FALSE	FALSE	ebi-a-GC:	17	48845322	0.0451717	14267
ENSG00(	AGK	rs377908	T	C	T	C	0.20629	-0.0100503	0.42326	NA	FALSE	FALSE	FALSE	ebi-a-GC:	7	141343087	0.02078	14267
ENSG00(	AGK	rs270601	C	T	C	T	-0.0768826	0.0725707	0.669999	NA	FALSE	FALSE	FALSE	ebi-a-GC:	5	131656997	0.0327249	14267
ENSG00(	AGK	rs731539	T	G	T	G	-0.390945	-0.174353	0.0190665	NA	FALSE	FALSE	FALSE	ebi-a-GC:	7	141583683	0.11975	14267
ENSG00(	COX10	rs620538	A	G	A	G	-0.101366	0.0487902	0.362391	NA	FALSE	FALSE	FALSE	ebi-a-GC:	17	13835308	0.0302755	14267
ENSG00(	COX10	rs778775	T	C	T	C	0.61002	-0.105361	0.0347234	NA	FALSE	FALSE	FALSE	ebi-a-GC:	17	13972860	0.0783481	14267
ENSG00(	ELAC2	rs721607	C	T	C	T	-0.556159	-0.0100503	0.187446	NA	FALSE	FALSE	FALSE	ebi-a-GC:	17	12884646	0.0497328	14267
ENSG00(	ELAC2	rs728151	A	G	A	G	-0.214247	0.0676586	0.0257885	NA	FALSE	FALSE	FALSE	ebi-a-GC:	17	12944249	0.0848033	14267
ENSG00(	ELAC2	rs563147	C	T	C	T	0.139755	0.113329	0.0727152	NA	FALSE	FALSE	FALSE	ebi-a-GC:	17	13005683	0.0718772	14267
ENSG00(	AASS	rs612982	A	G	A	G	-0.66794	-0.0202027	0.0906879	NA	FALSE	FALSE	FALSE	ebi-a-GC:	7	121784441	0.061673	14267
ENSG00(	AASS	rs120638	C	A	C	A	0.12343	0.0295588	0.441899	NA	FALSE	FALSE	FALSE	ebi-a-GC:	7	121698827	0.0256383	14267
ENSG00(	MGST1	rs372883	C	T	C	T	-0.124268	-0.0304592	0.494776	NA	FALSE	FALSE	FALSE	ebi-a-GC:	21	30717737	0.0331406	14267
ENSG00(	MGST1	rs414919	A	T	A	T	-0.57357	0.0676586	0.0679486	NA	FALSE	TRUE	TRUE	ebi-a-GC:	12	16502260	0.0535746	14267
ENSG00(	MGST1	rs223963	G	A	G	A	0.103973	0.0100503	0.519173	NA	FALSE	FALSE	FALSE	ebi-a-GC:	14	23589349	0.020316	14267
ENSG00(	MGST1	rs116410	G	C	G	C	0.0820861	-0.235722	0.207945	NA	FALSE	TRUE	TRUE	ebi-a-GC:	16	86014881	0.0404954	14267
ENSG00(	MGST1	rs359798	T	C	T	C	-0.228224	-0.0725707	0.0571059	NA	FALSE	FALSE	FALSE	ebi-a-GC:	12	54685880	0.0540597	14267
ENSG00(	MGST1	rs137549	A	G	A	G	0.0755663	0.0100503	0.565077	NA	FALSE	FALSE	FALSE	ebi-a-GC:	2	182323766	0.0453692	14267
ENSG00(	MGST1	rs100983	A	G	A	G	-0.0925011	0.0202027	0.575007	NA	FALSE	FALSE	FALSE	ebi-a-GC:	8	130613614	0.0250755	14267
ENSG00(	CRY1	rs108617	G	A	G	A	0.184796	-0.00995033	0.553967	NA	FALSE	FALSE	FALSE	ebi-a-GC:	12	107491592	0.0243119	14267

Table S9. The all result of two-sample Mendelian randomization analysis for association between mitochondrial gene expression and systemic lupus erythem

exposure	Genenam	id.exposu	id.outcom	outcome	method	nsnp	b	se	pval	pval1	OR	or_uci95	or_lci95
ENSG00(	BAD	eqtl-a-EN	ebi-a-GC	Systemic	Wald ratio	1	-0.300502039	0.297643655	0.312685352	0.313	0.74	1.327	0.413
ENSG00(	LAP3	eqtl-a-EN	ebi-a-GC	Systemic	Simple m	6	-0.093902794	0.07370361	0.258647699	0.259	0.91	1.052	0.788
ENSG00(	LAP3	eqtl-a-EN	ebi-a-GC	Systemic	Weighted	6	-0.069040626	0.039760728	0.082492694	0.082	0.933	1.009	0.863
ENSG00(	LAP3	eqtl-a-EN	ebi-a-GC	Systemic	Inverse va	6	-0.071890258	0.037893982	0.057809222	0.058	0.931	1.002	0.864
ENSG00(	LAP3	eqtl-a-EN	ebi-a-GC	Systemic	Weighted	6	-0.068475755	0.040847243	0.154509614	0.155	0.934	1.012	0.862
ENSG00(	LAP3	eqtl-a-EN	ebi-a-GC	Systemic	MR Egge	6	-0.064197338	0.060090749	0.345553771	0.346	0.938	1.055	0.834
ENSG00(	ARF5	eqtl-a-EN	ebi-a-GC	Systemic	Weighted	3	-0.05795338	0.232069187	0.802800349	0.803	0.944	1.487	0.599
ENSG00(	ARF5	eqtl-a-EN	ebi-a-GC	Systemic	Simple m	3	-0.074928484	0.283104706	0.816045854	0.816	0.928	1.616	0.533
ENSG00(	ARF5	eqtl-a-EN	ebi-a-GC	Systemic	Inverse va	3	0.044844103	0.20747918	0.8288803	0.829	1.046	1.571	0.696
ENSG00(	ARF5	eqtl-a-EN	ebi-a-GC	Systemic	MR Egge	3	0.578330356	1.254972204	0.725091943	0.725	1.783	20.865	0.152
ENSG00(	ARF5	eqtl-a-EN	ebi-a-GC	Systemic	Weighted	3	-0.089818134	0.280758973	0.779362904	0.779	0.914	1.585	0.527
ENSG00(	POLDIP2	eqtl-a-EN	ebi-a-GC	Systemic	Inverse va	2	0.178686362	0.090351749	0.047965425	0.048	1.196	1.427	1.002
ENSG00(	AK2	eqtl-a-EN	ebi-a-GC	Systemic	Inverse va	2	-0.11784708	0.191190945	0.537640972	0.538	0.889	1.293	0.611
ENSG00(	NDUFAB	eqtl-a-EN	ebi-a-GC	Systemic	Wald ratio	1	0.166286947	0.462507305	0.719195898	0.719	1.181	2.924	0.477
ENSG00(	PDK4	eqtl-a-EN	ebi-a-GC	Systemic	Weighted	3	-0.206805294	0.103999674	0.046754356	0.047	0.813	0.997	0.663
ENSG00(	PDK4	eqtl-a-EN	ebi-a-GC	Systemic	Simple m	3	-0.215489247	0.166426402	0.324717703	0.325	0.806	1.117	0.582
ENSG00(	PDK4	eqtl-a-EN	ebi-a-GC	Systemic	Weighted	3	-0.197648904	0.113134626	0.22274557	0.223	0.821	1.024	0.657
ENSG00(	PDK4	eqtl-a-EN	ebi-a-GC	Systemic	MR Egge	3	0.63772192	0.728015422	0.542027917	0.542	1.892	7.882	0.454
ENSG00(	PDK4	eqtl-a-EN	ebi-a-GC	Systemic	Inverse va	3	-0.251671025	0.097383917	0.009757174	0.01	0.778	0.941	0.642
ENSG00(	SLC25A1	eqtl-a-EN	ebi-a-GC	Systemic	Weighted	3	0.039919821	0.085157823	0.63923079	0.639	1.041	1.23	0.881
ENSG00(	SLC25A1	eqtl-a-EN	ebi-a-GC	Systemic	Weighted	3	0.020070815	0.094295277	0.851168118	0.851	1.02	1.227	0.848
ENSG00(	SLC25A1	eqtl-a-EN	ebi-a-GC	Systemic	MR Egge	3	0.070237241	0.269066245	0.837443844	0.837	1.073	1.818	0.633
ENSG00(	SLC25A1	eqtl-a-EN	ebi-a-GC	Systemic	Inverse va	3	0.046072937	0.083264952	0.580037949	0.58	1.047	1.233	0.889
ENSG00(	SLC25A1	eqtl-a-EN	ebi-a-GC	Systemic	Simple m	3	-0.042463154	0.197774154	0.849900425	0.85	0.958	1.412	0.65
ENSG00(	MCUB	eqtl-a-EN	ebi-a-GC	Systemic	Wald ratio	1	0.190097922	0.364341661	0.601839321	0.602	1.209	2.47	0.592
ENSG00(	LIG3	eqtl-a-EN	ebi-a-GC	Systemic	Inverse va	3	-0.069071003	0.072831231	0.342940787	0.343	0.933	1.076	0.809
ENSG00(	LIG3	eqtl-a-EN	ebi-a-GC	Systemic	MR Egge	3	-0.068204912	0.204099324	0.794685061	0.795	0.934	1.394	0.626
ENSG00(	LIG3	eqtl-a-EN	ebi-a-GC	Systemic	Simple m	3	-0.063060806	0.104036507	0.606053466	0.606	0.939	1.151	0.766
ENSG00(	LIG3	eqtl-a-EN	ebi-a-GC	Systemic	Weighted	3	-0.068083968	0.074302886	0.359507919	0.36	0.934	1.081	0.808
ENSG00(	LIG3	eqtl-a-EN	ebi-a-GC	Systemic	Weighted	3	-0.0680994	0.082967441	0.498028953	0.498	0.934	1.099	0.794
ENSG00(	ACSM3	eqtl-a-EN	ebi-a-GC	Systemic	Inverse va	2	0.055834218	0.114311135	0.625237672	0.625	1.057	1.323	0.845
ENSG00(	CROT	eqtl-a-EN	ebi-a-GC	Systemic	Inverse va	2	0.074994874	0.084087379	0.37246349	0.372	1.078	1.271	0.914
ENSG00(	PDK2	eqtl-a-EN	ebi-a-GC	Systemic	Wald ratio	1	0.090163656	0.154759068	0.560158152	0.56	1.094	1.482	0.808
ENSG00(	SPATA20	eqtl-a-EN	ebi-a-GC	Systemic	Inverse va	4	0.077320295	0.037487397	0.039153715	0.039	1.08	1.163	1.004
ENSG00(	SPATA20	eqtl-a-EN	ebi-a-GC	Systemic	Weighted	4	0.077719938	0.042778483	0.166852938	0.167	1.081	1.175	0.994
ENSG00(	SPATA20	eqtl-a-EN	ebi-a-GC	Systemic	Weighted	4	0.079216832	0.038012127	0.037161222	0.037	1.082	1.166	1.005
ENSG00(	SPATA20	eqtl-a-EN	ebi-a-GC	Systemic	MR Egge	4	0.05586375	0.06954211	0.50609387	0.506	1.057	1.212	0.923
ENSG00(	SPATA20	eqtl-a-EN	ebi-a-GC	Systemic	Simple m	4	0.077206845	0.058953123	0.281586103	0.282	1.08	1.213	0.962
ENSG00(	AGK	eqtl-a-EN	ebi-a-GC	Systemic	Weighted	3	-0.027215686	0.099293966	0.809728325	0.81	0.973	1.182	0.801
ENSG00(	AGK	eqtl-a-EN	ebi-a-GC	Systemic	MR Egge	3	0.582528552	0.262579541	0.269598475	0.27	1.791	2.996	1.07
ENSG00(	AGK	eqtl-a-EN	ebi-a-GC	Systemic	Weighted	3	-0.023657624	0.101979495	0.816550342	0.817	0.977	1.193	0.8
ENSG00(	AGK	eqtl-a-EN	ebi-a-GC	Systemic	Simple m	3	0.10322728	0.186146607	0.634938306	0.635	1.109	1.597	0.77
ENSG00(	AGK	eqtl-a-EN	ebi-a-GC	Systemic	Inverse va	3	-0.045829801	0.175041916	0.793458781	0.793	0.955	1.346	0.678
ENSG00(	COX10	eqtl-a-EN	ebi-a-GC	Systemic	Inverse va	2	-0.220878063	0.117988833	0.061203123	0.061	0.802	1.01	0.636
ENSG00(	ELAC2	eqtl-a-EN	ebi-a-GC	Systemic	Simple m	3	-0.127072303	0.168056123	0.528497894	0.528	0.881	1.224	0.634
ENSG00(	ELAC2	eqtl-a-EN	ebi-a-GC	Systemic	MR Egge	3	-0.110131891	0.298647012	0.77508408	0.775	0.896	1.608	0.499
ENSG00(	ELAC2	eqtl-a-EN	ebi-a-GC	Systemic	Inverse va	3	0.02447803	0.085995807	0.775918325	0.776	1.025	1.213	0.866
ENSG00(	ELAC2	eqtl-a-EN	ebi-a-GC	Systemic	Weighted	3	0.013814075	0.091414018	0.893750063	0.894	1.014	1.213	0.848
ENSG00(	ELAC2	eqtl-a-EN	ebi-a-GC	Systemic	Weighted	3	0.011164471	0.087322526	0.89826509	0.898	1.011	1.2	0.852
ENSG00(	AASS	eqtl-a-EN	ebi-a-GC	Systemic	Inverse va	2	0.064768279	0.084372797	0.442698662	0.443	1.067	1.259	0.904
ENSG00(	MGST1	eqtl-a-EN	ebi-a-GC	Systemic	Weighted	5	0.187989622	0.182217951	0.360516715	0.361	1.207	1.725	0.844
ENSG00(	MGST1	eqtl-a-EN	ebi-a-GC	Systemic	Inverse va	5	0.121442932	0.115890236	0.294678503	0.295	1.129	1.417	0.9
ENSG00(	MGST1	eqtl-a-EN	ebi-a-GC	Systemic	Weighted	5	0.122697753	0.137840551	0.373389296	0.373	1.131	1.481	0.863
ENSG00(	MGST1	eqtl-a-EN	ebi-a-GC	Systemic	Simple m	5	0.187989622	0.199900111	0.400248818	0.4	1.207	1.786	0.816
ENSG00(	MGST1	eqtl-a-EN	ebi-a-GC	Systemic	MR Egge	5	0.593486412	0.404447308	0.238556155	0.239	1.81	4	0.819
ENSG00(	CRY1	eqtl-a-EN	ebi-a-GC	Systemic	Wald ratio	1	-0.053844943	0.131560748	0.682335532	0.682	0.948	1.226	0.732
ENSG00(	LARS2	eqtl-a-EN	ebi-a-GC	Systemic	Inverse va	2	0.0343392	0.064419418	0.59399436	0.594	1.035	1.174	0.912
ENSG00(	SLC25A3	eqtl-a-EN	ebi-a-GC	Systemic	Weighted	6	0.1124216	0.175523314	0.550047403	0.55	1.119	1.578	0.793
ENSG00(	SLC25A3	eqtl-a-EN	ebi-a-GC	Systemic	Simple m	6	-0.093423321	0.240923449	0.714143649	0.714	0.911	1.461	0.568
ENSG00(	SLC25A3	eqtl-a-EN	ebi-a-GC	Systemic	MR Egge	6	0.550993544	0.411746821	0.251841054	0.252	1.735	3.888	0.774
ENSG00(	SLC25A3	eqtl-a-EN	ebi-a-GC	Systemic	Inverse va	6	-0.011776636	0.128497655	0.926977199	0.927	0.988	1.271	0.768
ENSG00(	SLC25A3	eqtl-a-EN	ebi-a-GC	Systemic	Weighted	6	0.105483487	0.156297693	0.499746723	0.5	1.111	1.51	0.818
ENSG00(	HEBP1	eqtl-a-EN	ebi-a-GC	Systemic	Weighted	3	-0.165446188	0.073947296	0.025263292	0.025	0.848	0.98	0.733
ENSG00(	HEBP1	eqtl-a-EN	ebi-a-GC	Systemic	Weighted	3	-0.16697943	0.073127053	0.149846639	0.15	0.846	0.977	0.733
ENSG00(	HEBP1	eqtl-a-EN	ebi-a-GC	Systemic	MR Egge	3	-0.184194143	0.322250343	0.669425239	0.669	0.832	1.564	0.442
ENSG00(	HEBP1	eqtl-a-EN	ebi-a-GC	Systemic	Simple m	3	-0.147090851	0.120796928	0.347515189	0.348	0.863	1.094	0.681
ENSG00(	HEBP1	eqtl-a-EN	ebi-a-GC	Systemic	Inverse va	3	-0.165162813	0.07012419	0.018508248	0.019	0.848	0.973	0.739
ENSG00(	SLC30A9	eqtl-a-EN	ebi-a-GC	Systemic	Inverse va	2	-0.104331365	0.157750331	0.508374945	0.508	0.901	1.227	0.661
ENSG00(	BID	eqtl-a-EN	ebi-a-GC	Systemic	Inverse va	2	-0.087271734	0.123868018	0.481087701	0.481	0.916	1.168	0.719
ENSG00(	CHDH	eqtl-a-EN	ebi-a-GC	Systemic	Wald ratio	1	-0.142553033	0.512225487	0.780781057	0.781	0.867	2.366	0.318
ENSG00(	CYP24A1	eqtl-a-EN	ebi-a-GC	Systemic	Inverse va	2	0.018783438	0.124793596	0.880357495	0.88	1.019	1.301	0.798
ENSG00(	NDUFS1	eqtl-a-EN	ebi-a-GC	Systemic	Inverse va	2	-0.495861738	0.198579176	0.0125232	0.013	0.609	0.899	0.413
ENSG00(	GLRX2	eqtl-a-EN	ebi-a-GC	Systemic	Inverse va	2	0.305654448	0.193901344	0.114947463	0.115	1.358	1.985	0.928
ENSG00(	TOMM34	eqtl-a-EN	ebi-a-GC	Systemic	Weighted	4	0.045676111	0.056834616	0.42158926	0.422	1.047	1.17	0.936
ENSG00(	TOMM34	eqtl-a-EN	ebi-a-GC	Systemic	Simple m	4	0.038692804	0.085938914	0.683086467	0.683	1.039	1.23	0.878
ENSG00(	TOMM34	eqtl-a-EN	ebi-a-GC	Systemic	MR Egge	4	0.008388913	0.114478753	0.948253193	0.948	1.008	1.262	0.806
ENSG00(	TOMM34	eqtl-a-EN	ebi-a-GC	Systemic	Weighted	4	0.037261988	0.058009714	0.566366914	0.566	1.038	1.163	0.926
ENSG00(	TOMM34	eqtl-a-EN	ebi-a-GC	Systemic	Inverse va	4	0.02860088	0.055197168	0.604347705	0.604	1.029	1.147	0.923
ENSG00(	AGPAT4	eqtl-a-EN	ebi-a-GC	Systemic	Weighted	4	0.077629393	0.122305603	0.525613283	0.526	1.081	1.373	0.85
ENSG00(	AGPAT4	eqtl-a-EN	ebi-a-GC	Systemic	Simple m	4	0.090527769	0.215495392	0.702692083	0.703	1.095	1.67	0.718
ENSG00(	AGPAT4	eqtl-a-EN	ebi-a-GC	Systemic	Inverse va	4	0.070233803	0.120722092	0.560714297	0.561	1.073	1.359	0.847
ENSG00(	AGPAT4	eqtl-a-EN	ebi-a-GC	Systemic	Weighted	4	0.077309066	0.131522624	0.597981438	0.598	1.08	1.398	0.835
ENSG00(	AGPAT4	eqtl-a-EN	ebi-a-GC	Systemic	MR Egge	4	0.048507224	0.327548858	0.895852907	0.896	1.05	1.995	0.552
ENSG00(	MIPEP	eqtl-a-EN	ebi-a-GC	Systemic	Wald ratio	1	-0.250358937	0.172703342	0.147156893	0.147	0.779	1.092	0.555
ENSG00(	TFB1M	eqtl-a-EN	ebi-a-GC	Systemic	Inverse va	2	0.086337286	0.107313099	0.421087298	0.421	1.09	1.345	0.883
ENSG00(	BAK1	eqtl-a-EN	ebi-a-GC	Systemic	MR Egge	3	-0.006102793	0.346618635	0.988792417	0.989	0.994	1.961	0.504
ENSG00(	BAK1	eqtl-a-EN	ebi-a-GC	Systemic	Weighted	3	0.086905764	0.049148059	0.077020109	0.077	1.091	1.201	0.991
ENSG00(	BAK1	eqtl-a-EN	ebi-a-GC	Systemic	Simple m	3	0.127406062	0.11352953	0.378397648	0.378	1.136	1.419	0.909
ENSG00(	BAK1	eqtl-a-EN	ebi-a-GC	Systemic	Weighted	3	0.083644108	0.049740774	0.234669399	0.235	1.087	1.199	0.986

Table S10.Testing for heterogeneity and horizontal pleiotropy in the positive exposure of 36 eQTLs

exposure	method	Q	Q_df	Q_pval	gger_intercep	se	pval
ATP5MPL	IVW	0.152	1.000	0.697	NA	NA	NA
ATPAF2	IVW	0.058	1.000	0.810	NA	NA	NA
BAX	IVW	0.301	1.000	0.583	NA	NA	NA
BIK	IVW	2.464	1.000	0.116	NA	NA	NA
C6orf136	IVW	0.202	1.000	0.653	NA	NA	NA
CASP9	MR Egger	0.455	1.000	0.500	-0.033	0.115	0.824
	IVW	0.535	2.000	0.765			
CAT	MR Egger	0.038	1.000	0.846	-0.072	0.048	0.375
	IVW	2.276	2.000	0.321			
CHCHD2	IVW	0.696	3.000	0.874	0.030	0.038	0.512
	MR Egger	0.070	2.000	0.966			
CMPK2	IVW	6.811	2.000	0.033	0.109	0.045	0.249
	MR Egger	0.920	1.000	0.337			
COX20	IVW	2.045	1.000	0.153	NA	NA	NA
GLS2	IVW	2.465	1.000	0.116			
HEBP1	IVW	0.008	2.000	0.996	0.008	0.129	0.962
	MR Egger	0.004	1.000	0.950			
HIBCH	MR Egger	4.816	2.000	0.090	0.002	0.033	0.953
	IVW	4.827	3.000	0.185			
METTL4	IVW	0.398	1.000	0.528	NA	NA	NA
MFN2	IVW	2.320	4.000	0.677	0.032	0.035	0.422
	MR Egger	1.459	3.000	0.692			
MGST3	IVW	1.365	4.000	0.850	0.028	0.047	0.591
	MR Egger	1.006	3.000	0.800			
MPV17L2	IVW	0.235	1.000	0.628	NA	NA	NA
MSRA	IVW	6.803	8.000	0.558	0.005	0.033	0.884
	MR Egger	6.781	7.000	0.452			
MTERF2	IVW	0.815	1.000	0.367	NA	NA	NA
NDUFS1	IVW	0.339	1.000	0.561			
NIPSNAP3A	IVW	0.358	2.000	0.836	-0.002	0.041	0.969
	MR Egger	0.356	1.000	0.551			
PAICS	IVW	0.030	1.000	0.862	NA	NA	NA
PDK4	IVW	1.524	2.000	0.467	-0.210	0.170	0.434
	MR Egger	0.004	1.000	0.948			
PDSS1	IVW	0.386	1.000	0.534	NA	NA	NA
PGS1	MR Egger	1.058	1.000	0.304	-0.027	0.048	0.675
	IVW	1.389	2.000	0.499			
PISD	IVW	4.150	5.000	0.528	0.019	0.023	0.461
	MR Egger	3.487	4.000	0.480			
POLDIP2	IVW	2.313	1.000	0.128	NA	NA	NA
PPTC7	MR Egger	0.180	2.000	0.914	-0.088	0.044	0.184
	IVW	4.152	3.000	0.246			
SDHC	IVW	2.007	1.000	0.157	NA	NA	NA
SLC25A29	IVW	0.155	2.000	0.925	0.010	0.058	0.890
	MR Egger	0.125	1.000	0.724			
SPATA20	IVW	0.656	3.000	0.884	0.015	0.041	0.749
	MR Egger	0.522	2.000	0.770			
SUOX	IVW	2.207	3.000	0.531	0.165	0.118	0.297
	MR Egger	0.254	2.000	0.881			
TOP3A	IVW	1.427	1.000	0.232	NA	NA	NA
TST	IVW	5.832	7.000	0.559	0.025	0.025	0.370
	MR Egger	4.892	6.000	0.558			
VARS2	IVW	6.146	2.000	0.046	-0.028	0.108	0.838
	MR Egger	5.754	1.000	0.016			
VWA8	IVW	0.002	1.000	0.968	NA	NA	NA

Table S11.Summary data-level Mendelian randomization analysis for association between mitochondrial gene expression and systemic lupus erythematosus

Gene	Genenam	probeID	ProbeChr	Probe_bp	topSNP	topSNP_(	topSNP_bp	A1	A2	Freq	b_GWAS	se_GWAS	p_GWAS	b_eQTL	se_eQTL	p_eQTL	b_SMR	se_SMR	p_SMR	p_HEIDI	nsnp_HEIDI
ENSG00(	LAP3	ENSG00(	4	17594205	rs377592	4	17599837	G	A	0.346918	0.0618754	0.0271542	0.02268663	0.786803	0.00744609	0	0.0786416	0.0345201	0.02271835	0.593172	20
ENSG00(	SPATA2(	ENSG00(	17	48626816	rs989020	17	48624523	C	A	0.349901	0.0833816	0.033068	0.01168503	-1.00849	0.00674709	0	-0.0826794	0.0327942	0.01169685	0.07816728	20
ENSG00(	FAM136A	ENSG00(	2	70526164	rs670875	2	70514819	G	A	0.314115	-0.0676586	0.0299072	0.02367936	-0.193636	0.00836102	1.17E-118	0.349411	0.155186	0.02434959	0.4392221	20
ENSG00(	CASP8	ENSG00(	2	202125300	rs756032	2	202164837	A	C	0.385686	-0.0582689	0.0284645	0.04065088	-0.21219	0.00808379	7.38E-152	0.274607	0.134554	0.04126318	0.02386138	20
ENSG00(	POLB	ENSG00(	8	42212649	rs227273	8	42157902	T	C	0.124254	-0.10436	0.0458639	0.02288021	-0.249559	0.0118075	3.74E-99	0.418178	0.184842	0.02367548	0.9898028	20
ENSG00(	DLD	ENSG00(	7	107551795	rs171546	7	107559732	T	G	0.126243	-0.113329	0.0460416	0.01383821	0.132236	0.0119575	1.99E-28	-0.857024	0.356699	0.01627665	0.9174938	20
ENSG00(	ACOT7	ENSG00(	1	6389392	rs490855	1	6463999	G	C	0.0904573	-0.113329	0.0518367	0.02879652	0.33236	0.0148767	1.48E-110	-0.340983	0.156711	0.02956454	0.2145383	20
ENSG00(	SPTLC2	ENSG00(	14	78027728	rs227258	14	78023519	C	G	0.427435	0.0618754	0.0303099	0.0412088	0.370514	0.0077674	0	0.166999	0.0818798	0.04139432	0.607093	20
ENSG00(	PXMP4	ENSG00(	20	32301318	rs274755	20	32299042	A	C	0.481113	0.0618754	0.0301398	0.04007744	-0.0718816	0.00794939	1.53E-19	-0.860796	0.429969	0.04528492	0.198148	20
ENSG00(	SLC25A1	ENSG00(	13	41373897	rs112420	13	41276833	A	G	0.184891	0.0943107	0.0434138	0.02982772	-0.118294	0.0150704	4.18E-15	-0.797258	0.380796	0.03628981	0.7888078	20
ENSG00(	VWA8	ENSG00(	13	42338108	rs128668	13	42152254	T	C	0.148111	-0.0861777	0.0402903	0.03244218	-0.198331	0.0125135	1.42E-56	0.434514	0.204988	0.03403101	0.3020742	20
ENSG00(	GARS1	ENSG00(	7	30653973	rs252944	7	30560535	G	A	0.0675944	-0.10436	0.0523573	0.04623704	-0.199021	0.0154076	3.61E-38	0.524368	0.266189	0.04884848	0.996699	20
ENSG00(	DHRS7B	ENSG00(	17	21061627	rs124523	17	21033736	T	C	0.435388	0.0618754	0.0297258	0.03738436	-0.131884	0.007992	3.55E-61	-0.469165	0.22718	0.03890654	0.04922415	20
ENSG00(	MRPL51	ENSG00(	12	6602078	rs476460	12	6611078	C	T	0.309145	-0.076961	0.0317522	0.0153589	-0.196189	0.00873785	1.20E-111	0.392279	0.162785	0.01596127	0.4943116	20
ENSG00(	MRPL19	ENSG00(	2	75895943	rs116928	2	75896630	A	C	0.242545	0.0943107	0.0355456	0.0079726	-0.324255	0.00892133	3.04E-289	-0.290854	0.109914	0.008140406	0.076919	20
ENSG00(	IDE	ENSG00(	10	94272637	rs139796	10	94310394	A	C	0.0159046	-0.270027	0.105499	0.01048142	0.631689	0.0747791	2.98E-17	-0.427468	0.174509	0.01430353	0.01686257	12
ENSG00(	MTIF3	ENSG00(	13	28017257	rs214681	13	27997305	T	C	0.447316	-0.0676586	0.0302553	0.02533495	0.217774	0.011782	2.80E-76	-0.310683	0.139943	0.02641423	0.3881361	20
ENSG00(	MPST	ENSG00(	22	37420769	rs207593	22	37266322	A	G	0.305169	0.0618754	0.030128	0.04	0.0792236	0.00879246	2.05E-19	0.781023	0.390044	0.04524233	0.727173	20
ENSG00(	COX4I1	ENSG00(	16	85836444	rs96603	16	85815097	C	T	0.198807	-0.0861777	0.0347587	0.01316346	0.207191	0.0105277	3.16E-86	-0.415933	0.169087	0.01389872	0.05623832	20
ENSG00(	CASP9	ENSG00(	1	15835178	rs668564	1	15825195	C	T	0.297217	0.105361	0.031117	0.000709349	0.334371	0.00838694	0	0.315102	0.0933962	0.000741337	0.745254	20
ENSG00(	TIMM17A	ENSG00(	1	201932204	rs7513	1	201938995	T	C	0.467197	0.0725707	0.0298788	0.01514747	0.0692802	0.00797644	3.77E-18	1.0475	0.44782	0.01933024	0.5787691	20
ENSG00(	CARS2	ENSG00(	13	111329854	rs440500	13	111300799	G	A	0.204771	-0.0676586	0.0342782	0.04840386	-0.425572	0.0145374	2.20E-188	0.158983	0.0807292	0.04891439	0.419192	20
ENSG00(	NIPSNAF	ENSG00(	9	107516186	rs108207	9	107524390	C	T	0.462227	-0.0582689	0.0260802	0.0254683	-0.452544	0.00765282	0	0.128759	0.0576714	0.02557356	0.3764208	20
ENSG00(	FPGS	ENSG00(	9	130566741	rs108193	9	130581723	A	G	0.429423	-0.0676586	0.0275704	0.01412668	0.0681953	0.0084896	9.53E-16	-0.992131	0.422732	0.01892772	0.1340068	20
ENSG00(	MRPL15	ENSG00(	8	55054115	rs450618	8	54962327	C	G	0.182903	-0.0861777	0.0366697	0.01876809	0.170506	0.0115978	6.30E-49	-0.505423	0.217795	0.02030609	0.1586351	20
ENSG00(	PNPT1	ENSG00(	2	55891222	rs782642	2	55928444	G	T	0.481113	0.0618754	0.0311278	0.04683494	0.153858	0.00790334	2.08E-84	0.40216	0.203368	0.04798472	0.8258913	20
ENSG00(	SUOX	ENSG00(	12	56395694	rs187391	12	56379427	C	G	0.390656	-0.0582689	0.0285195	0.04104025	-0.336612	0.00796648	0	0.173104	0.0848242	0.04127662	0.03570309	20
ENSG00(	CYP11A1	ENSG00(	15	74645090	rs427869	15	74664951	T	C	0.162028	-0.10436	0.0397184	0.008601615	0.0826582	0.0113735	3.66E-13	-1.26255	0.510953	0.0134748	0.3645513	20
ENSG00(	NDUFB1(	ENSG00(	16	2010742	rs758335	16	2011126	A	G	0.188867	-0.0676586	0.0341689	0.0476892	-0.241118	0.0119429	1.22E-90	0.280604	0.14239	0.04876201	0.2803258	20
ENSG00(	MGST3	ENSG00(	1	165615565	rs414759	1	165600606	T	C	0.452286	-0.0582689	0.0288225	0.04321256	-0.80994	0.00710808	0	0.0719422	0.0355916	0.0432458	0.6041114	20
ENSG00(	PPOX	ENSG00(	1	161142001	rs120314	1	161126975	G	C	0.399602	-0.0582689	0.0276402	0.03502031	-0.185975	0.00821103	1.41E-113	0.313316	0.149266	0.03581259	0.04144062	20
ENSG00(	MCL1	ENSG00(	1	150549549	rs497096	1	150584103	T	G	0.241551	-0.0676586	0.0325496	0.0376513	-0.220546	0.00971457	4.22E-114	0.306778	0.148204	0.03845492	0.3096448	20
ENSG00(	SLC25A3	ENSG00(	3	39431840	rs730602	3	39446889	C	T	0.156064	0.0943107	0.0415712	0.02328949	0.301777	0.0249729	1.28E-33	0.312518	0.140161	0.02576725	0.4780404	20
ENSG00(	NDUFS6	ENSG00(	5	1809116	rs131871	5	1799865	A	G	0.12326	-0.0861777	0.0401858	0.03199411	-0.1029	0.01754	4.45E-09	0.837487	0.415805	0.04399539	0.9551233	20
ENSG00(	PDSS1	ENSG00(	10	27011157	rs174834	10	26974263	G	A	0.308151	-0.076961	0.031782	0.01545539	0.325315	0.00841312	0	-0.236574	0.0978874	0.0156579	0.8409684	20
ENSG00(	OXSM	ENSG00(	3	25830216	rs222044	3	25744483	A	G	0.196819	-0.076961	0.0373636	0.03941849	0.116793	0.0111682	1.35E-25	-0.658954	0.326061	0.04328426	0.3745733	20
ENSG00(	BCL2L11	ENSG00(	2	111901489	rs113135	2	111887754	G	T	0.117296	-0.139762	0.0446566	0.001749806	0.164709	0.0127383	3.04E-38	-0.84854	0.278954	0.002351153	0.1727808	20
ENSG00(	MRPL39	ENSG00(	21	26968898	rs282980	21	26960189	T	G	0.264414	-0.0676586	0.0341857	0.04779913	-0.199368	0.00875903	1.11E-114	0.339366	0.172118	0.04864278	0.5296066	20
ENSG00(	OXNAD1	ENSG00(	3	16342764	rs552	3	16301959	A	G	0.162028	-0.113329	0.0398759	0.004482703	0.208718	0.0112695	1.41E-76	-0.542977	0.193288	0.004967094	0.5533847	20
ENSG00(	NDUFS2	ENSG00(	1	161175539	rs437969	1	161186313	T	C	0.319085	-0.0582689	0.0287645	0.04279273	-0.225171	0.00863498	6.72E-150	0.258776	0.12813	0.04342097	0.5621551	20
ENSG00(	COX6B2	ENSG00(	19	55863428	rs124616	19	55865350	G	A	0.463221	-0.0582689	0.0281471	0.03843793	-0.0946239	0.00966319	1.22E-22	0.615795	0.304038	0.04282732	0.02670839	17
ENSG00(	RPUSD4	ENSG00(	11	126076790	rs630956	11	126078600	A	C	0.197813	0.105361	0.0343503	0.002160479	-0.523821	0.00950582	0	-0.201139	0.0656779	0.002194886	0.1149034	20
ENSG00(	COMTD1	ENSG00(	10	76994757	rs792327	10	76962774	A	C	0.0506958	0.235722	0.0655505	0.000323095	0.630461	0.0173128	2.39E-290	0.373888	0.104478	0.000345399	0.02278594	20
ENSG00(	METTL17	ENSG00(	14	21461559	rs274172	14	21456092	A	C	0.310139	0.0618754	0.0298248	0.03802069	-0.327201	0.00928267	3.60E-272	-0.189105	0.091309	0.03835459	0.1574729	20
ENSG00(	COQ7	ENSG00(	16	19085169	rs463053	16	19080184	C	T	0.377734	-0.076961	0.0289864	0.007929029	0.228832	0.00798393	1.15E-180	-0.336321	0.127213	0.008199233	0.8704244	20
ENSG00(	DUS2	ENSG00(	16	68067436	rs649915	16	68105474	C	A	0.183897	-0.076961	0.0340556	0.02382978	0.784482	0.0105154	0	-0.0981042	0.0434315	0.02389414	0.04712693	20
ENSG00(	PRDX2	ENSG00(	19	12910164	rs897804	19	12876964	C	G	0.439364	0.0833816	0.0316284	0.008381659	0.235476	0.00955092	3.27E-134	0.354098	0.135082	0.008758338	0.1574785	20
ENSG00(	AHCYL1	ENSG00(	1	110546832	rs375444	1	110534462	A	G	0.16501	-0.0861777	0.0376376	0.02204043	-0.126236	0.0100747	5.12E-36	0.682674	0.303091	0.02429868	0.5996056	20
ENSG00(	NFU1	ENSG00(	2	69643821	rs434643	2	69668802	A	G	0.38668	0.0618754	0.0299913	0.03910209	0.267776	0.0119309	1.47E-111	0.231072	0.112474	0.03993217	0.176177	20
ENSG00(	NUDT6	ENSG00(	4	123828926	rs768309	4	123818085	G	C	0.132207	0.127833	0.0395241	0.001219326	0.281864	0.0118737	1.44E-124	0.453527	0.141519	0.001352046	0.3675599	20
ENSG00(	GATM	ENSG00(	15	45673869	rs716460	15	45671325	G	A	0.275348	-0.0676586	0.0305003	0.02653444	-0.576781	0.00822595	0	0.117304	0.0529067	0.02661055	0.4417127	20
ENSG00(	ATPAF2	ENSG00(	17	17911623	rs339971	17	17921995	T	C	0.0427435	0.18633	0.0799519	0.0197788	-0.791058	0.0217364	5.43E-290	-0.235545	0.101277	0.02003123	0.5299698	20
ENSG00(	DCAKD	ENSG00(	17	43119590	rs558977	17	43136303	T	C	0.417495	0.0618754	0.0305859	0.04307249	-0.497154	0.00834366	0	-0.124459	0.0615574	0.04319298	0.2248476	20
ENSG00(	ABCD2	ENSG00(	12	39978694	rs713337	12	39982882	A	G	0.164016	-0.0861777	0.0375122	0.02160031	-0.217787	0.0111642	9.46E-85	0.395697	0.173433	0.02251545	0.3119079	20
ENSG00(	MSRA	ENSG00(	8	10099089	rs117773	8	10288546	C	G	0.451292	0.0833816	0.0318395	0.008823685	0.319316	0.00790221	0	0.261125	0.0999207	0.008966532	0.6961632	20
ENSG00(	DDX28	ENSG00(	16	68056474	rs720419	16	68039309	G	A	0.150099	-0.122218	0.0412963	0.00308113	0.0950532	0.0110805	9.62E-18	-1.28578	0.459583	0.005146467	0.7700987	20
ENSG00(	COA5	ENSG00(	2	99220375	rs376969	2	99233894	C	T	0.271372	0.0833816	0.0318476	0.008840768	0.191846	0.010327	4.91E-77	0.434627	0.167646	0.009527455	0.5314015	20
ENSG00(	NDUFA1:	ENSG00(	19	19635415	rs110852	19	19621780	G	A	0.16998	-0.0676586	0.0337626	0.04507544	0.192874	0.0128241	4.02E-51	-0.350791	0.176597	0.04698935	0.493487	20
ENSG00(	PPTC7	ENSG00(	12	110995122	rs729434	12	110884380	G	A	0.11829	0.127833	0.0467999	0.006304926	-0.2348	0.014421	1.33E-59	-0.544434	0.202104	0.00706351	0.1934815	20
ENSG00(	ATAD3A	ENSG00(	1	1458799	rs376617	1	1497008	C	T	0.346918	0.0943107	0.0307244	0.00214368	-0.13413	0.0097562	5.22E-43	-0.703128	0.234704	0.002737187	0.6234767	20
ENSG00(	HIBCH	ENSG00(	2	191131690	rs291460	2	191181585	G	A	0.40159	-0.10436	0.0312199	0.000829564	-0.415124	0.00780058	0	0.251395	0.0753544	0.000849428	0.04509647	20
ENSG00(	SYNJ2BF	ENSG00(	14	70860963	rs619795	14	70745723	T	G	0.0626243	0.150823	0.0514816	0.003393517	-0.142404	0.0157009	1.19E-19	-1.05912	0.379909	0.00530639	0.9978139	20
ENSG00(	ATP5PO	ENSG00(	21	35282020	rs731058	21	35275199	G	A	0.395626	-0.10436	0.0304861	0.000618899	-0.148453	0.00807369	1.66E-75	0.702986	0.208888	0.000764401	0.1537658	20
ENSG00(	MARS2	ENSG00(	2	198571600	rs234151	2	198558679	T	C	0.513917	-0.0676586	0.0278201	0.01501516	0.202769	0.00787503	3.36E-146	-0.333673	0.137811	0.01546818	0.4418973	20
ENSG00(	MPV17L2	ENSG00(	19	18305875	rs227188	19	18305824	T	C	0.353877	0.105361	0.0305531	0.000563832	0.143401	0.00837478	1.00E-65	0.734728	0.217338	0.000723331	0.2332586	20

Table S12. pQTL-harm_32 pQTLs of mitochondria-related genes.

SNP	effect_allele.	other_allele.	effect_allele.	other_allele.	beta.exposur	beta.outcome	eaf.exposure	eaf.outcome	remove	palindromic	ambiguous	id.outcome	chr	pos	se.outcome	samplesize.c	pval.outcome	outcome	originalname	outcome.dep	mr_keep.out	data_source
rs10305656	C	G	C	G	0.3511	-0.0833816	0.02008	NA	FALSE	TRUE	TRUE	ebi-a-GCST(	1	150840520	0.108356	14267	0.441586	Systemic lup	Systemic lup	Systemic lup	TRUE	igd
rs60162170	A	G	A	G	-0.078	0.0582689	0.1282	NA	FALSE	FALSE	FALSE	ebi-a-GCST(	1	150511144	0.0403042	14267	0.148254	Systemic lup	Systemic lup	Systemic lup	TRUE	igd
rs10814111	A	C	A	C	0.1822	-0.0100503	0.07849	NA	FALSE	FALSE	FALSE	ebi-a-GCST(	9	34457852	0.0465878	14267	0.829199	Systemic lup	Systemic lup	Systemic lup	TRUE	igd
rs111577519	A	G	A	G	-0.0729	0.076961	0.14021	NA	FALSE	FALSE	FALSE	ebi-a-GCST(	9	33499934	0.0426612	14267	0.0712312	Systemic lup	Systemic lup	Systemic lup	TRUE	igd
rs7025269	T	C	T	C	0.2999	-0.0198026	0.20745	NA	FALSE	FALSE	FALSE	ebi-a-GCST(	9	34336266	0.0365359	14267	0.587815	Systemic lup	Systemic lup	Systemic lup	TRUE	igd
rs10853721	G	A	G	A	-0.0787	-0.040822	0.12994	NA	FALSE	FALSE	FALSE	ebi-a-GCST(	19	39312496	0.0448915	14267	0.363166	Systemic lup	Systemic lup	Systemic lup	TRUE	igd
rs4802890	A	G	A	G	0.4475	0.0392207	0.08781	NA	FALSE	FALSE	FALSE	ebi-a-GCST(	19	39304402	0.0496201	14267	0.429283	Systemic lup	Systemic lup	Systemic lup	TRUE	igd
rs73544031	C	T	C	T	0.2006	0.0295588	0.01294	NA	FALSE	FALSE	FALSE	ebi-a-GCST(	19	39771267	0.1025	14267	0.773058	Systemic lup	Systemic lup	Systemic lup	TRUE	igd
rs10939687	T	C	T	C	-0.1428	0.0676586	0.0379	NA	FALSE	FALSE	FALSE	ebi-a-GCST(	4	16734098	0.0634128	14267	0.285992	Systemic lup	Systemic lup	Systemic lup	TRUE	igd
rs141974152	A	G	A	G	-0.4471	0.14842	0.01204	NA	FALSE	FALSE	FALSE	ebi-a-GCST(	4	18163701	0.155812	14267	0.340813	Systemic lup	Systemic lup	Systemic lup	TRUE	igd
rs10939687	T	C	T	C	-0.1408	0.0676586	0.0379	NA	FALSE	FALSE	FALSE	ebi-a-GCST(	4	16734098	0.0634128	14267	0.285992	Systemic lup	Systemic lup	Systemic lup	TRUE	igd
rs141974152	A	G	A	G	-0.4368	0.14842	0.01204	NA	FALSE	FALSE	FALSE	ebi-a-GCST(	4	18163701	0.155812	14267	0.340813	Systemic lup	Systemic lup	Systemic lup	TRUE	igd
rs10973326	A	G	A	G	0.2604	-0.0100503	0.32781	NA	FALSE	FALSE	FALSE	ebi-a-GCST(	9	37419497	0.0230595	14267	0.66295	Systemic lup	Systemic lup	Systemic lup	TRUE	igd
rs72737776	G	A	G	A	-0.1577	-0.105361	0.04236	NA	FALSE	FALSE	FALSE	ebi-a-GCST(	9	37279578	0.0810669	14267	0.193713	Systemic lup	Systemic lup	Systemic lup	TRUE	igd
rs7874199	A	G	A	G	0.0644	0.0198026	0.22902	NA	FALSE	FALSE	FALSE	ebi-a-GCST(	9	37698871	0.0303234	14267	0.513727	Systemic lup	Systemic lup	Systemic lup	TRUE	igd
rs11025480	A	G	A	G	0.3213	0.0676586	0.21333	NA	FALSE	FALSE	FALSE	ebi-a-GCST(	11	20326091	0.0302247	14267	0.0251866	Systemic lup	Systemic lup	Systemic lup	TRUE	igd
rs1356981	G	A	G	A	0.0861	0.0202027	0.44745	NA	FALSE	FALSE	FALSE	ebi-a-GCST(	11	20243291	0.0353392	14267	0.567539	Systemic lup	Systemic lup	Systemic lup	TRUE	igd
rs1715284	T	G	T	G	-0.1287	-0.0202027	0.19712	NA	FALSE	FALSE	FALSE	ebi-a-GCST(	11	20687297	0.048871	14267	0.679323	Systemic lup	Systemic lup	Systemic lup	TRUE	igd
rs2003174	C	G	C	G	-0.1066	-0.0100503	0.09513	NA	FALSE	TRUE	TRUE	ebi-a-GCST(	11	20992139	0.083163	14267	0.903809	Systemic lup	Systemic lup	Systemic lup	TRUE	igd
rs3758807	A	G	A	G	-0.1256	-0.0304592	0.35978	NA	FALSE	FALSE	FALSE	ebi-a-GCST(	11	20638531	0.0325488	14267	0.349376	Systemic lup	Systemic lup	Systemic lup	TRUE	igd
rs79225249	C	G	C	G	0.147	0.0676586	0.05609	NA	FALSE	TRUE	TRUE	ebi-a-GCST(	11	20045577	0.0582007	14267	0.24503	Systemic lup	Systemic lup	Systemic lup	TRUE	igd
rs11055175	G	A	G	A	-1.33	0.0295588	0.02799	NA	FALSE	FALSE	FALSE	ebi-a-GCST(	12	13121778	0.0721117	14267	0.681877	Systemic lup	Systemic lup	Systemic lup	TRUE	igd
rs12368093	C	T	C	T	-0.0705	-0.0512933	0.25065	NA	FALSE	FALSE	FALSE	ebi-a-GCST(	12	13403073	0.0330665	14267	0.12085	Systemic lup	Systemic lup	Systemic lup	TRUE	igd
rs1379509	C	T	C	T	-0.1409	0.00995033	0.12012	NA	FALSE	FALSE	FALSE	ebi-a-GCST(	12	13534551	0.0321609	14267	0.757023	Systemic lup	Systemic lup	Systemic lup	TRUE	igd
rs73068707	A	T	A	T	-0.1821	-0.0202027	0.11361	NA	FALSE	TRUE	TRUE	ebi-a-GCST(	12	13089880	0.0376457	14267	0.591507	Systemic lup	Systemic lup	Systemic lup	TRUE	igd
rs1122818	G	A	G	A	-0.0729	0.0392207	0.18101	NA	FALSE	FALSE	FALSE	ebi-a-GCST(	3	39141096	0.0324253	14267	0.226444	Systemic lup	Systemic lup	Systemic lup	TRUE	igd
rs2685096	C	A	C	A	-0.0809	-0.0100503	0.36921	NA	FALSE	FALSE	FALSE	ebi-a-GCST(	3	37813594	0.0347427	14267	0.772368	Systemic lup	Systemic lup	Systemic lup	TRUE	igd
rs7625290	G	T	G	T	-0.1782	0.00995033	0.04447	NA	FALSE	FALSE	FALSE	ebi-a-GCST(	3	38178290	0.0585681	14267	0.865094	Systemic lup	Systemic lup	Systemic lup	TRUE	igd
rs115538711	T	A	T	A	-0.1506	-0.0618754	0.02557	NA	FALSE	TRUE	TRUE	ebi-a-GCST(	6	4400945	0.116479	14267	0.595271	Systemic lup	Systemic lup	Systemic lup	TRUE	igd
rs72845845	A	T	A	T	0.1264	0.0392207	0.07023	NA	FALSE	TRUE	TRUE	ebi-a-GCST(	6	3960233	0.058537	14267	0.502848	Systemic lup	Systemic lup	Systemic lup	TRUE	igd
rs115777975	A	G	A	G	-0.2283	0.076961	0.02158	NA	FALSE	FALSE	FALSE	ebi-a-GCST(	5	157410356	0.145461	14267	0.596748	Systemic lup	Systemic lup	Systemic lup	TRUE	igd
rs35323270	T	C	T	C	-0.054	0.00995033	0.36887	NA	FALSE	FALSE	FALSE	ebi-a-GCST(	5	157531320	0.0474802	14267	0.834005	Systemic lup	Systemic lup	Systemic lup	TRUE	igd
rs62387542	GAGACG	GAGAAG	G	T	-0.0947	-0.0100503	0.26901	NA	TRUE	FALSE	FALSE	ebi-a-GCST(	5	156867767	0.0555907	14267	0.856531	Systemic lup	Systemic lup	Systemic lup	TRUE	igd
rs7704907	A	C	A	C	0.2536	-0.0198026	0.46707	NA	FALSE	FALSE	FALSE	ebi-a-GCST(	5	157109244	0.0327246	14267	0.545093	Systemic lup	Systemic lup	Systemic lup	TRUE	igd
rs11580071	A	G	A	G	0.4114	0.0582689	0.11328	NA	FALSE	FALSE	FALSE	ebi-a-GCST(	1	160951832	0.0404926	14267	0.15015	Systemic lup	Systemic lup	Systemic lup	TRUE	igd
rs2255847	C	T	C	T	-0.0621	0.0100503	0.17194	NA	FALSE	FALSE	FALSE	ebi-a-GCST(	1	160926937	0.0324114	14267	0.756495	Systemic lup	Systemic lup	Systemic lup	TRUE	igd
rs78714351	G	A	G	A	0.2011	0.0487902	0.02225	NA	FALSE	FALSE	FALSE	ebi-a-GCST(	1	160805118	0.0848404	14267	0.565236	Systemic lup	Systemic lup	Systemic lup	TRUE	igd
rs116161634	T	C	T	C	-0.6046	0.076961	0.01536	NA	FALSE	FALSE	FALSE	ebi-a-GCST(	1	147088133	0.110141	14267	0.484708	Systemic lup	Systemic lup	Systemic lup	TRUE	igd
rs116509906	A	T	A	T	0.2908	-0.0202027	0.04463	NA	FALSE	TRUE	TRUE	ebi-a-GCST(	1	147323415	0.102013	14267	0.843014	Systemic lup	Systemic lup	Systemic lup	TRUE	igd
rs143333817	G	A	G	A	-0.9248	-0.287682	0.03025	NA	FALSE	FALSE	FALSE	ebi-a-GCST(	1	147027578	0.153523	14267	0.0609481	Systemic lup	Systemic lup	Systemic lup	TRUE	igd
rs202051594	T	C	T	C	-0.7665	-0.0100503	0.0173	NA	FALSE	FALSE	FALSE	ebi-a-GCST(	1	146938450	0.106964	14267	0.925141	Systemic lup	Systemic lup	Systemic lup	TRUE	igd
rs2985722	T	C	T	C	0.0967	0.0295588	0.26603	NA	FALSE	FALSE	FALSE	ebi-a-GCST(	1	146533291	0.038594	14267	0.443741	Systemic lup	Systemic lup	Systemic lup	TRUE	igd
rs59326551	A	T	A	T	-0.3403	0.122218	0.03058	NA	FALSE	TRUE	TRUE	ebi-a-GCST(	1	147303275	0.0945682	14267	0.196227	Systemic lup	Systemic lup	Systemic lup	TRUE	igd
rs76390540	A	G	A	G	-0.6406	-0.105361	0.02492	NA	FALSE	FALSE	FALSE	ebi-a-GCST(	1	147150770	0.0801864	14267	0.188865	Systemic lup	Systemic lup	Systemic lup	TRUE	igd
rs791283	C	T	C	T	0.2377	0.076961	0.03911	NA	FALSE	FALSE	FALSE	ebi-a-GCST(	1	147246725	0.125239	14267	0.538876	Systemic lup	Systemic lup	Systemic lup	TRUE	igd
rs1165681	C	T	C	T	0.0508	0.0295588	0.32009	NA	FALSE	FALSE	FALSE	ebi-a-GCST(	12	104325111	0.0348795	14267	0.396742	Systemic lup	Systemic lup	Systemic lup	TRUE	igd
rs954921	C	T	C	T	0.1674	0.0861777	0.378	NA	FALSE	FALSE	FALSE	ebi-a-GCST(	12	104173630	0.028143	14267	0.00219766	Systemic lup	Systemic lup	Systemic lup	TRUE	igd
rs117789622	T	C	T	C	-0.2892	-0.235722	0.01245	NA	FALSE	FALSE	FALSE	ebi-a-GCST(	21	37597235	0.1659	14267	0.155355	Systemic lup	Systemic lup	Systemic lup	TRUE	igd
rs138308942	G	C	G	C	0.1269	-0.0833816	0.03756	NA	FALSE	TRUE	TRUE	ebi-a-GCST(	21	36751239	0.143815	14267	0.56206	Systemic lup	Systemic lup	Systemic lup	TRUE	igd
rs17814116	C	T	C	T	0.1516	0.131028	0.04304	NA	FALSE	FALSE	FALSE	ebi-a-GCST(	21	37712085	0.0729798	14267	0.0725889	Systemic lup	Systemic lup	Systemic lup	TRUE	igd
rs218634	A	G	A	G	-0.0735	-0.0725707	0.12794	NA	FALSE	FALSE	FALSE	ebi-a-GCST(	21	37794921	0.0404966	14267	0.0731291	Systemic lup	Systemic lup	Systemic lup	TRUE	igd
rs73202262	G	A	G	A	0.2307	-0.0725707	0.03196	NA	FALSE	FALSE	FALSE	ebi-a-GCST(	21	37621927	0.0971328	14267	0.454986	Systemic lup	Systemic lup	Systemic lup	TRUE	igd
rs117799316	T	G	T	G	-0.1252	0.00995033	0.03236	NA	FALSE	FALSE	FALSE	ebi-a-GCST(	16	491484	0.0826706	14267	0.904197	Systemic lup	Systemic lup	Systemic lup	TRUE	igd
rs3848364	G	T	G	T	-0.0617	-0.0833816	0.31988	NA	FALSE	FALSE	FALSE	ebi-a-GCST(	16	372635	0.0331242	14267	0.0118277	Systemic lup	Systemic lup	Systemic lup	TRUE	igd
rs117918535	GA	TA	C	A	-0.1229	-0.040822	0.08579	NA	TRUE	FALSE	FALSE	ebi-a-GCST(	7	86338286	0.0533343	14267	0.444034	Systemic lup	Systemic lup	Systemic lup	TRUE	igd
rs193163017	G	A	G	A	-0.1711	0.076961	0.066	NA	FALSE	FALSE	FALSE	ebi-a-GCST(	7	87742979	0.0480247	14267	0.109038	Systemic lup	Systemic lup	Systemic lup	TRUE	igd
rs7810499	C	T	C	T	0.106	0.0582689	0.06598	NA	FALSE	FALSE	FALSE	ebi-a-GCST(	7	87286446	0.0573873	14267	0.309933	Systemic lup	Systemic lup	Systemic lup	TRUE	igd
rs809066	G	A	G	A	0.5317	0.040822	0.10785	NA	FALSE	FALSE	FALSE	ebi-a-GCST(	7	86987655	0.0491871	14267	0.406577	Systemic lup	Systemic lup	Systemic lup	TRUE	igd
rs12129832	C	T	C	T	-0.187	-0.0618754	0.04225	NA	FALSE	FALSE	FALSE	ebi-a-GCST(	1	192977251	0.0743267	14267	0.405139	Systemic lup	Systemic lup	Systemic lup	TRUE	igd
rs72743118	A	G	A	G	0.3213	-0.0202027	0.01746	NA	FALSE	FALSE	FALSE	ebi-a-GCST(	1	193960610	0.135238	14267	0.881249	Systemic lup	Systemic lup	Systemic lup	TRUE	igd
rs12403088	T	G	T	G	-0.0727	-0.0512933	0.3555	NA	FALSE	FALSE	FALSE	ebi-a-GCST(	1	74236492	0.0325258	14267	0.114795	Systemic lup	Systemic lup	Systemic lup	TRUE	igd
rs13375787	T	C	T	C	0.8701	0.139762	0.03876	NA	FALSE	FALSE	FALSE	ebi-a-GCST(	1	75178713	0.0709364	14267	0.0488101	Systemic lup	Systemic lup	Systemic lup	TRUE	igd
rs277392	G	T	G	T	-0.2865	0.0392207	0.33234	NA	FALSE	FALSE	FALSE	ebi-a-GCST(	1	75282962	0.0306825	14267	0.201152	Systemic lup	Systemic lup	Systemic lup	TRUE	igd
rs72675345	G	A	G	A	-0.4359	0.00995033	0.01434	NA	FALSE	FALSE	FALSE	ebi-a-GCST(	1	74981360	0.0880683	14267	0.910043	Systemic lup	Systemic lup	Systemic lup	TRUE	igd
rs76296629	T	G	T	G	0.479	-0.0100503	0.07364	NA	FALSE	FALSE	FALSE	ebi-a-GCST(	1	75056843	0.0930373	14267	0.913976	Systemic lup	Systemic lup	Systemic lup	TRUE	igd
rs12464143	ATTGTTTT	TTTGTTTT	T	A	0.1279	0.10436	0.05733	NA	TRUE	FALSE	FALSE	ebi-a-GCST(	2	191860502	0.0438116	14267	0.0172179	Systemic lup	Systemic lup	Systemic lup	TRUE	igd
rs62184386	T	C	T	C	0.2417	-0.0618754	0.47584	NA	FALSE	FALSE	FALSE	ebi-a-GCST(	2	191085618	0.0301514	14267	0.0401541	Systemic lup	Systemic lup	Systemic lup	TRUE	igd
rs75949659	A	C	A	C	0.0891	-0.0100503	0.11961	NA	FALSE	FALSE	FALSE	ebi-a-GCST(	2	190264322	0.0694249	14267	0.884896	Systemic lup	Systemic lup	Systemic lup	TRUE	igd
rs785223	T	G	T	G	-0.0584	0.0100503	0.24223	NA	FALSE	FALSE	FALSE	ebi-a-GCST(	2	191002589	0.0279974	14267	0.719615	Systemic lup	Systemic lup	Systemic lup	TRUE	igd
rs12881757	C	G	C	G	0.8177	0.0676586	0.04413	NA	FALSE	TRUE	TRUE	ebi-a-GCST(	14	78118912	0.08592	14267	0.431012	Systemic lup	Systemic lup	Systemic lup	TRUE	igd
rs149721633	T	C	T	C	-0.4557	-0.0618754	0.02536	NA	FALSE	FALSE	FALSE	ebi-a-GCST(	14	77801238	0.0910384	14267	0.496718	Systemic lup	Systemic lup	Systemic lup	TRUE	igd
rs186433582	G	A	G	A	0.7385	0.207014	0.03508	NA	FALSE	FALSE	FALSE	ebi-a-GCST(	14	77479045	0.143197	14267	0.148274	Systemic lup	Systemic lup	Systemic lup	TRUE	igd
rs4467006	G	C	G	C	0.3093	0.0202027	0.29744	NA	FALSE	TRUE	TRUE	ebi-a-GCST(	14	77684670	0.0340162	14267	0.55257	Systemic lup	Systemic lup	Systemic lup	TRUE	igd
rs45608147	G	C	G	C	0.9121	0.10436	0.01974	NA	FALSE	TRUE	TRUE	ebi-a-GCST(	14	77735762	0.0886921	14267	0.239333	Systemic lup	Systemic lup	Systemic lup	TRUE	igd
rs56198483	T	C	T	C	0.8332	0.00995033	0.0127	NA	FALSE	FALSE	FALSE	ebi-a-GCST(	14	77828430	0.116725	14267	0.932066	Systemic lup	Systemic lup	Systemic lup	TRUE	igd
rs755072	G	A	G	A	0.9087	-0.0304592	0.02345	NA	FALSE	FALSE	FALSE	ebi-a-GCST(	14	77771745	0.126947	14267	0.810379	Systemic lup	Systemic lup	Systemic lup	TRUE	igd
rs78371502	T	C	T	C	-0.3559	0.0198026	0.08938	NA	FALSE	FALSE	FALSE	ebi-a-GCST(	14	77951453	0.0534904	14267	0.711227	Systemic lup	Systemic lup	Systemic lup	TRUE	igd
rs79607813	A	G	A	G	-0.2331	-0.150823	0.01703	NA	FALSE	FALSE	FALSE	ebi-a-GCST(	14	77563676	0.092411	14267	0.102661	Systemic lup	Systemic lup	Systemic lup	TRUE	igd
rs1344544	G	A	G	A	0.136	-0.0304592	0.30615	NA	FALSE	FALSE	FALSE	ebi-a-GCST(	12	110082071	0.0338963	14267	0.368866	Systemic lup	Systemic lup	Systemic lup	TRUE	igd
rs16939972	C	T	C	T	0.0948	-0.0582689	0.35016	NA	FALSE	FALSE	FALSE	ebi-a-GCST(	12	109568797	0.0304953	14267	0.0560364	Systemic lup	Systemic lup	Systemic lup	TRUE	igd
rs190825201	A	G	A	G	-0.3106	0.0198026	0.01889	NA	FALSE	FALSE	FALSE	ebi-a-GCST(	12	110106429	0.0799145	14267	0.804291	Systemic lup	Systemic lup	Systemic lup	TRUE	igd
rs4766598	G	A	G	A	-0.305	0.00995033	0.22092	NA	FALSE	FALSE	FALSE	ebi-a-GCST(	12	109881809	0.03619	14267	0.783357	Systemic lup	Systemic lup	Systemic lup	TRUE	igd
rs78925260	A	G	A	G	-0.1893	-0.0725707	0.02683	NA	FALSE	FALSE	FALSE	ebi-a-GCST(	12	110752215	0.127349	14267	0.568774	Systemic lup	Systemic lup	Systemic lup	TRUE	igd
rs1380642	T	C	T	C	-0.3019	0.0487902	0.2272	NA	FALSE	FALSE	FALSE	ebi-a-GCST(	15	80096871	0.0363758	14267	0.179829	Systemic lup	Systemic lup	Systemic lup	TRUE	igd
rs62026657	G	A	G	A	-0.3493	-0.105361	0.06422	NA	FALSE	FALSE	FALSE	ebi-a-GCST(	15	80110999	0.0682857	14267	0.122846	Systemic lup	Systemic lup	Systemic lup	TRUE	igd
rs72736560	A	C	A	C	0.2404	-0.235722	0.02676	NA	FALSE	FALSE	FALSE	ebi-a-GCST(	15	80240840	0.105831	14267	0.0259245	Systemic lup	Systemic lup	Systemic lup	TRUE	igd
rs80102939	T	C	T	C	-0.0723	0.0487902	0.13174	NA	FALSE	FALSE	FALSE	ebi-a-GCST(	15	80675608	0.0405341	14267	0.228713	Systemic lup	Systemic lup	Systemic lup	TRUE	igd
rs144303868	C	G	C	G	0.1579	-0.0833816	0.06011	NA	FALSE	TRUE	TRUE	ebi-a-GCST(	2	242619558	0.0996258	14267	0.402622	Systemic lup	Systemic lup	Systemic lup	TRUE	igd
rs1675513	G	A	G	A	-0.1881	-0.0100503	0.13903	NA	FALSE	FALSE	FALSE	ebi-a-GCST(	3	98318393	0.0349676	14267	0.773792	Systemic lup	Systemic lup	Systemic lup	TRUE	igd
rs1841542	G	A	G	A	0.0795	-0.10436	0.25297	NA	FALSE	FALSE	FALSE	ebi-a-GCST(	3	98348742	0.045772	14267	0.0226079	Systemic lup	Systemic lup	Systemic lup	TRUE	igd
rs17714829	C	T	C	T	0.1255	-0.0512933	0.0781	NA	FALSE	FALSE	FALSE	ebi-a-GCST(	17	48452481	0.0617735	14267	0.406344	Systemic lup	Systemic lup	Systemic lup	TRUE	igd
rs77405995	A	G	A	G	0.1222	0.0676586	0.04892	NA	FALSE	FALSE	FALSE	ebi-a-GCST(	17	48361373	0.0777947	14267	0.38446	Systemic lup	Systemic lup	Systemic lup	TRUE	igd
rs9890200	C	A	C	A	-0.3204	-0.0833816	0.40963	NA	FALSE	FALSE	FALSE	ebi-a-GCST(	17	48624523	0.033068	14267	0.011685	Systemic lup	Systemic lup	Systemic lup	TRUE	igd
rs1861123	T	C	T	C	0.0654	-0.0487902	0.14458	NA	FALSE	FALSE	FALSE	ebi-a-GCST(	4	18286475	0.048035	14267	0.309762	Systemic lup	Systemic lup	Systemic lup	TRUE	igd
rs3822233	A	G	A	G	-0.3944	-0.0304592	0.27499	NA	FALSE	FALSE	FALSE	ebi-a-GCST(	4	17511347	0.0273707	14267	0.265777	Systemic lup	Systemic lup	Systemic lup	TRUE	igd
rs75218392	G	A	G	A	-0.2005	-0.0202027	0.04573	NA	FALSE	FALSE	FALSE	ebi-a-GCST(	4	17402549	0.0634416	14267	0.750146	Systemic lup	Systemic lup	Systemic lup	TRUE	igd
rs2746626	G	C	G	C	-0.0587	0.0100503	0.1753	NA	FALSE	TRUE	TRUE	ebi-a-GCST(	11	34241381	0.0408691	14267	0.805748	Systemic lup	Systemic lup	Systemic lup	TRUE	igd
rs2755171	G	T	G	T	0.0905	-0.105361	0.25301	NA	FALSE	FALSE	FALSE	ebi-a-GCST(	11	34349623	0.0364105	14267	0.0038075	Systemic lup	Systemic lup	Systemic lup	TRUE	igd
rs769218	A	G	A	G	-0.212	0.0198026	0.21994	NA	FALSE	FALSE	FALSE	ebi-a-GCST(	11	34470679	0.0287725	14267	0.491297	Systemic lup	Systemic lup	Systemic lup	TRUE	igd
rs35022330	GGGGCAG(	AGGGCAG(	C	T	-0.2042	0.00995033	0.20531	NA	TRUE	FALSE	FALSE	ebi-a-GCST(	2	173445325	0.0421701	14267	0.813466	Systemic lup	Systemic lup	Systemic lup	TRUE	igd
rs35783685	T	C	T	C	-0.0557	-0.0100503	0.23407	NA	FALSE	FALSE	FALSE	ebi-a-GCST(	2	173322927	0.0265345	14267	0.704863	Systemic lup	Systemic lup	Systemic lup	TRUE	igd
rs4464946	A	G	A	G	-0.2688	0.00995033	0.27394	NA	FALSE	FALSE	FALSE	ebi-a-GCST(	8	87332545	0.0417888	14267	0.811796	Systemic lup	Systemic lup	Systemic lup	TRUE	igd
rs4961042	G	T	G	T	-0.0956	0.0202027	0.39933	NA	FALSE	FALSE	FALSE	ebi-a-GCST(	8	87056585	0.0330083	14267	0.540505	Systemic lup	Systemic lup	Systemic lup	TRUE	igd
rs75979940	A	G	A	G	-0.123	0.00995033	0.05407	NA	FALSE	FALSE	FALSE	ebi-a-GCST(	8	87929935	0.0449767	14267	0.824911	Systemic lup	Systemic lup	Systemic lup	TRUE	igd
rs79117756	G	A	G	A	0.1286	-0.0618754	0.05041	NA	FALSE	FALSE	FALSE	ebi-a-GCST(	8	86499059	0.106951	14267	0.5629	Systemic lup	Systemic lup	Systemic lup	TRUE	igd
rs4733505	G	A	G	A	0.113	0.040822	0.339	NA	FALSE	FALSE	FALSE	ebi-a-GCST(	8	30553430	0.0273415	14267	0.135426	Systemic lup	Systemic lup	Systemic lup	TRUE	igd
rs61825157	T	C	T	C	0.3558	-0.0100503	0.06717	NA	FALSE	FALSE	FALSE	ebi-a-GCST(	1	228299452	0.0605915	14267	0.868259	Systemic lup	Systemic lup	Systemic lup	TRUE	igd
rs72759354	C	G	C	G	0.1537	0.00995033	0.03086	NA	FALSE	TRUE	TRUE	ebi-a-GCST(	1	227518713	0.129093	14267	0.938561	Systemic lup	Systemic lup	Systemic lup	TRUE	igd
rs71467906	CCCTTG	GCCTTG	G	C	0.2457	0.0198026	0.01725	NA	TRUE	FALSE	FALSE	ebi-a-GCST(	12	113835773	0.0967546	14267	0.837831	Systemic lup	Systemic lup	Systemic lup	TRUE	igd
rs7294372	CTGGAA	TTGGAA	G	A	-0.2053	0.0487902	0.0138	NA	TRUE	FALSE	FALSE	ebi-a-GCST(	12	114637423	0.0899232	14267	0.587422	Systemic lup	Systemic lup	Systemic lup	TRUE	igd

SNP	proxy.outc	target_snp.o	proxy_snp.o	target_a1.ou	target_a2.ou	proxy_a1.ou	proxy_a2.ou	id.exposure	exposure	chr.exposure	pos.exposur	se.exposure	pval.exposur	samplesize.e	mr_keep.exp	pval_origin.e	data_source.	action	mr_keep
rs10305656	NA	NA	NA	NA	NA	NA	NA	15370_5_BC	BOLA1	chr1	150868044	0.028046	5.88E-36	35362	TRUE	reported	textfile	2	FALSE
rs60162170	NA	NA	NA	NA	NA	NA	NA	15370_5_BC	BOLA1	chr1	150538668	0.011965	7.08E-11	35361	TRUE	reported	textfile	2	TRUE
rs10814111	NA	NA	NA	NA	NA	NA	NA	16583_8_NU	NUDT2	chr9	34457854	0.014889	1.97E-34	35350	TRUE	reported	textfile	2	TRUE
rs111577519	NA	NA	NA	NA	NA	NA	NA	16583_8_NU	NUDT2	chr9	33499936	0.011523	2.51E-10	35348	TRUE	reported	textfile	2	TRUE
rs7025269	NA	NA	NA	NA	NA	NA	NA	16583_8_NU	NUDT2	chr9	34336268	0.009776	1.00E-200	35350	TRUE	reported	textfile	2	TRUE
rs10853721	NA	NA	NA	NA	NA	NA	NA	12662_82_E	ECH1	chr19	38821856	0.011774	2.32E-11	35366	TRUE	reported	textfile	2	TRUE
rs4802890	NA	NA	NA	NA	NA	NA	NA	12662_82_E	ECH1	chr19	38813762	0.013784	1.00E-200	35366	TRUE	reported	textfile	2	TRUE
rs73544031	NA	NA	NA	NA	NA	NA	NA	12662_82_E	ECH1	chr19	39280627	0.035106	1.10E-08	35359	TRUE	reported	textfile	2	TRUE
rs10939687	NA	NA	NA	NA	NA	NA	NA	13959_7_LA	LAP3	chr4	16732475	0.021086	1.27E-11	35359	TRUE	reported	textfile	2	TRUE
rs141974152	NA	NA	NA	NA	NA	NA	NA	13959_7_LA	LAP3	chr4	18162078	0.037901	4.07E-32	35354	TRUE	reported	textfile	2	TRUE
rs10939687	NA	NA	NA	NA	NA	NA	NA	15610_72_L	LAP3	chr4	16732475	0.02105	2.25E-11	35359	TRUE	reported	textfile	2	TRUE
rs141974152	NA	NA	NA	NA	NA	NA	NA	15610_72_L	LAP3	chr4	18162078	0.037831	7.73E-31	35354	TRUE	reported	textfile	2	TRUE
rs10973326	NA	NA	NA	NA	NA	NA	NA	18295_102_	GRHPR	chr9	37419500	0.008531	1.00E-200	35353	TRUE	reported	textfile	2	TRUE
rs72737776	NA	NA	NA	NA	NA	NA	NA	18295_102_	GRHPR	chr9	37279581	0.019757	1.44E-15	35355	TRUE	reported	textfile	2	TRUE
rs7874199	NA	NA	NA	NA	NA	NA	NA	18295_102_	GRHPR	chr9	37698874	0.009622	2.19E-11	35354	TRUE	reported	textfile	2	TRUE
rs11025480	NA	NA	NA	NA	NA	NA	NA	10630_5_HT	HTATIP2	chr11	20304545	0.009983	1.00E-200	35370	TRUE	reported	textfile	2	TRUE
rs1356981	NA	NA	NA	NA	NA	NA	NA	10630_5_HT	HTATIP2	chr11	20221745	0.008314	3.94E-25	35369	TRUE	reported	textfile	2	TRUE
rs1715284	NA	NA	NA	NA	NA	NA	NA	10630_5_HT	HTATIP2	chr11	20665751	0.010405	3.86E-35	35366	TRUE	reported	textfile	2	TRUE
rs2003174	NA	NA	NA	NA	NA	NA	NA	10630_5_HT	HTATIP2	chr11	20970593	0.01403	3.01E-14	35360	TRUE	reported	textfile	2	FALSE
rs3758807	NA	NA	NA	NA	NA	NA	NA	10630_5_HT	HTATIP2	chr11	20616985	0.008676	1.71E-47	35371	TRUE	reported	textfile	2	TRUE
rs79225249	NA	NA	NA	NA	NA	NA	NA	10630_5_HT	HTATIP2	chr11	20024031	0.018183	6.25E-16	35367	TRUE	reported	textfile	2	FALSE
rs11055175	NA	NA	NA	NA	NA	NA	NA	18225_13_H	HEBP1	chr12	12968844	0.020156	1.00E-200	35374	TRUE	reported	textfile	2	TRUE
rs12368093	NA	NA	NA	NA	NA	NA	NA	18225_13_H	HEBP1	chr12	13250139	0.009765	5.20E-13	35376	TRUE	reported	textfile	2	TRUE
rs1379509	NA	NA	NA	NA	NA	NA	NA	18225_13_H	HEBP1	chr12	13381617	0.012944	1.35E-27	35376	TRUE	reported	textfile	2	TRUE
rs73068707	NA	NA	NA	NA	NA	NA	NA	18225_13_H	HEBP1	chr12	12936946	0.013324	1.59E-42	35369	TRUE	reported	textfile	2	FALSE
rs1122818	NA	NA	NA	NA	NA	NA	NA	17782_23_A	ACAA1	chr3	39099605	0.01065	7.65E-12	35357	TRUE	reported	textfile	2	TRUE
rs2685096	NA	NA	NA	NA	NA	NA	NA	17782_23_A	ACAA1	chr3	37772103	0.008451	1.04E-21	35357	TRUE	reported	textfile	2	TRUE
rs7625290	NA	NA	NA	NA	NA	NA	NA	17782_23_A	ACAA1	chr3	38136799	0.019491	6.10E-20	35357	TRUE	reported	textfile	2	TRUE
rs115538711	NA	NA	NA	NA	NA	NA	NA	12859_33_E	ECI2	chr6	4400711	0.025725	4.80E-09	35374	TRUE	reported	textfile	2	FALSE
rs72845845	NA	NA	NA	NA	NA	NA	NA	12859_33_E	ECI2	chr6	3959999	0.015851	1.53E-15	35374	TRUE	reported	textfile	2	FALSE
rs115777975	NA	NA	NA	NA	NA	NA	NA	18215_5_TH	THG1L	chr5	157983348	0.027957	3.18E-16	35359	TRUE	reported	textfile	2	TRUE
rs35323270	NA	NA	NA	NA	NA	NA	NA	18215_5_TH	THG1L	chr5	158104312	0.008403	1.31E-10	35366	TRUE	reported	textfile	2	TRUE
rs62387542	TRUE	rs62387542	rs61291323	C	A	C	A	18215_5_TH	THG1L	chr5	157425743	0.009172	5.42E-25	35354	TRUE	reported	textfile	2	FALSE
rs7704907	NA	NA	NA	NA	NA	NA	NA	18215_5_TH	THG1L	chr5	157682236	0.00798	1.00E-200	35355	TRUE	reported	textfile	2	TRUE
rs11580071	NA	NA	NA	NA	NA	NA	NA	19277_4_TS	TSTD1	chr1	160982042	0.012805	1.00E-200	35364	TRUE	reported	textfile	2	TRUE
rs2255847	NA	NA	NA	NA	NA	NA	NA	19277_4_TS	TSTD1	chr1	160957147	0.010743	7.45E-09	35363	TRUE	reported	textfile	2	TRUE
rs78714351	NA	NA	NA	NA	NA	NA	NA	19277_4_TS	TSTD1	chr1	160835328	0.027747	4.24E-13	35365	TRUE	reported	textfile	2	TRUE
rs116161634	NA	NA	NA	NA	NA	NA	NA	5742_14_AC	ACP6	chr1	147616333	0.033252	7.14E-74	35359	TRUE	reported	textfile	2	TRUE
rs116509906	NA	NA	NA	NA	NA	NA	NA	5742_14_AC	ACP6	chr1	147851279	0.020793	1.91E-44	35360	TRUE	reported	textfile	2	FALSE
rs143333817	NA	NA	NA	NA	NA	NA	NA	5742_14_AC	ACP6	chr1	147555803	0.024642	1.00E-200	35358	TRUE	reported	textfile	2	TRUE
rs202051594	TRUE	rs202051594	rs61841174	T	C	C	T	5742_14_AC	ACP6	chr1	147327330	0.032202	3.10E-125	35357	TRUE	reported	textfile	2	TRUE
rs2985722	NA	NA	NA	NA	NA	NA	NA	5742_14_AC	ACP6	chr1	147061747	0.009677	1.64E-23	35358	TRUE	reported	textfile	2	TRUE
rs59326551	NA	NA	NA	NA	NA	NA	NA	5742_14_AC	ACP6	chr1	147831137	0.024764	5.70E-43	35360	TRUE	reported	textfile	2	FALSE
rs76390540	NA	NA	NA	NA	NA	NA	NA	5742_14_AC	ACP6	chr1	147678651	0.026998	1.87E-124	35358	TRUE	reported	textfile	2	TRUE
rs791283	TRUE	rs791283	rs115393172	C	T	A	G	5742_14_AC	ACP6	chr1	147774182	0.02192	2.13E-27	35360	TRUE	reported	textfile	2	TRUE
rs1165681	NA	NA	NA	NA	NA	NA	NA	9126_171_N	NT5DC3	chr12	103931333	0.008291	8.96E-10	35361	TRUE	reported	textfile	2	TRUE
rs954921	NA	NA	NA	NA	NA	NA	NA	9126_171_N	NT5DC3	chr12	103779852	0.00795	2.00E-98	35362	TRUE	reported	textfile	2	TRUE
rs117789622	NA	NA	NA	NA	NA	NA	NA	14091_42_C	CBR3	chr21	36224937	0.03703	5.73E-15	35384	TRUE	reported	textfile	2	TRUE
rs138308942	NA	NA	NA	NA	NA	NA	NA	14091_42_C	CBR3	chr21	35378941	0.021046	1.64E-09	35379	TRUE	reported	textfile	2	FALSE
rs17814116	NA	NA	NA	NA	NA	NA	NA	14091_42_C	CBR3	chr21	36339787	0.019525	8.20E-15	35385	TRUE	reported	textfile	2	TRUE
rs218634	NA	NA	NA	NA	NA	NA	NA	14091_42_C	CBR3	chr21	36422623	0.012147	1.44E-09	35383	TRUE	reported	textfile	2	TRUE
rs73202262	NA	NA	NA	NA	NA	NA	NA	14091_42_C	CBR3	chr21	36249629	0.023112	1.83E-23	35384	TRUE	reported	textfile	2	TRUE
rs117799316	NA	NA	NA	NA	NA	NA	NA	16809_1_NM	NME4	chr16	441484	0.022388	2.24E-08	35319	TRUE	reported	textfile	2	TRUE
rs3848364	NA	NA	NA	NA	NA	NA	NA	16809_1_NM	NME4	chr16	322635	0.008472	3.26E-13	35319	TRUE	reported	textfile	2	TRUE
rs117918535	NA	NA	NA	NA	NA	NA	NA	13929_27_C	CROT	chr7	86708970	0.014542	2.88E-17	35350	TRUE	reported	textfile	2	FALSE
rs193163017	NA	NA	NA	NA	NA	NA	NA	13929_27_C	CROT	chr7	88113664	0.016324	1.05E-25	35352	TRUE	reported	textfile	2	TRUE
rs7810499	NA	NA	NA	NA	NA	NA	NA	13929_27_C	CROT	chr7	87657130	0.016541	1.47E-10	35354	TRUE	reported	textfile	2	TRUE
rs809066	NA	NA	NA	NA	NA	NA	NA	13929_27_C	CROT	chr7	87358339	0.010636	1.00E-200	35347	TRUE	reported	textfile	2	TRUE
rs12129832	NA	NA	NA	NA	NA	NA	NA	12486_8_GL	GLRX2	chr1	193008121	0.019983	8.13E-21	35336	TRUE	reported	textfile	2	TRUE
rs72743118	NA	NA	NA	NA	NA	NA	NA	12486_8_GL	GLRX2	chr1	193991480	0.030498	5.94E-26	35334	TRUE	reported	textfile	2	TRUE
rs12403088	NA	NA	NA	NA	NA	NA	NA	13983_27_C	CRYZ	chr1	73770809	0.008458	8.33E-18	35356	TRUE	reported	textfile	2	TRUE
rs13375787	NA	NA	NA	NA	NA	NA	NA	13983_27_C	CRYZ	chr1	74713029	0.014778	1.00E-200	35354	TRUE	reported	textfile	2	TRUE
rs277392	NA	NA	NA	NA	NA	NA	NA	13983_27_C	CRYZ	chr1	74817278	0.008447	1.00E-200	35353	TRUE	reported	textfile	2	TRUE
rs72675345	NA	NA	NA	NA	NA	NA	NA	13983_27_C	CRYZ	chr1	74515676	0.034606	2.22E-36	35354	TRUE	reported	textfile	2	TRUE
rs76296629	NA	NA	NA	NA	NA	NA	NA	13983_27_C	CRYZ	chr1	74591159	0.015085	1.00E-200	35354	TRUE	reported	textfile	2	TRUE
rs12464143	TRUE	rs12464143	rs12693591	A	T	A	C	12396_19_H	HIBCH	chr2	190991698	0.017635	4.08E-13	35361	TRUE	reported	textfile	2	FALSE
rs62184386	NA	NA	NA	NA	NA	NA	NA	12396_19_H	HIBCH	chr2	190220892	0.007875	1.00E-200	35369	TRUE	reported	textfile	2	TRUE
rs75949659	NA	NA	NA	NA	NA	NA	NA	12396_19_H	HIBCH	chr2	189399596	0.012272	3.86E-13	35359	TRUE	reported	textfile	2	TRUE
rs785223	NA	NA	NA	NA	NA	NA	NA	12396_19_H	HIBCH	chr2	190137863	0.010015	5.49E-09	35367	TRUE	reported	textfile	2	TRUE
rs12881757	NA	NA	NA	NA	NA	NA	NA	16872_248_	GSTZ1	chr14	77652569	0.012085	1.00E-200	35354	TRUE	reported	textfile	2	FALSE
rs149721633	NA	NA	NA	NA	NA	NA	NA	16872_248_	GSTZ1	chr14	77334895	0.026471	2.05E-66	35356	TRUE	reported	textfile	2	TRUE
rs186433582	NA	NA	NA	NA	NA	NA	NA	16872_248_	GSTZ1	chr14	77012702	0.022735	1.00E-200	35357	TRUE	reported	textfile	2	TRUE
rs4467006	NA	NA	NA	NA	NA	NA	NA	16872_248_	GSTZ1	chr14	77218327	0.008938	1.00E-200	35353	TRUE	reported	textfile	2	FALSE
rs45608147	NA	NA	NA	NA	NA	NA	NA	16872_248_	GSTZ1	chr14	77269419	0.028589	1.00E-200	35355	TRUE	reported	textfile	2	FALSE
rs56198483	NA	NA	NA	NA	NA	NA	NA	16872_248_	GSTZ1	chr14	77362087	0.037806	1.22E-107	35356	TRUE	reported	textfile	2	TRUE
rs755072	NA	NA	NA	NA	NA	NA	NA	16872_248_	GSTZ1	chr14	77305402	0.026036	1.00E-200	35355	TRUE	reported	textfile	2	TRUE
rs78371502	NA	NA	NA	NA	NA	NA	NA	16872_248_	GSTZ1	chr14	77485110	0.014498	4.50E-133	35354	TRUE	reported	textfile	2	TRUE
rs79607813	NA	NA	NA	NA	NA	NA	NA	16872_248_	GSTZ1	chr14	77097333	0.031313	9.76E-14	35357	TRUE	reported	textfile	2	TRUE
rs1344544	NA	NA	NA	NA	NA	NA	NA	17783_9_MM	MMAB	chr12	109644266	0.008504	1.46E-57	35370	TRUE	reported	textfile	2	TRUE
rs16939972	NA	NA	NA	NA	NA	NA	NA	17783_9_MM	MMAB	chr12	109130992	0.008238	1.20E-30	35372	TRUE	reported	textfile	2	TRUE
rs190825201	NA	NA	NA	NA	NA	NA	NA	17783_9_MM	MMAB	chr12	109668624	0.028345	6.09E-28	35366	TRUE	reported	textfile	2	TRUE
rs4766598	TRUE	rs4766598	rs35393419	G	A	T	C	17783_9_MM	MMAB	chr12	109415501	0.009401	1.00E-200	35372	TRUE	reported	textfile	2	TRUE
rs78925260	NA	NA	NA	NA	NA	NA	NA	17783_9_MM	MMAB	chr12	110314410	0.024496	1.09E-14	35370	TRUE	reported	textfile	2	TRUE
rs1380642	NA	NA	NA	NA	NA	NA	NA	14107_1_MT	MTHFS	chr15	79804529	0.009578	1.00E-200	35358	TRUE	reported	textfile	2	TRUE
rs62026657	NA	NA	NA	NA	NA	NA	NA	14107_1_MT	MTHFS	chr15	79818657	0.016509	2.31E-99	35358	TRUE	reported	textfile	2	TRUE
rs72736560	NA	NA	NA	NA	NA	NA	NA	14107_1_MT	MTHFS	chr15	79948498	0.025268	1.83E-21	35358	TRUE	reported	textfile	2	TRUE
rs80102939	NA	NA	NA	NA	NA	NA	NA	14107_1_MT	MTHFS	chr15	80383266	0.012123	2.46E-09	35356	TRUE	reported	textfile	2	TRUE
rs144303868	NA	NA	NA	NA	NA	NA	NA	19504_22_D	DTYMK	chr2	241680143	0.017131	3.05E-20	35117	TRUE	reported	textfile	2	FALSE
rs1675513	TRUE	rs1675513	rs1729988	G	A	T	C	18241_18_C	CPOX	chr3	98589854	0.011719	5.62E-58	35330	TRUE	reported	textfile	2	TRUE
rs1841542	NA	NA	NA	NA	NA	NA	NA	18241_18_C	CPOX	chr3	98629898	0.009388	2.49E-17	35330	TRUE	reported	textfile	2	TRUE
rs17714829	NA	NA	NA	NA	NA	NA	NA	11117_2_SP	SPATA20	chr17	50375120	0.015247	1.85E-16	35357	TRUE	reported	textfile	2	TRUE
rs77405995	NA	NA	NA	NA	NA	NA	NA	11117_2_SP	SPATA20	chr17	50284012	0.018545	4.41E-11	35350	TRUE	reported	textfile	2	TRUE
rs9890200	NA	NA	NA	NA	NA	NA	NA	11117_2_SP	SPATA20	chr17	50547162	0.007116	1.00E-200	35353	TRUE	reported	textfile	2	TRUE
rs1861123	NA	NA	NA	NA	NA	NA	NA	11257_1_QD	QDPR	chr4	18284852	0.011462	1.16E-08	35345	TRUE	reported	textfile	2	TRUE
rs3822233	NA	NA	NA	NA	NA	NA	NA	11257_1_QD	QDPR	chr4	17509724	0.007528	1.00E-200	35346	TRUE	reported	textfile	2	TRUE
rs75218392	NA	NA	NA	NA	NA	NA	NA	11257_1_QD	QDPR	chr4	17400926	0.019459	6.80E-25	35346	TRUE	reported	textfile	2	TRUE
rs2746626	TRUE	rs2746626	rs2611084	C	G	A	G	3488_64_CA	CAT	chr11	34217843	0.010534	2.51E-08	35365	TRUE	reported	textfile	2	FALSE
rs2755171	NA	NA	NA	NA	NA	NA	NA	3488_64_CA	CAT	chr11	34328076	0.009122	3.36E-23	35368	TRUE	reported	textfile	2	TRUE
rs769218	NA	NA	NA	NA	NA	NA	NA	3488_64_CA	CAT	chr11	34449132	0.009637	2.99E-107	35374	TRUE	reported	textfile	2	TRUE
rs35022330	TRUE	rs35022330	rs12476089	G	A	C	T	5227_60_PD	PDK1	chr2	172548130	0.009759	3.27E-97	35362	TRUE	reported	textfile	2	FALSE
rs35783685	NA	NA	NA	NA	NA	NA	NA	5227_60_PD	PDK1	chr2	172458199	0.009363	2.69E-09	35366	TRUE	reported	textfile	2	TRUE
rs4464946	TRUE	rs4464946	rs2953516	A	G	T	C	7096_30_RM	RMDN1	chr8	86314030	0.008889	1.00E-200	35349	TRUE	reported	textfile	2	TRUE
rs4961042	NA	NA	NA	NA	NA	NA	NA	7096_30_RM	RMDN1	chr8	86044356	0.008165	1.16E-31	35348	TRUE	reported	textfile	2	TRUE
rs75979940	NA	NA	NA	NA	NA	NA	NA	7096_30_RM	RMDN1	chr8	86917707	0.017976	7.78E-12	35353	TRUE	reported	textfile	2	TRUE
rs79117756	NA	NA	NA	NA	NA	NA	NA	7096_30_RM	RMDN1	chr8	85586830	0.017963	8.12E-13	35349	TRUE	reported	textfile	2	TRUE
rs4733505	NA	NA	NA	NA	NA	NA	NA	19273_3_GS	GSR	chr8	30695913	0.008578	1.26E-39	35358	TRUE	reported	textfile	2	TRUE
rs61825157	NA	NA	NA	NA	NA	NA	NA	17325_10_G	GUK1	chr1	228111751	0.016093	2.59E-108	35358	TRUE	reported	textfile	2	TRUE
rs72759354	NA	NA	NA	NA	NA	NA	NA	17325_10_G	GUK1	chr1	227331012	0.023569	6.97E-11	35360	TRUE	reported	textfile	2	FALSE
rs71467906	NA	NA	NA	NA	NA	NA	NA	17777_31_S	SDSL	chr12	113397968	0.031615	7.74E-15	35366	TRUE	reported	textfile	2	FALSE
rs7294372	NA	NA	NA	NA	NA	NA	NA	17777_31_S	SDSL	chr12	114199618	0.034862	3.89E-09	35373	TRUE	reported	textfile	2	FALSE

Table S13. The all result of two-sample Mendelian randomization analysis for association between mitochondrial gene encoded protein and systemic lupus erythematosus

id.exposure	id.outcome	outcome	exposure	method	nsnp	b	se	pval	pval1	OR	or_uci95	or_lci95
10630_5_HT	ebi-a-GCST(	Systemic lup	HTATIP2	MR Egger	4	0.203907071	0.174614693	0.363283528	0.363	1.226	1.727	0.871
10630_5_HT	ebi-a-GCST(	Systemic lup	HTATIP2	Weighted me	4	0.213386866	0.087332008	0.014549676	0.015	1.238	1.469	1.043
10630_5_HT	ebi-a-GCST(	Systemic lup	HTATIP2	Inverse varia	4	0.21232941	0.084284991	0.011762713	0.012	1.237	1.459	1.048
10630_5_HT	ebi-a-GCST(	Systemic lup	HTATIP2	Simple mode	4	0.233832093	0.145028549	0.205287211	0.205	1.263	1.679	0.951
10630_5_HT	ebi-a-GCST(	Systemic lup	HTATIP2	Weighted mo	4	0.211700529	0.094131018	0.110040071	0.11	1.236	1.486	1.028
11117_2_SF	ebi-a-GCST(	Systemic lup	SPATA20	MR Egger	3	0.448428923	0.362133916	0.432477847	0.432	1.566	3.184	0.77
11117_2_SF	ebi-a-GCST(	Systemic lup	SPATA20	Weighted me	3	0.24886795	0.105949163	0.018827073	0.019	1.283	1.579	1.042
11117_2_SF	ebi-a-GCST(	Systemic lup	SPATA20	Inverse varia	3	0.239967606	0.099763825	0.016156677	0.016	1.271	1.546	1.045
11117_2_SF	ebi-a-GCST(	Systemic lup	SPATA20	Simple mode	3	0.381822777	0.204621922	0.203027843	0.203	1.465	2.188	0.981
11117_2_SF	ebi-a-GCST(	Systemic lup	SPATA20	Weighted mo	3	0.26477406	0.107209908	0.132206522	0.132	1.303	1.608	1.056
11257_1_QI	ebi-a-GCST(	Systemic lup	QDPR	MR Egger	3	0.217340973	0.161779717	0.407360962	0.407	1.243	1.706	0.905
11257_1_QI	ebi-a-GCST(	Systemic lup	QDPR	Weighted me	3	0.078114349	0.06779669	0.249244942	0.249	1.081	1.235	0.947
11257_1_QI	ebi-a-GCST(	Systemic lup	QDPR	Inverse varia	3	0.071346918	0.0675002	0.290516951	0.291	1.074	1.226	0.941
11257_1_QI	ebi-a-GCST(	Systemic lup	QDPR	Simple mode	3	0.088491505	0.138736719	0.588862747	0.589	1.093	1.434	0.832
11257_1_QI	ebi-a-GCST(	Systemic lup	QDPR	Weighted mo	3	0.078726078	0.066391555	0.357492739	0.357	1.082	1.232	0.95
12396_19_H	ebi-a-GCST(	Systemic lup	HIBCH	MR Egger	3	-0.287130363	0.221370468	0.418126021	0.418	0.75	1.158	0.486
12396_19_H	ebi-a-GCST(	Systemic lup	HIBCH	Weighted me	3	-0.248653985	0.123609524	0.04426121	0.044	0.78	0.994	0.612
12396_19_H	ebi-a-GCST(	Systemic lup	HIBCH	Inverse varia	3	-0.247447313	0.119303393	0.038069911	0.038	0.781	0.986	0.618
12396_19_H	ebi-a-GCST(	Systemic lup	HIBCH	Simple mode	3	-0.156344979	0.20984728	0.533901469	0.534	0.855	1.29	0.567
12396_19_H	ebi-a-GCST(	Systemic lup	HIBCH	Weighted mo	3	-0.254312811	0.120410929	0.169074714	0.169	0.775	0.982	0.612
12486_8_GL	ebi-a-GCST(	Systemic lup	GLRX2	Inverse varia	2	0.145272075	0.288984371	0.615176105	0.615	1.156	2.037	0.656
12662_82_E	ebi-a-GCST(	Systemic lup	ECH1	MR Egger	3	-0.003007785	0.18093756	0.989418232	0.989	0.997	1.421	0.699
12662_82_E	ebi-a-GCST(	Systemic lup	ECH1	Weighted me	3	0.092441348	0.109065343	0.396673222	0.397	1.097	1.358	0.886
12662_82_E	ebi-a-GCST(	Systemic lup	ECH1	Inverse varia	3	0.105250131	0.106456918	0.322827522	0.323	1.111	1.369	0.902
12662_82_E	ebi-a-GCST(	Systemic lup	ECH1	Simple mode	3	0.117414695	0.209091705	0.630955455	0.631	1.125	1.694	0.746
12662_82_E	ebi-a-GCST(	Systemic lup	ECH1	Weighted mo	3	0.088713689	0.110775784	0.507242487	0.507	1.093	1.358	0.879
13929_27_C	ebi-a-GCST(	Systemic lup	CROT	MR Egger	3	0.118449664	0.302974236	0.762740858	0.763	1.126	2.039	0.622
13929_27_C	ebi-a-GCST(	Systemic lup	CROT	Weighted me	3	0.039795654	0.08764676	0.649795775	0.65	1.041	1.236	0.876
13929_27_C	ebi-a-GCST(	Systemic lup	CROT	Inverse varia	3	0.038640184	0.086725492	0.655925329	0.656	1.039	1.232	0.877
13929_27_C	ebi-a-GCST(	Systemic lup	CROT	Simple mode	3	0.127420453	0.182923818	0.558138099	0.558	1.136	1.626	0.794
13929_27_C	ebi-a-GCST(	Systemic lup	CROT	Weighted mo	3	0.065446218	0.091920423	0.550321906	0.55	1.068	1.278	0.892
13959_7_LA	ebi-a-GCST(	Systemic lup	LAP3	Inverse varia	2	-0.386021961	0.274152295	0.159114297	0.159	0.68	1.163	0.397
13983_27_C	ebi-a-GCST(	Systemic lup	CRYZ	MR Egger	5	0.051292724	0.144036335	0.745329307	0.745	1.053	1.396	0.794
13983_27_C	ebi-a-GCST(	Systemic lup	CRYZ	Weighted me	5	0.023760088	0.080114111	0.766788657	0.767	1.024	1.198	0.875
13983_27_C	ebi-a-GCST(	Systemic lup	CRYZ	Inverse varia	5	0.049857372	0.058356544	0.392906743	0.393	1.051	1.178	0.938
13983_27_C	ebi-a-GCST(	Systemic lup	CRYZ	Simple mode	5	-0.0343978	0.12700204	0.799912436	0.8	0.966	1.239	0.753
13983_27_C	ebi-a-GCST(	Systemic lup	CRYZ	Weighted mo	5	0.128670055	0.086852065	0.212601043	0.213	1.137	1.348	0.959
14091_42_C	ebi-a-GCST(	Systemic lup	CBR3	MR Egger	4	-0.090451453	0.705695725	0.909737646	0.91	0.914	3.643	0.229
14091_42_C	ebi-a-GCST(	Systemic lup	CBR3	Weighted me	4	0.825677693	0.334390256	0.013541407	0.014	2.283	4.398	1.186
14091_42_C	ebi-a-GCST(	Systemic lup	CBR3	Inverse varia	4	0.471750022	0.247771301	0.05691362	0.057	1.603	2.605	0.986
14091_42_C	ebi-a-GCST(	Systemic lup	CBR3	Simple mode	4	0.866621967	0.493086624	0.177073161	0.177	2.379	6.253	0.905
14091_42_C	ebi-a-GCST(	Systemic lup	CBR3	Weighted mo	4	0.866621967	0.617483776	0.255075118	0.255	2.379	7.98	0.709
15610_72_L	ebi-a-GCST(	Systemic lup	LAP3	Inverse varia	2	-0.394043708	0.279628872	0.158785868	0.159	0.674	1.167	0.39
16583_8_NU	ebi-a-GCST(	Systemic lup	NUDT2	MR Egger	3	0.234213026	0.246237441	0.515929598	0.516	1.264	2.048	0.78
16583_8_NU	ebi-a-GCST(	Systemic lup	NUDT2	Weighted me	3	-0.064374827	0.105576688	0.542030933	0.542	0.938	1.153	0.762
16583_8_NU	ebi-a-GCST(	Systemic lup	NUDT2	Inverse varia	3	-0.097851704	0.108089226	0.365313691	0.365	0.907	1.121	0.734
16583_8_NU	ebi-a-GCST(	Systemic lup	NUDT2	Simple mode	3	-0.060031445	0.141325126	0.712334404	0.712	0.942	1.242	0.714
16583_8_NU	ebi-a-GCST(	Systemic lup	NUDT2	Weighted mo	3	-0.064220893	0.108340803	0.613434128	0.613	0.938	1.16	0.758
16809_1_NM	ebi-a-GCST(	Systemic lup	NME4	Inverse varia	2	0.78196078	0.416553077	0.060488272	0.06	2.186	4.945	0.966
16872_248_	ebi-a-GCST(	Systemic lup	GSTZ1	MR Egger	6	-0.035029843	0.174484285	0.850679486	0.851	0.966	1.359	0.686
16872_248_	ebi-a-GCST(	Systemic lup	GSTZ1	Weighted me	6	-0.004902429	0.086085525	0.954586332	0.955	0.995	1.178	0.841
16872_248_	ebi-a-GCST(	Systemic lup	GSTZ1	Inverse varia	6	0.055763569	0.069932369	0.425222922	0.425	1.057	1.213	0.922
16872_248_	ebi-a-GCST(	Systemic lup	GSTZ1	Simple mode	6	0.003123352	0.112682707	0.97895927	0.979	1.003	1.251	0.804
16872_248_	ebi-a-GCST(	Systemic lup	GSTZ1	Weighted mo	6	-0.010236093	0.099847289	0.922330543	0.922	0.99	1.204	0.814
17782_23_A	ebi-a-GCST(	Systemic lup	ACAA1	MR Egger	3	0.128466922	0.631458534	0.872226839	0.872	1.137	3.92	0.33
17782_23_A	ebi-a-GCST(	Systemic lup	ACAA1	Weighted me	3	-0.049509418	0.280387161	0.859842078	0.86	0.952	1.649	0.549
17782_23_A	ebi-a-GCST(	Systemic lup	ACAA1	Inverse varia	3	-0.129862398	0.225107526	0.564013414	0.564	0.878	1.365	0.565
17782_23_A	ebi-a-GCST(	Systemic lup	ACAA1	Simple mode	3	0.026336105	0.349478402	0.946789113	0.947	1.027	2.037	0.518
17782_23_A	ebi-a-GCST(	Systemic lup	ACAA1	Weighted mo	3	0.001411407	0.323969788	0.996919433	0.997	1.001	1.89	0.531
17783_9_MI	ebi-a-GCST(	Systemic lup	MMAB	MR Egger	5	0.197778321	0.203974986	0.40375581	0.404	1.219	1.818	0.817
17783_9_MI	ebi-a-GCST(	Systemic lup	MMAB	Weighted me	5	-0.046488311	0.102458208	0.650023533	0.65	0.955	1.167	0.781
17783_9_MI	ebi-a-GCST(	Systemic lup	MMAB	Inverse varia	5	-0.104978486	0.093615718	0.262127544	0.262	0.9	1.082	0.749
17783_9_MI	ebi-a-GCST(	Systemic lup	MMAB	Simple mode	5	-0.094896064	0.154406759	0.572066973	0.572	0.909	1.231	0.672
17783_9_MI	ebi-a-GCST(	Systemic lup	MMAB	Weighted mo	5	-0.05717309	0.110758172	0.632940346	0.633	0.944	1.173	0.76
18215_5_TH	ebi-a-GCST(	Systemic lup	THG1L	MR Egger	3	-0.057993241	0.288070026	0.87352843	0.874	0.944	1.66	0.537
18215_5_TH	ebi-a-GCST(	Systemic lup	THG1L	Weighted me	3	-0.084570305	0.126503835	0.503801917	0.504	0.919	1.177	0.717
18215_5_TH	ebi-a-GCST(	Systemic lup	THG1L	Inverse varia	3	-0.090237063	0.125184113	0.471011172	0.471	0.914	1.168	0.715
18215_5_TH	ebi-a-GCST(	Systemic lup	THG1L	Simple mode	3	-0.15683504	0.255629326	0.602010655	0.602	0.855	1.411	0.518
18215_5_TH	ebi-a-GCST(	Systemic lup	THG1L	Weighted mo	3	-0.079486035	0.127275936	0.596035142	0.596	0.924	1.185	0.72
18225_13_H	ebi-a-GCST(	Systemic lup	HEBP1	MR Egger	3	-0.047700995	0.077662295	0.64934798	0.649	0.953	1.11	0.819
18225_13_H	ebi-a-GCST(	Systemic lup	HEBP1	Weighted me	3	-0.024222435	0.052495876	0.644500203	0.645	0.976	1.082	0.881
18225_13_H	ebi-a-GCST(	Systemic lup	HEBP1	Inverse varia	3	-0.015411294	0.05242098	0.768764808	0.769	0.985	1.091	0.889
18225_13_H	ebi-a-GCST(	Systemic lup	HEBP1	Simple mode	3	-0.045967172	0.100922737	0.69344166	0.693	0.955	1.164	0.784
18225_13_H	ebi-a-GCST(	Systemic lup	HEBP1	Weighted mo	3	-0.024260532	0.051575327	0.684384776	0.684	0.976	1.08	0.882
18295_102_	ebi-a-GCST(	Systemic lup	GRHPR	MR Egger	3	-0.169072375	0.232087273	0.599190873	0.599	0.844	1.331	0.536
18295_102_	ebi-a-GCST(	Systemic lup	GRHPR	Weighted me	3	-0.017209813	0.084697844	0.838985989	0.839	0.983	1.16	0.833
18295_102_	ebi-a-GCST(	Systemic lup	GRHPR	Inverse varia	3	-0.00741121	0.085807432	0.931172092	0.931	0.993	1.174	0.839
18295_102_	ebi-a-GCST(	Systemic lup	GRHPR	Simple mode	3	0.290663591	0.282972884	0.412330105	0.412	1.337	2.329	0.768
18295_102_	ebi-a-GCST(	Systemic lup	GRHPR	Weighted mo	3	-0.033507077	0.086521586	0.73588371	0.736	0.967	1.146	0.816
19277_4_TS	ebi-a-GCST(	Systemic lup	TSTD1	MR Egger	3	0.195820299	0.148485996	0.413024439	0.413	1.216	1.627	0.909
19277_4_TS	ebi-a-GCST(	Systemic lup	TSTD1	Weighted me	3	0.143423569	0.097553527	0.141506557	0.142	1.154	1.397	0.953
19277_4_TS	ebi-a-GCST(	Systemic lup	TSTD1	Inverse varia	3	0.136776538	0.094275588	0.146831083	0.147	1.147	1.379	0.953
19277_4_TS	ebi-a-GCST(	Systemic lup	TSTD1	Simple mode	3	0.191910178	0.157952094	0.348341246	0.348	1.212	1.651	0.889
19277_4_TS	ebi-a-GCST(	Systemic lup	TSTD1	Weighted mo	3	0.144495079	0.097027193	0.274867385	0.275	1.155	1.397	0.955
5742_14_AC	ebi-a-GCST(	Systemic lup	ACP6	MR Egger	6	0.079909278	0.106523328	0.494874403	0.495	1.083	1.335	0.879
5742_14_AC	ebi-a-GCST(	Systemic lup	ACP6	Weighted me	6	0.117857431	0.091462267	0.197540403	0.198	1.125	1.346	0.94
5742_14_AC	ebi-a-GCST(	Systemic lup	ACP6	Inverse varia	6	0.113373072	0.072268029	0.116697814	0.117	1.12	1.29	0.972
5742_14_AC	ebi-a-GCST(	Systemic lup	ACP6	Simple mode	6	0.304333153	0.135052369	0.073956739	0.074	1.356	1.767	1.04
5742_14_AC	ebi-a-GCST(	Systemic lup	ACP6	Weighted mo	6	0.163369429	0.112303426	0.205503054	0.206	1.177	1.467	0.945
7096_30_RM	ebi-a-GCST(	Systemic lup	RMDN1	MR Egger	4	0.056138508	0.298881496	0.868341229	0.868	1.058	1.9	0.589
7096_30_RM	ebi-a-GCST(	Systemic lup	RMDN1	Weighted me	4	-0.052534926	0.137994619	0.703423856	0.703	0.949	1.244	0.724
7096_30_RM	ebi-a-GCST(	Systemic lup	RMDN1	Inverse varia	4	-0.07846435	0.130534567	0.547773271	0.548	0.925	1.194	0.716
7096_30_RM	ebi-a-GCST(	Systemic lup	RMDN1	Simple mode	4	-0.084868449	0.196749708	0.695330502	0.695	0.919	1.351	0.625
7096_30_RM	ebi-a-GCST(	Systemic lup	RMDN1	Weighted mo	4	-0.04869298	0.14990918	0.766652462	0.767	0.952	1.278	0.71
9126_171_N	ebi-a-GCST(	Systemic lup	NT5DC3	Inverse varia	2	0.518594454	0.163294466	0.001494117	0.001	1.68	2.313	1.22
14107_1_MT	ebi-a-GCST(	Systemic lup	MTHFS	MR Egger	4	0.178387758	0.409184159	0.705409897	0.705	1.195	2.665	0.536
14107_1_MT	ebi-a-GCST(	Systemic lup	MTHFS	Weighted me	4	-0.073252916	0.111299913	0.510436682	0.51	0.929	1.156	0.747
14107_1_MT	ebi-a-GCST(	Systemic lup	MTHFS	Inverse varia	4	-0.101034775	0.175325696	0.564433328	0.564	0.904	1.275	0.641
14107_1_MT	ebi-a-GCST(	Systemic lup	MTHFS	Simple mode	4	-0.631583103	0.333787279	0.154822153	0.155	0.532	1.023	0.276
14107_1_MT	ebi-a-GCST(	Systemic lup	MTHFS	Weighted mo	4	-0.082519358	0.126488448	0.560681436	0.561	0.921	1.18	0.719
18241_18_C	ebi-a-GCST(	Systemic lup	CPOX	Inverse varia	2	-0.075546766	0.399456332	0.849995657	0.85	0.927	2.029	0.424
3488_64_CA	ebi-a-GCST(	Systemic lup	CAT	Inverse varia	2	-0.202811838	0.324314926	0.531738337	0.532	0.816	1.542	0.432
19273_3_GS	ebi-a-GCST(	Systemic lup	GSR	Wald ratio	1	0.361256637	0.241960177	0.135426268	0.135	1.435	2.306	0.893

Table S14. Results of heterogeneity and horizontal pleiotropy tests for positive exposures of TSMR analysis between pQTLs an

id.exposure	exposure	method	Q	Q df	Q pval	egger intercep	se	pval
10630_5_HTA	HTATIP2	MR Egger	0.035	2.000	0.983	0.002	0.036	0.961
10630_5_HTA	HTATIP2	IVW	0.038	3.000	0.998			
11117_2_SPA	SPATA20	MR Egger	1.470	1.000	0.225	-0.061	0.099	0.651
11117_2_SPA	SPATA20	IVW	2.018	2.000	0.365			
12396_19_HI	HIBCH	MR Egger	0.014	1.000	0.906	0.008	0.037	0.867
12396_19_HI	HIBCH	IVW	0.059	2.000	0.971			
9126_171_NT	NT5DC3	IVW	0.009	1.000	0.924	NA	NA	NA

Table S15.Summary data-level Mendelian randomization analysis for association between mitochondrial gene encoded protein and systemic lupus

probeID	ProbeChr	Gene	Probe bp	topSNP	topSNP c	topSNP bp	A1	A2	Freq	b GWAS	se GWAS	p GWAS
15370_5	1	BOLA1	149894344	rs134953	1	149899450	T	C	0.0864811	0.162519	0.0599199	0.006682362
13384_11	1	FH	241508655	rs667876	1	241527254	G	A	0.706759	-0.0725707	0.0345411	0.03564101
12396_19	2	HIBCH	190266964	rs291466	2	190319749	G	A	0.597416	0.10436	0.0307145	0.000679438
17782_23	3	ACAA1	38120186	rs360460	3	38045533	T	C	0.230616	0.0943107	0.0357004	0.008248405
18307_71	4	PPA2	105421572	rs469917	4	105474024	A	G	0.2167	-0.076961	0.0311546	0.01350019
5900_11	5	HINT1	131189926	rs112241	5	131141405	A	C	0.233598	0.0725707	0.0341442	0.03355212
17712_7	10	IDI1	1044136	rs488075	10	1039632	G	C	0.0854871	0.174353	0.0479157	0.000273962
9126_171	12	NT5DC3	103805844	rs954921	12	103779852	C	T	0.378728	-0.0861777	0.028143	0.002197657
18381_16	12	ALDH2	111792210	rs108499	12	111373800	A	G	0.207753	0.105361	0.0392371	0.007248197
15542_19	15	CKMT1A	43696054	rs284337	15	43696643	A	G	0.100398	-0.10436	0.0484092	0.03109997
18188_12	15	GATM	45381726	rs115385	15	45360505	T	C	0.276342	-0.0676586	0.029037	0.01980204
11117_2	17	SPATA20	50549455	rs807663	17	50548567	G	C	0.349901	0.0725707	0.0305669	0.01758895

erythematosus

b_eQTL	se_eQTL	p_eQTL	b_SMR	se_SMR	p_SMR	p_HEIDI	nsnp_HEIDI
0.9625	0.01049	0	0.168851	0.0622816	0.00670631	0.402756	20
0.1477	0.00871738	2.16E-64	-0.491339	0.235651	0.0370669	0.4116919	20
0.3279	0.00695	0	0.318268	0.0939129	0.000701565	0.01457062	20
-0.2278	0.0100247	2.60E-114	-0.414007	0.157774	0.008688988	0.4434649	16
0.1054	0.00907528	3.50E-31	-0.73018	0.302197	0.01568168	0.8070094	20
0.097	0.00973824	2.26E-23	0.748152	0.359926	0.03765193	0.9175707	20
-0.1062	0.0162371	6.13E-11	-1.64174	0.516307	0.001473861	0.8692747	20
0.1674	0.00795011	2.00E-98	-0.514801	0.169887	0.002443427	0.06024549	20
-0.0913	0.00914356	1.77E-23	-1.15401	0.445029	0.009511243	0.07616775	20
-0.202	0.0146905	5.07E-43	0.516634	0.242577	0.0331904	0.04918791	20
-0.1811	0.00922297	7.65E-86	0.373598	0.161462	0.02067606	0.3629702	20
-0.3215	0.007116	0	-0.225725	0.0952071	0.01774539	0.2287662	20

Table S16. Two eQTLs of mitochondria-related genes in skin tissue based on TSMR analysis.

GTEs	SNP	effect_allele	other_allele	effect_allele	other_allele	beta.exposu	beta.outcome	eaf.expos	eaf.outco	remove	palindrom	ambiguou
Skin_Sun	rs4582610	C	T	C	T	0.240828	-0.0725707	NA	NA	FALSE	FALSE	FALSE
Skin_Sun	rs9890200	C	A	C	A	-0.804667	-0.0833816	NA	NA	FALSE	FALSE	FALSE
Skin_Not_	rs12544112	A	C	A	C	-0.193162	0.0725707	NA	NA	FALSE	FALSE	FALSE
Skin_Not	rs9890200	C	A	C	A	-0.879427	-0.0833816	NA	NA	FALSE	FALSE	FALSE

id.outcom	chr	pos	se.outcome	samplesiz	pval.outcom	outcome	originalna	outcome.	mr keep.	data sou	V1	chr.expos	pos.exposu
ebi-a-GC:	8	10318343	0.0315218	14267	0.0213216	Systemic	Systemic	Systemic	TRUE	igd	4922	8	10318343
ebi-a-GC:	17	48624523	0.033068	14267	0.011685	Systemic	Systemic	Systemic	TRUE	igd	8451	17	48624523
ebi-a-GC:	8	10318815	0.0317293	14267	0.022185	Systemic	Systemic	Systemic	TRUE	igd	4501	8	10318815
ebi-a-GC:	17	48624523	0.033068	14267	0.011685	Systemic	Systemic	Systemic	TRUE	igd	7523	17	48624523

id.exposu	exposure	se.exposure	pval.exposu	action	SNP	inde	mr	keep
ENSG00(	MSRA	0.0314044	1.74E-14	2		1		TRUE
ENSG00(	SPATA2(	0.0184486	0	2		1		TRUE
ENSG00(	MSRA	0.0329696	4.66E-09	2		1		TRUE
ENSG00(	SPATA2(	0.0340204	2.43E-147	2		1		TRUE

Table S17.Summary data-level Mendelian randomization analysis for association between between the eQTLs of skin tissue (Skin_Sun, Skin_

probeID	ProbeChr	Gene	Probe_bp	topSNP	topSNP_(	topSNP_bp	A1	A2	Freq	b_GWAS	se_GWAS	p_GWAS
ENSG00(	8	MSRA	9911778	rs125441	8	10318815	A	C	0.550696	-0.0725707	0.0317293	0.02218503
ENSG00(	17	SPATA2(	48620419	rs989020	17	48624523	C	A	0.349901	0.0833816	0.033068	0.01168503
ENSG00(	8	MSRA	9911778	rs458261	8	10318343	C	T	0.449304	0.0725707	0.0315218	0.02132161
ENSG00(	17	SPATA2(	48620419	rs989020	17	48624523	C	A	0.349901	0.0833816	0.033068	0.01168503

_Not_Sun) and systemic lupus erythematosus

b_eQTL	se_eQTL	p_eQTL	b_SMR	se_SMR	p_SMR	p_HEIDI	nsnp_HE	SMR_OR	SMR_uci9	SMR_lci9	GTEx
-0.193162	0.0329696	4.66E-09	0.375699	0.176336	0.033	0.024	12	1.456	2.057	1.031	Skin_Sun
-0.879427	0.0340204	2.43E-147	-0.0948136	0.0377802	0.012	0.192	20	0.91	0.979	0.845	Skin_Sun
0.240828	0.0314044	1.74E-14	0.301338	0.136661	0.027	0.056	20	1.352	1.767	1.034	Skin_Not_Sun
-0.804667	0.0184486	0	-0.103622	0.0411639	0.012	0.272	20	0.902	0.977	0.832	Skin_Not_Sun
