# Supplementary material for: Association between mitochondria-related genes and systemic lupus erythematosus: Findings from Mendelian randomization study
Source: Medicine (Baltimore). 2025 Oct 31;104(44):e45301. doi: 10.1097/MD.0000000000045301 (PMC12582722; doi:10.1097/MD.0000000000045301)

Supplementary Fig 1: visualized Mendelian randomization analysis of mQTLs for mitochondria-related genes

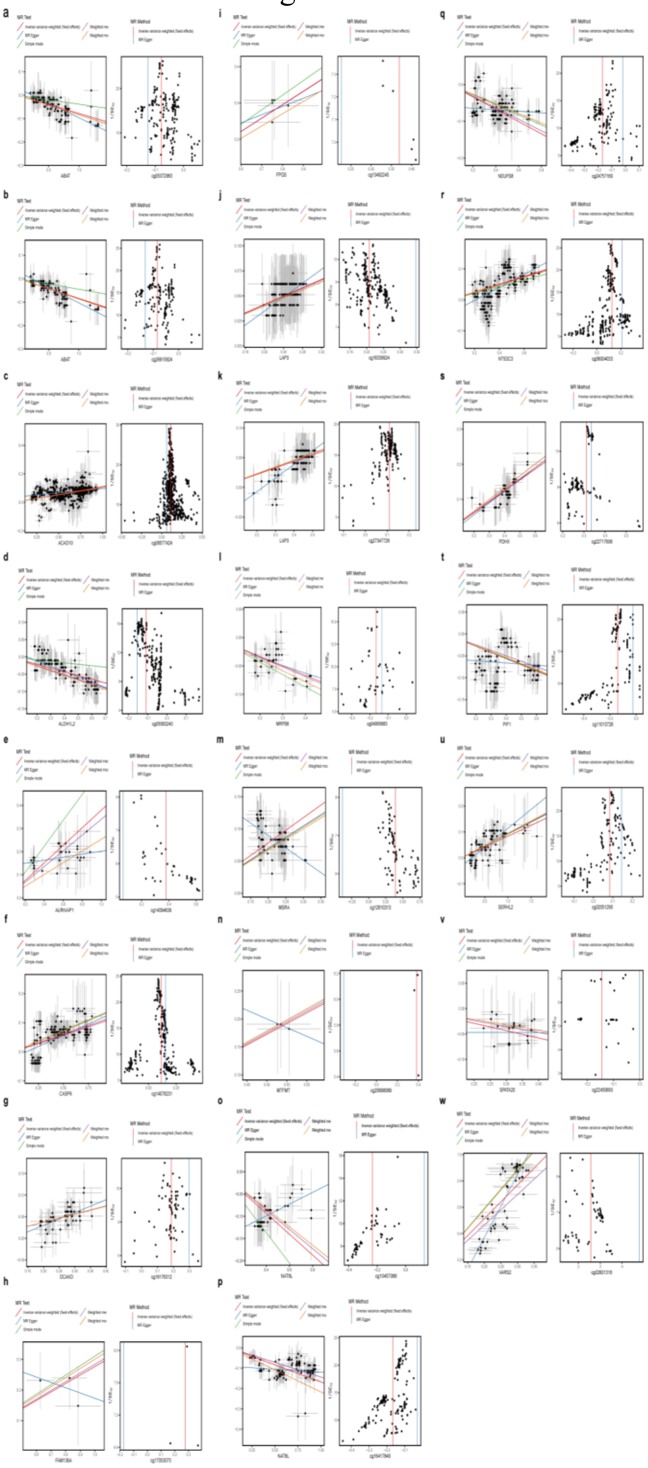

Supplementary Fig 2: Visualized Mendelian randomization analysis of the eQTLs of mitochondria-related genes

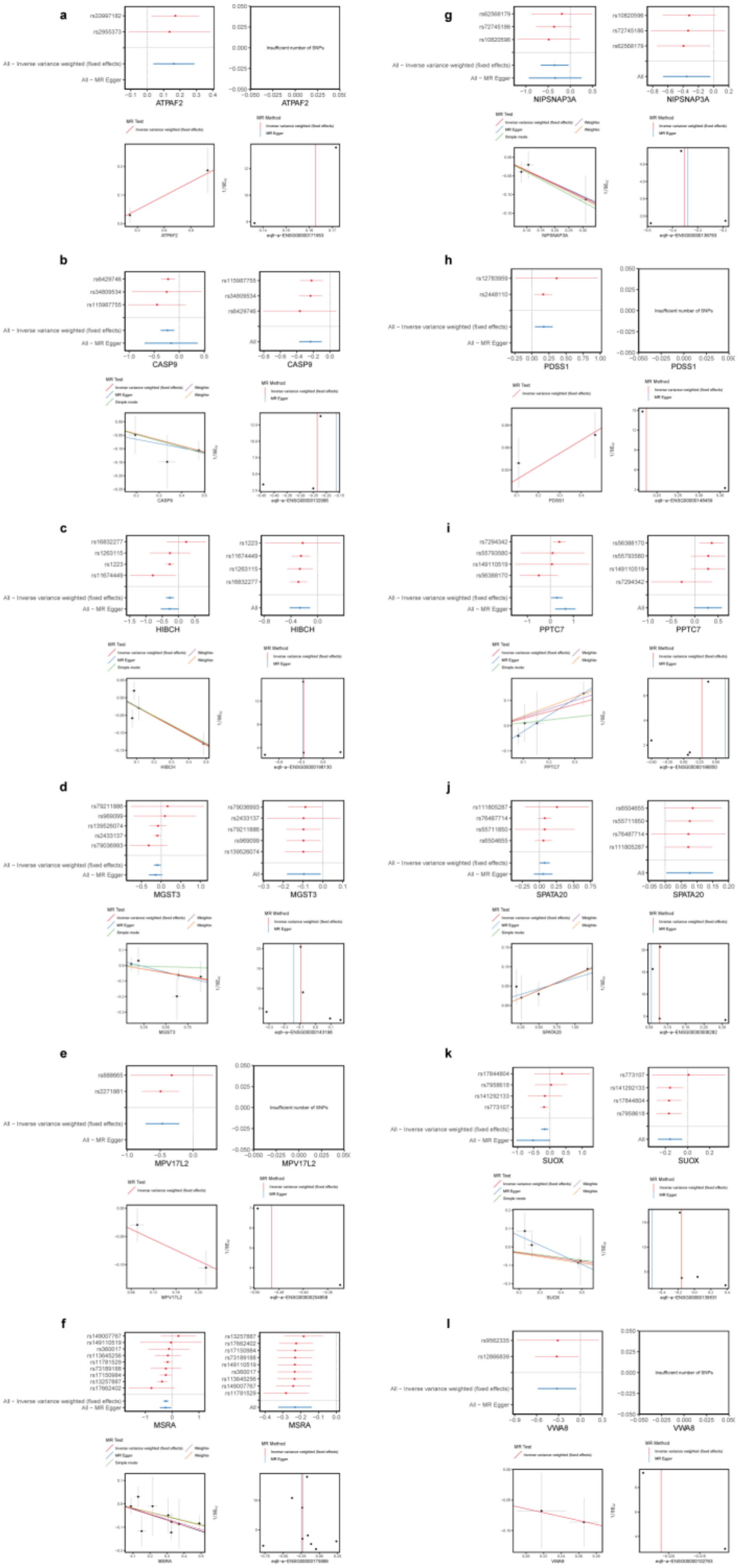

Supplementary Fig 3: Visualized Mendelian randomization analysis of the mitochondria-associated plasma protein pQTLs

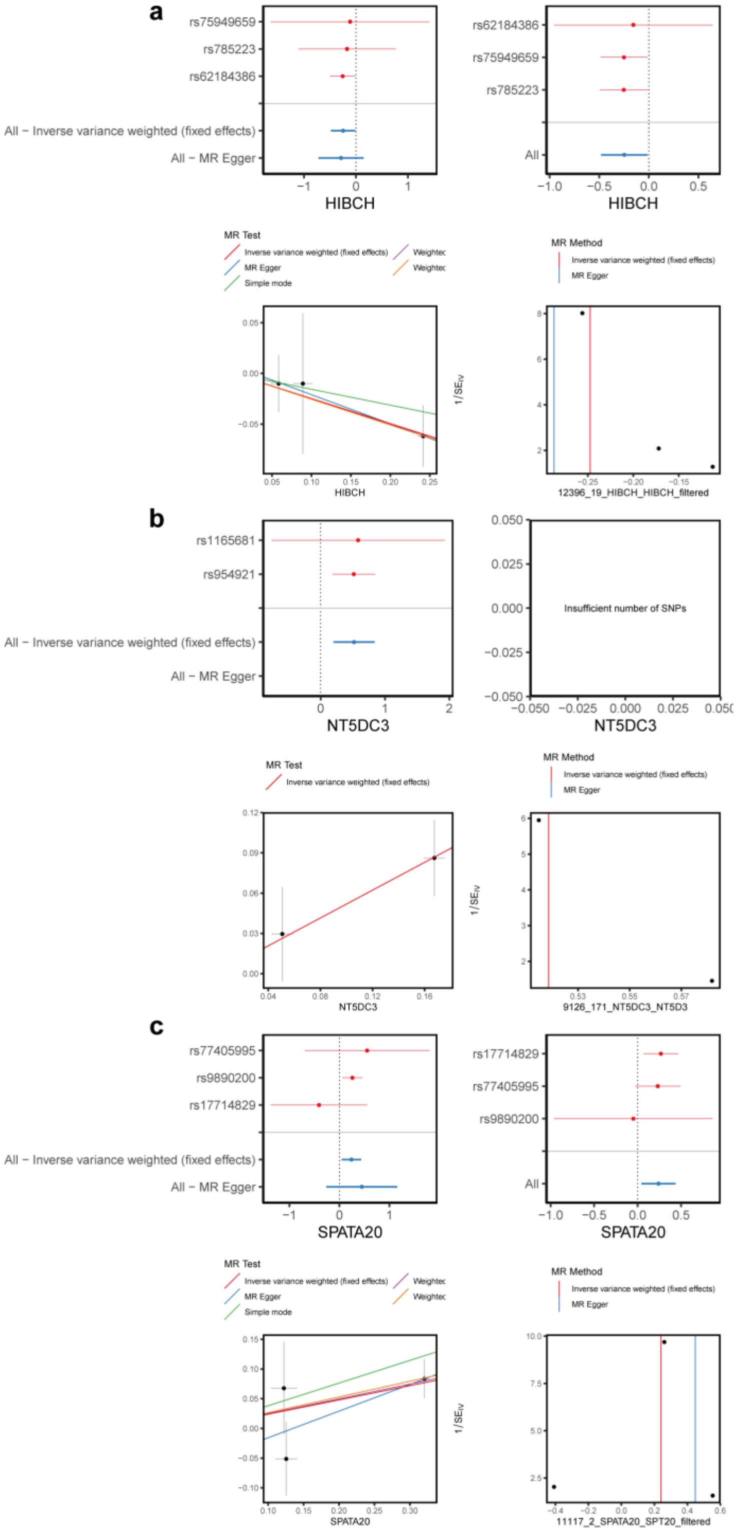

Supplement: Supplementary file 2 [file medi-104-e45301-s002.pdf]
